# Exploration of the Neuromodulatory Properties of Fyn and GSK-3β Kinases Exploiting 7‑Azaindole-Based Inhibitors

**DOI:** 10.1021/acs.jmedchem.5c00629

**Published:** 2025-08-08

**Authors:** Giambattista Marotta, Francesca Massenzio, Jose Ortega, Debora Russo, Ilaria Penna, Federico Falchi, Giorgia Babini, Sabrina Petralla, Rita Scarpelli, Elena Roggiolani, Gert Fricker, Michela Rosini, Andrea Cavalli, Barbara Monti, Anna Minarini, Filippo Basagni

**Affiliations:** † Department of Pharmacy and Biotechnology, 9296 Alma Mater Studiorum—University of Bologna, Via Belmeloro 6, 40126 Bologna, Italy; ‡ Department of Pharmacy and Biotechnology, Alma Mater Studiorum—University of Bologna, Via Selmi 3, 40126 Bologna, Italy; § Computational and Chemical Biology, 121451 Istituto Italiano di Tecnologia, Via Morego 30, 16163 Genova, Italy; ∥ D3 Pharma Chemistry, Istituto Italiano di Tecnologia, via Morego 30, 16163 Genova, Italy; ⊥ Medicinal Chemistry and Technologies for Drug Discovery and Delivery Facility, Istituto Italiano di Tecnologia, via Morego 30, 16163 Genova, Italy; # Institute of Pharmacy and Molecular Biotechnology, Ruprecht-Karls-University, Im Neuenheimer Feld 364, 69120 Heidelberg, Germany; ¶ IRCCS Istituto delle Scienze Neurologiche di Bologna, Via Altura 3, 40139 Bologna, Italy

## Abstract

The lack of efficient treatments and reliable biomarkers for neurodegenerative diseases requires the development of a late-stage powerful therapy. To this aim, we focused on Fyn and GSK-3β because both kinases are strictly involved in regulating neurodevelopmental processes, besides orchestrating neurotoxic aggregates’ deposition and neuroinflammatory processes development. Based on these premises, we developed dual kinase inhibitors to verify at the cellular level the suitability of Fyn and GSK-3β modulation in pursuing the recovery of neural trophism paired to the activation of a neuroprotective profile. Starting from the mild inhibitory potency of the 3-aminothiazole-7-azaindole scaffold, we identified nanomolar dual and selective inhibitors among the kinases of interest. In-depth biological evaluations were performed with the best compounds of the series to assess the neuroprotective and neuromodulatory properties, like enabling neurogenesis or glial polarization, as well as triggering immunomodulation with different patterns relating to their inhibitory profile, setting the stage for potential development of neuroregenerative treatments.

## Graphical abstract

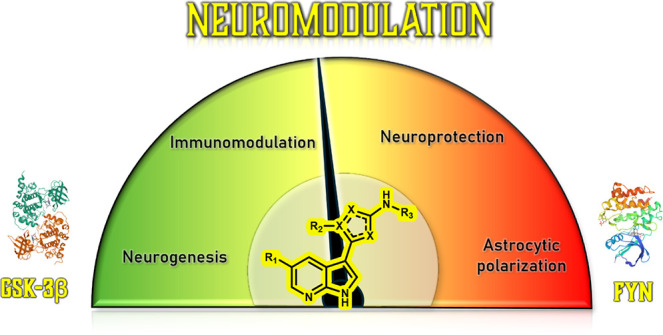

## Introduction

The intricate etiopathology of neurodegenerative diseases represents one of the biggest unmet medical needs, which results in a lack of efficient treatments able to halt the cognitive/motor deterioration. Impaired neurotransmission systems, as well as neurotoxic aggregates of misfolded proteins guided by neuroinflammatory processes jointly contribute to synaptic loss and neuronal death leading to neurodegeneration.^1^ The lack of reliable biomarkers for robust early diagnosis made the available treatments ineffective, as they usually intervene in already compromised neurodegenerative conditions.^2^ Therefore, the chance to step in with potential treatment able to merge neuromodulatory to neuroprotective properties could result in amplified therapeutic effect favoring the shift from neurodegeneration to neuroregeneration.^3,4^ Particularly, the pharmacological triggering of neurogenic and neurodevelopmental processes should contribute to arrest cognitive/motor decline while preventing neurodegeneration, with the establishment of an induced neuroprotective defense.

To this aim, Fyn proto-oncogene kinase (Fyn) and Glycogen synthase kinase 3β (GSK-3β) emerged as promising targets for their multiple involvement in modulating brain physiopathological conditions. Besides triggering and fostering the formation of neurotoxic aggregates and the development of inflammatory processes, both kinases are physiologically involved in synaptic plasticity and glia formation.^5^ Fyn is a nonreceptor tyrosine kinase belonging to the Src family involved in multiple neurodevelopmental processes, such as promoting myelination, mediating oligodendrocytes differentiation and maturation, and controlling neuronal migration and synaptic regulation.^6^ Fyn dysregulation represents a shared neurotoxic feature among neurodegenerative disorders.^7,8^ In common with GSK-3β, Fyn is one of the most responsible kinases for amyloid precursor protein (APP) phosphorylation, thus triggering amyloidogenic cleavage and tau hyperphosphorylation, favoring neurofibrillary tangles (NFT) deposition.^9^ Furthermore, Fyn acts as an essential cellular player in boosting amyloid beta peptide (Aβ) and α-synuclein-induced neurotoxic cascade, while its persistent upregulation during neuroinflammation paired with its physiological expression in microglia and astrocytes make it a major conduit for fostering inflammatory processes.^10^ It is therefore not surprising that several animal models with Fyn ablation reported significantly mitigated Aβ-induced mortality, inflammation, NFT reduction, and dopaminergic degeneration.^7,11^

GSK-3β is a serine/threonine kinase widely expressed in the brain and broadly recognized as a valid target to contrast neuroinflammatory and neurodegenerative processes due to its strict entanglement in protein dysregulation, with the following neurotoxic aggregates deposition, or as a pivotal inflammation regulator.^12,13^ Moreover, experimental evidence from several studies confirmed the direct involvement of GSK-3β signaling in neural development and neuronal polarization, offering prospects for potential GSK-3β-directed neuromodulating therapies.^14^ Specifically, its inactivation drives neural progenitor proliferation, migration, and differentiation.^15^ Furthermore, GSK-3β inhibitors proved to exert remarkable neuroprotective properties to multiple neurotoxic insults and anti-inflammatory activities through phosphorylation-mediated modulation of the different transcription factors or signaling pathways.^16^ Notably, GSK-3β has been also identified as one of Fyn upstream regulators during cellular antioxidant response.^17^

Based on these premises, we aimed to combine the structural features responsible for Fyn and GSK-3β inhibition in single chemical entities to potentially merge the neuromodulatory properties with neuroprotective activities derived from their modulation to validate a potential synergistic neuroregenerative approach. Albeit several Fyn or GSK-3β inhibitors have been developed and evaluated for neurodegenerative diseases treatment, unfortunately translation into clinical practice has not occured.^7,13,18^ In this study, for the first time dual Fyn/GSK-3β inhibitors were purposely developed and characterized for their neuromodulatory properties. To this aim, we started from the 3-aminothiazole-7-azaindole scaffold, endowed with moderate Fyn and GSK-3β inhibitory potencies, and following several rounds of structural refinement, we identified nanomolar dual inhibitors that were then evaluated in suitable bidimensional (2D) and 3D cell cultures for their neuroprotective and neuromodulatory activities. Additionally, once the structural features required for optimal modulation of two kinases within the same class of compounds were identified, selective Fyn or GSK-3β inhibitors were developed and exploited in biological investigation to clearly depict the specific input ascribed to the modulation of the two kinases for the resulting biological properties.

## Results and Discussion

### Structure–Activity Relationship

In this work, we selected the 3-aminothiazole-7-azaindole core as a suitable starting point for the identification of potential GSK-3β and Fyn inhibitors. In preliminary *in vitro* evaluation, compound **I** promisingly inhibited both kinases, albeit with unbalanced potency toward GSK-3β (Table 1). From these premises, we started a hit-optimization campaign by systematically modifying the three regions of hit compound **I** (i.e., amino group, thiazole, and azaindole cores) to define the structural requirements for the optimal inhibition of the target kinases. First, different aliphatic and aromatic substituents were introduced on the amino group of thiazole, generating compounds **1**–**8**. From this series, benzyl derivative **7** emerged as the best one in terms of balanced activity, while the other members of the series gave a weak inhibition, especially on Fyn (Table 1). Particularly, except for **7**, only compound **6** bearing the phenyl side chain maintained the inhibitory activities of **I**. Therefore, we evaluated a small set of different substituents on the aromatic core (compounds **9**–**16**, Table 1) without achieving satisfying balanced inhibitors. To note, only the replacement of the phenyl ring with *p*-*N,N*-dimethyl aniline or a *p*-aniline (compounds **15** and **16**, respectively) retained the selective GSK-3β inhibitory property of **6** and **I**, indicating that these substituents are better tolerated in this kinase. Afterward, we selected the more promising benzyl derivative **7** to explore the amino region. Initially, we focused on benzyl analogues **17**–**26** (Table 1) by introducing small functional groups in ortho-, meta-, and para-positions or by replacing the aromatic core with *N*-heterocycles (4-pyridine in **27**, 3-chloro-4-pyridine in **28**, 2-pyrimidine in **29**, and 2-pyrazine in **30**, Table 1). Among the selected substituents, only the *p*-nitro group (in compound **24**) led to an improved submicromolar inhibitory profile toward GSK-3β. Regarding the different heterocycles attached in the side chain, the location of a nitrogen atom was preferred in the para position as occurs in compound **27** (GSK-3β IC_50_ = 0.28 ± 0.07 μM; Fyn IC_50_ = 3.48 ± 0.68 μM), whose inhibitory potencies were boosted with the insertion of an ortho-chloro substituent achieving the first double-digit nanomolar inhibitor of the series (**28**, GSK-3β IC_50_ = 0.038 ± 0.006 μM; Fyn IC_50_ = 0.71 ± 0.09 μM). Finally, different modifications were also explored in the aminomethyl bridge, such as the elongation of the methylene chain (**31**), the blockage with an amide group (**32**), and capping into a tertiary amine function (**33**), which totally disrupted Fyn activity while GSK-3β seemed more tolerant with these modifications, except for amidation (Table 1).

**Table 1:**
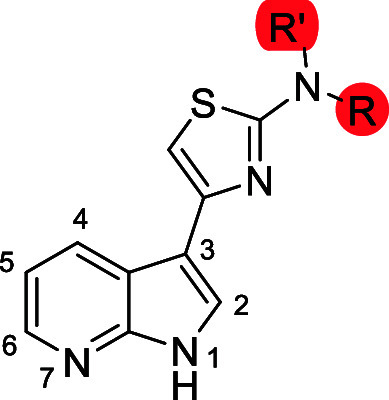

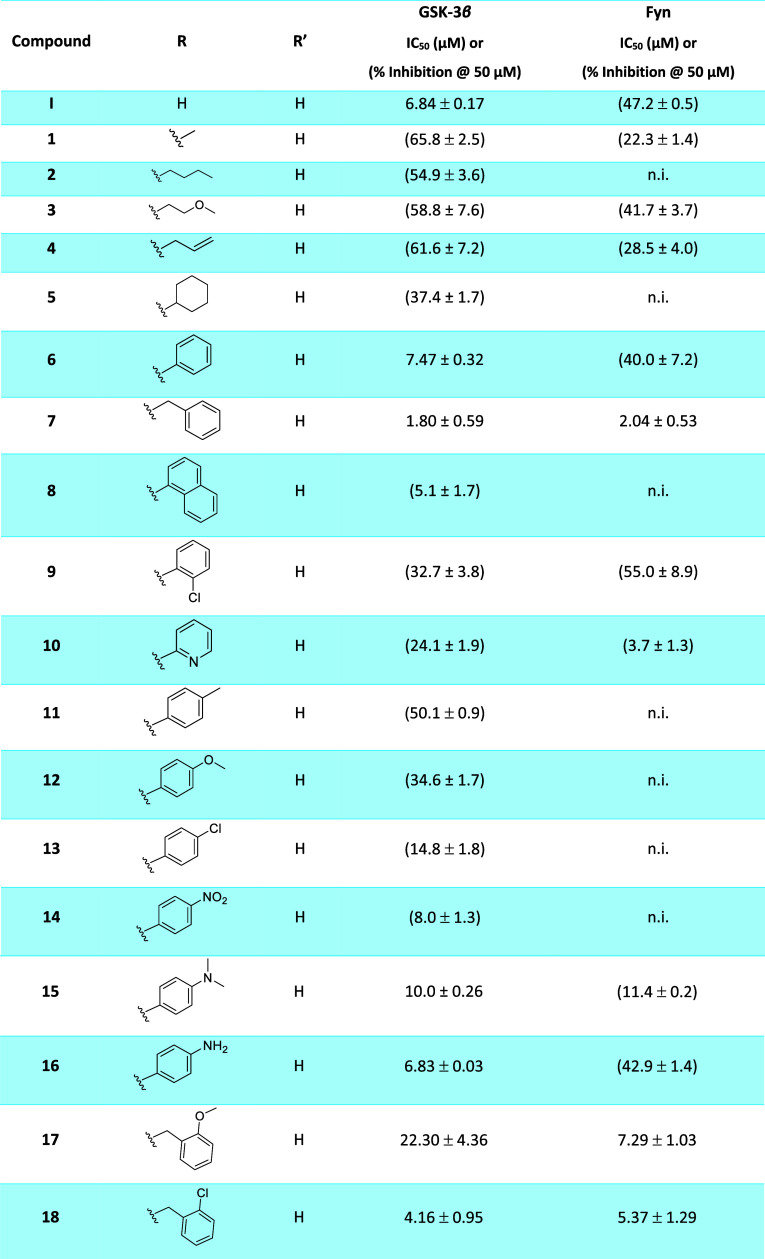

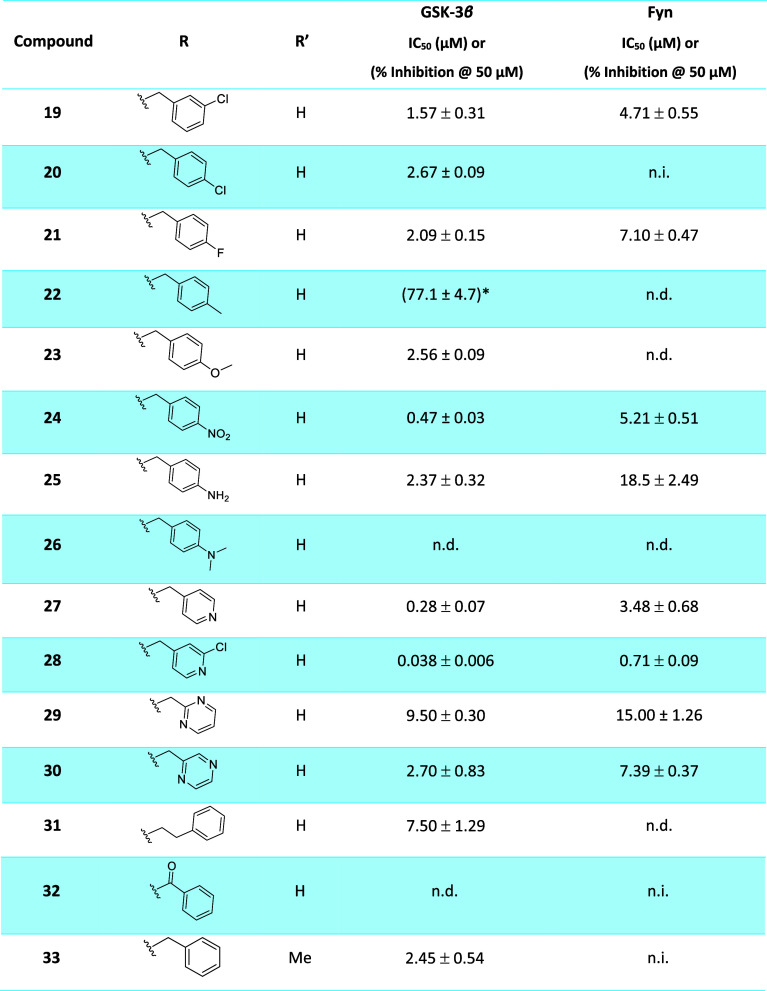
*In Vitro* Results of GSK-3*β* and Fyn Inhibition for Compounds **1**–**33** [Table-fn t1fn2]

a Percentages of inhibition and IC_50_ values are reported as a mean value (±SD) of at least three independent determinations. For most promising compounds, dose–response curves have been performed and IC_50_ determined. The percentage of inhibition at the highest dose tested (100 μM) is shown for compounds that did not reach a full dose–response for poor solubility in assay buffer (marked with *). n.i. = no inhibition. n.d. = not determined (IC_50_ was not calculated for compounds with a percentage of inhibition lower than 60% at 50 μM).

Then, we focused on the role of the 7-azaindole core in targets interaction. First, the removal, substitution, or masking of even one of the nitrogens led to a complete loss of activity toward the two kinases (compounds **34**–**37**, Table 2), highlighting the essential role of vicinal H-bonding acceptor–donor scheme in positions 1 and 7 for the inhibition. Surprisingly, the insertion of a bromine atom in position 5 (compound **38**, Table 2) increased the selectivity and inhibitory potency on Fyn in the submicromolar range (Fyn IC_50_ = 0.55 ± 0.02 μM).

**Table 2:**
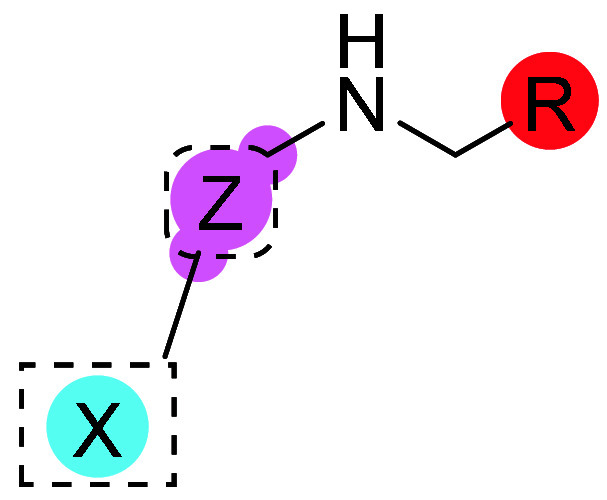

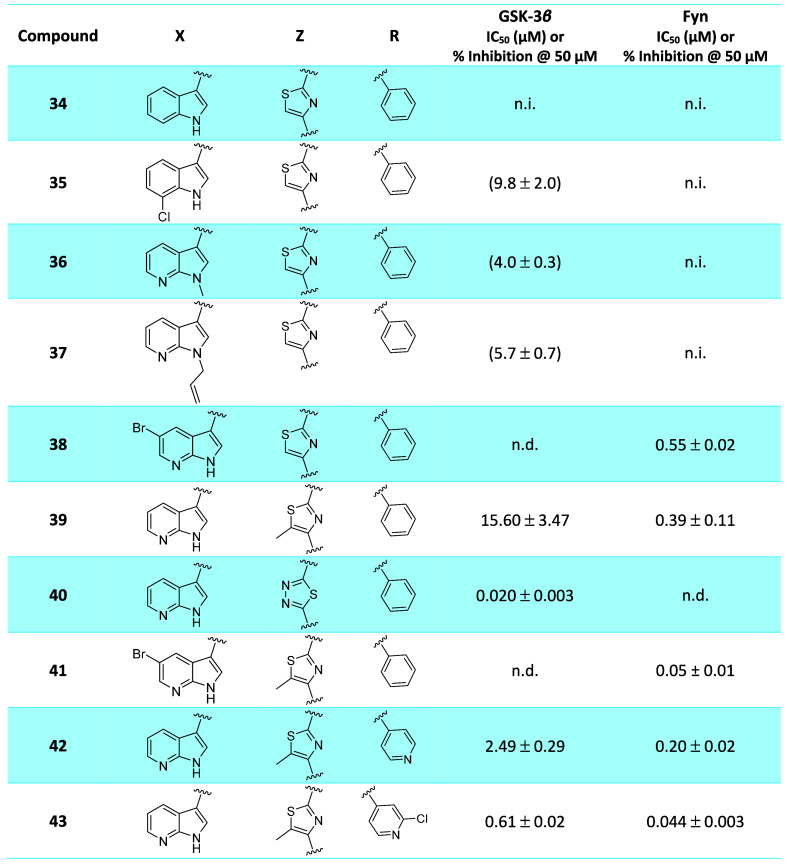
*In Vitro* Results of GSK-3*β* and Fyn Inhibition for Compounds **34–43** ^a^

a Percentages of inhibition and IC_50_ values are reported as a mean value (±SD) of at least three independent determinations. For most promising compounds, dose–response curves have been performed and IC_50_ determined. n.i. = no inhibition. n.d. = not determined (IC_50_ was not calculated for compounds with a percentage of inhibition lower than 60% at 50 μM).

Lastly, following the work conducted in the azaindole core, we explored two small thiazole modifications that were crucial for selectivity. Particularly, by simply adding a methyl group in position 5 (**39**, Table 2) the inhibitory activity was shifted toward Fyn (GSK-3β IC_50_ = 15.60 ± 3.47 μM; Fyn IC_50_ = 0.39 ± 0.11 μM), while 2-aminothiazole replacement with a 1,3,4-thiadiazol-2-amine nucleus powerfully switched the selective inhibition toward GSK-3β (**40**, GSK-3β IC_50_ = 0.020 ± 0.003 μM).

After this hit-optimization campaign, starting from the unbalanced micromolar inhibitor **I**, we achieved two dual compounds **27** (GSK-3β IC_50_ = 0.28 ± 0.07 μM; Fyn IC_50_ = 3.48 ± 0.68 μM) and **28** (GSK-3β IC_50_ = 0.038 ± 0.006 μM; Fyn IC_50_ = 0.71 ± 0.09 μM) with increased inhibitory potencies, albeit remaining more active toward GSK-3β. Furthermore, we identified a potent selective nanomolar GSK-3β inhibitor (**40**, GSK-3β IC_50_ = 0.020 ± 0.003 μM), while compounds **38** (Fyn IC_50_ = 0.55 ± 0.02 μM) and **39** (Fyn IC_50_ = 0.39 ± 0.11 μM) were the two more potent and selective Fyn inhibitors of the series. These results gave us preliminary information about the structural features required to modulate the activity on the two protein kinases. However, these activities were not yet satisfactory in terms of Fyn inhibition for both selective inhibitors **38** and **39** as well as dual compounds **27** and **28**. Based on these results, to boost Fyn-related selective potency, we therefore considered merging the two emerged Fyn-preferred motifs: bromine in position 5 of 7-azaindole nucleus with methyl group insertion in thiazole core. In this way, the two modifications were synergic for Fyn activity, achieving the more potent and selective Fyn inhibitor of the series (**41**, Fyn IC_50_ = 0.05 ± 0.01 μM). At the same time, to strengthen Fyn inhibitory potency of **27** and **28** the methyl group was inserted in their thiazole ring, affording compounds **42** and **43** (Table 2), respectively. In this case, Fyn IC_50_ values of both compounds dropped, while the GSK-3β IC_50_ value increased by an order of magnitude with respect to the parent compounds, highlighting GSK-3β′s low tolerance for this structural modification.

In the present study, we built upon a structure–activity relationship (SAR) investigation based on the different modifications made to the 3-aminothiazole-7-azaindole core regarding Fyn and GSK-3β activities, whose results are summarized in Figure 1: (i) the 7-azaindole core was pivotal for maintaining the inhibitory activities, whereas a 5-bromo substituent on this core enhanced Fyn inhibition; (ii) the insertion of a methyl group in position 5 of the central thiazole nucleus shifted the activity toward Fyn, while the replacement of thiazole with a thiadiazole ring switched completely the activity toward GSK-3β, rendering these modifications relevant to drive the selectivity within the two kinases; (iii) the side tail on the amino group was the most explored for chemical accessibility reasons and it was found that benzyl substitution was preferred and 4-methylpyridine with halogen insertion was also well tolerated.

**Figure 1:**
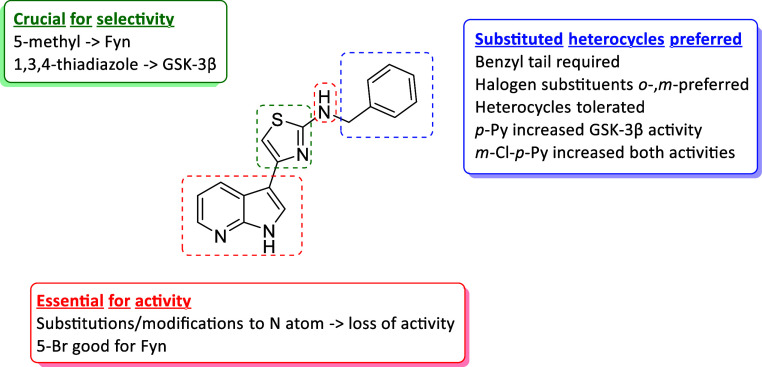
Schematic representations of the results of our SAR study.

### Mechanism of Action of Compound **43**

To gain insights into the mechanism of GSK-3β and Fyn inhibition within this class of compounds, compound **43** was tested for its ability to competitively replace ATP, as described in the Materials and Methods section.

Under a constant concentration (50 nM) of the substrates ULight-GS or TK (for GSK-3β and Fyn, respectively), ATP concentrations were varied from 0.75 to 12 μM or 1.5 to 24 μM (for GSK-3β and Fyn, respectively), and **43** was tested at 0.75 and 4.5 μM against GSK-3β and 0.05 and 0.3 μM against Fyn. Under these experimental conditions, we observed an increase in *K*_m_ constant (Michaelis–Menten constant) but an unaltered 1/*V*_max_ value, when the concentration of **43** increased (Figure 2), suggesting a competition between the compound and ATP.

**Figure 2:**
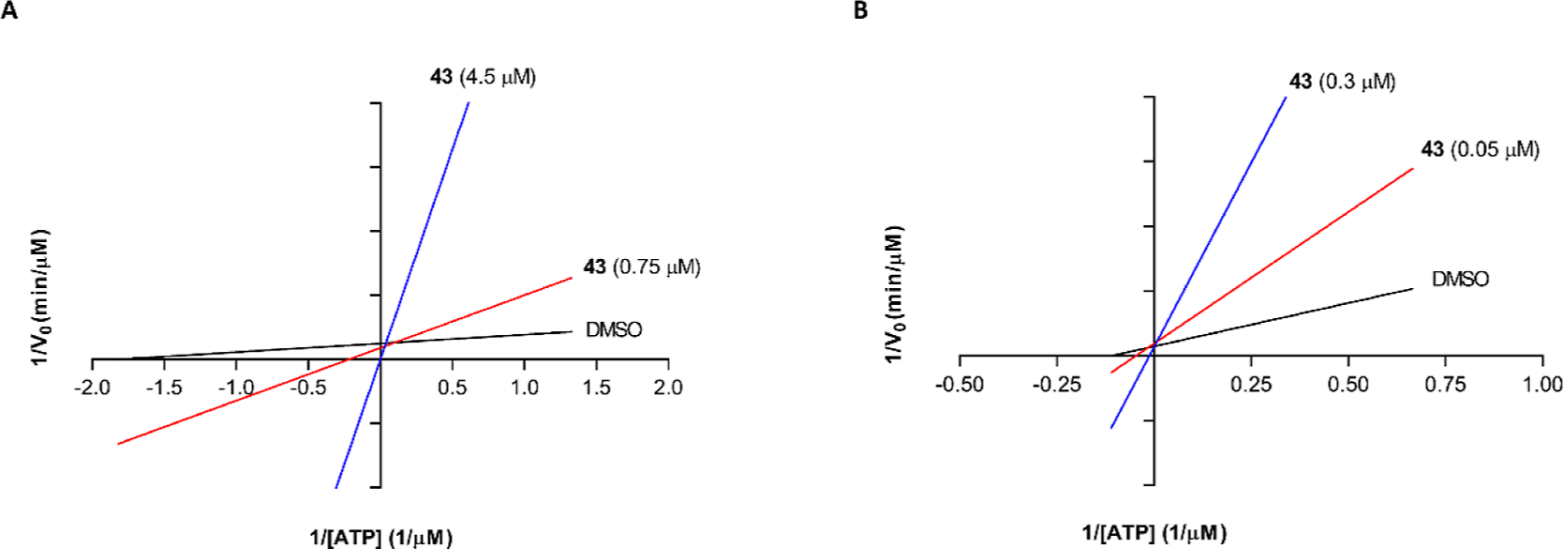
Lineweaver–Burk plots of GSK-3β and Fyn kinetic data (A,B) at two concentrations of compound **43** (0.75 and 4.5 μM, 0.05 and 0.3 μM, respectively). (A) Linear regression plot of 1/V against 1/[ATP] at given concentrations of the compound on GSK-3β and (B) on Fyn. Intersecting at the same point on the *y*-axis indicates competitive inhibition with respect to ATP of compound **43** on both targets.

### Computational Investigations

Based on the experimental outputs, we carried out molecular dynamics simulations on the complexes obtained from traditional docking of the most interesting compounds to rationalize at molecular level the differences of their inhibition profile toward the two kinases. Particularly, computational investigations revealed the ability of this series of compounds to properly accommodate within the ATP-binding sites of the two kinases, according to the ATP-competitive mechanism of inhibition determined by kinetic studies. As emerged from the complete inactivity of compounds **34**–**37**, the 7-azaindole nucleus acts as an anchor point with the hinge region of both targets through two vicinal H-bond with the amino acid backbone of Glu83 and Met85 in Fyn (Figures 4 and 5) or Asp133 and Val135 in GSK-3β (Figures 3 and 5). Moreover, for the whole series a third essential H-bond occurs with the amino group of aminothiazole and side chain of Asp148 in Fyn and Gln185, Asn186, or Asp200 via the H_2_O-bridge in GSK-3β (not shown in Figure 3). From computational investigations it emerged that the same compound adopts a different geometric orientation within the active site of the two kinases: a close conformation is required for Fyn due to a flattened binding pocket, while in GSK-3β the benzyl side chain is more free to openly rotate.

**Figure 3:**
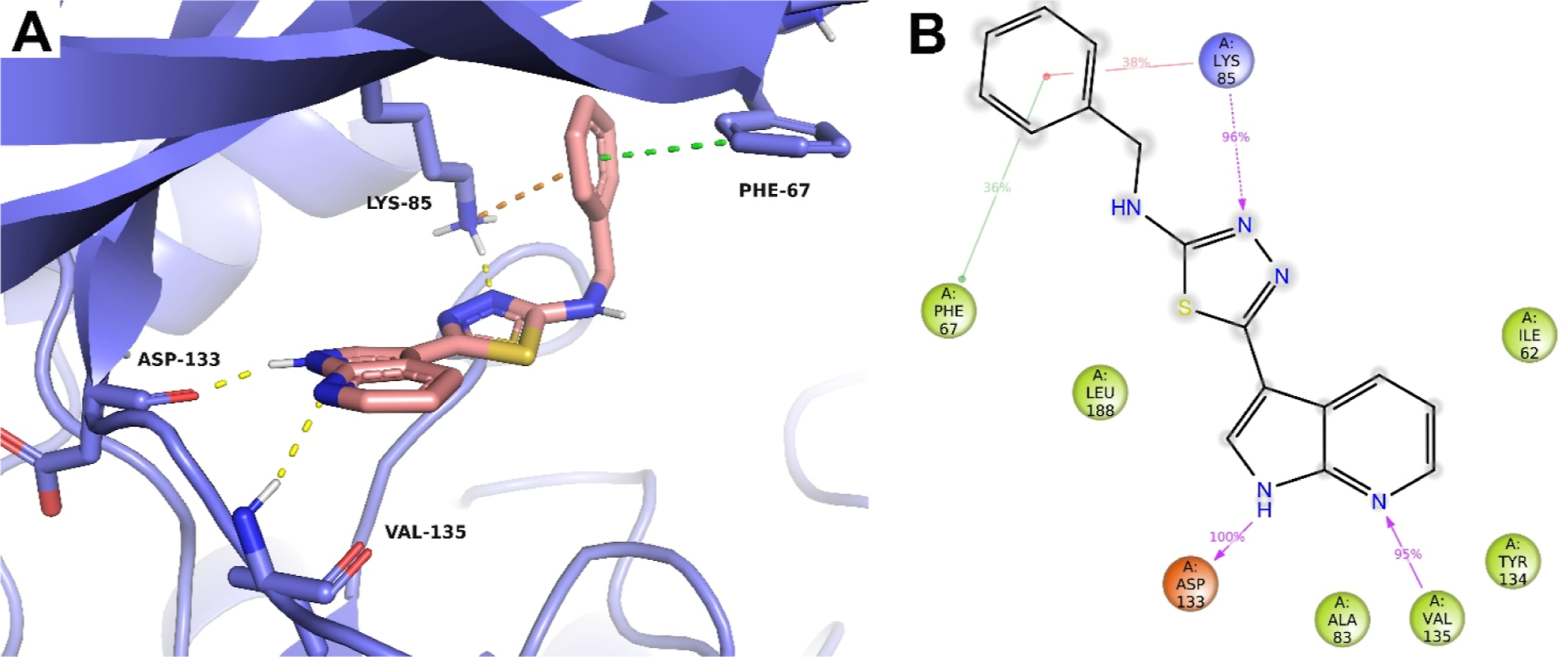
(A) Tridimensional binding pose of compound **40** within GSK-3β binding site with highlighted established interactions. Yellow dotted lines are H-bond interactions, orange dotted line is p-cation interaction, and green dotted line is p–p interaction. (B) Bidimensional interaction profiles over time calculated on the last 50 ns of molecular dynamics simulation. Green arrow is p–p interaction, red arrow is p-cation interaction, and magenta arrows are H-bond interactions. The number on the arrow represents how much of a percentage of interaction is maintained over the analyzed molecular dynamics time (last 50 ns).

The driving force in kinase selectivity relies on different thiazole substitutions. The conversion of this core in the thiadiazole (compound **40**) strongly enhances GSK-3β-related potency due to an additive H-bond between its N3 and the amino group of Lys85 alongside the benzyl group, which results to be sandwiched between a pi–pi interaction with Phe67 and a pi-cation with the same Lys85 (Figure 3). In parallel, the insertion of a methyl group on thiazole causes a steric clash with Leu132 in GSK-3β (reported in Figure 4B for compound **41**), forcing the molecule to be outside the binding site enough to lose crucial interactions for activity. Conversely, in Fyn the same substituent perfectly fits in a hydrophobic cavity while stabilizing compound **41** in a suitable pose for a more efficient H-bond between the amino group and Asp148 as well as the interaction between bromine on the azaindole ring, acting as an acceptor, and the H-bond donor in the Ser89 backbone (Figure 4A).

**Figure 4:**
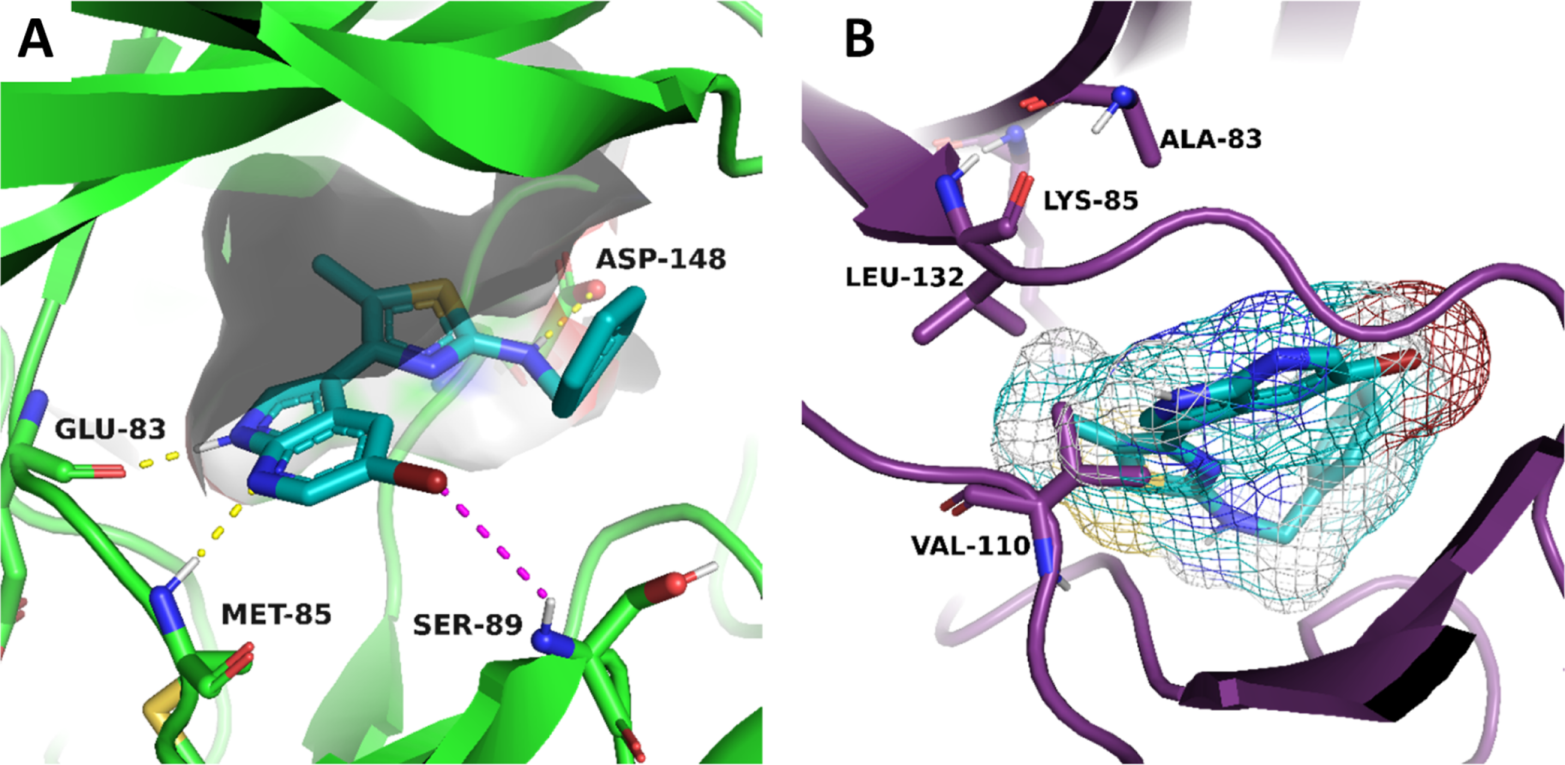
Tridimensional binding pose for compound **41** in Fyn with highlighted surface area of the binding pocket (A) and the forced pose potentially adopted within GSK-3β with the mesh representation of **41** surface pointing out the steric clash with side chain of Leu132 (B). Yellow dotted lines are H-bond interactions, and the magenta dotted line is the interaction between halogen and H-bond donor.

In recent years, the introduction of different halogen atoms in bioactive molecules has played a pivotal role for successful drug discovery campaigns by boosting target engagement and potency (besides pharmacokinetic improvements) thanks to their peculiar electronic properties.^19^ In our case, the essential role of halogen bonding within this class of compounds has been revealed for the first time in the Fyn potency shift while comparing compound **38** (Fyn IC_50_ = 0.55 ± 0.02 μM) with **7** (Fyn IC_50_ = 2.04 ± 0.53 μM) due to the new interaction between bromine atom in 5 and Ser89’s NH. Furthermore, the same effect resulted in increased potency toward both kinases after inserting a chlorine atom in the pyridine ring of compound **27** (GSK-3β IC_50_ = 0.28 ± 0.07 μM; Fyn IC_50_ = 3.48 ± 0.68 μM) vs **28** (GSK-3β IC_50_ = 0.038 ± 0.006 μM; Fyn IC_50_ = 0.71 ± 0.09 μM). Differently from the phenyl, the pyridine ring already better stabilizes the side chain by establishing several H-bonds via water bridges (Asp92 and Leu17 in Fyn, while Ile62 and Asn64 occur in GSK-3β beside a stronger pi–pi with Phe67 in the latter), but by adding the *o*-chloro an order of magnitude upgrade in potency occurred. Particularly, in Fyn the newly inserted chlorine atom interacts with the same Ser89 forming a halogen bond beside hydrophobic interactions with Leu17, while in GSK-3β the halogen bond engages the NH in Asn64’s backbone in addition to hydrophobic interactions with Ile62 and Val70 (Figures 5A,B and S1A,B).

**Figure 5:**
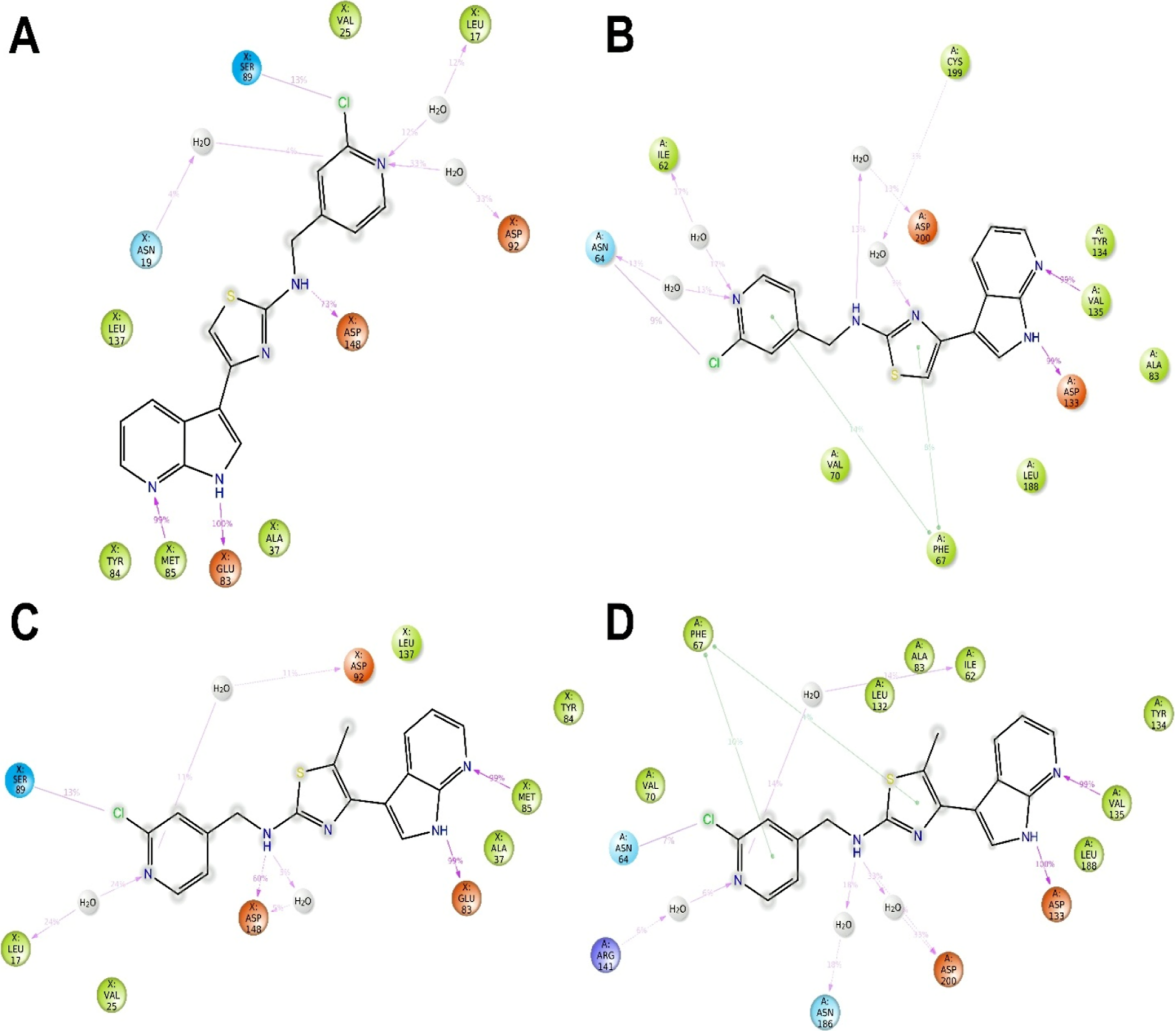
Bidimensional interaction profiles over time calculated on the last 50 ns of molecular dynamics simulation of compounds **28** (A,B) and **43** (C,D) within Fyn (A,C) and GSK-3β (B,D). Green arrows are p–p interactions, red arrows are p-cation interactions, magenta lines are halogen bonds, and magenta arrow are H-bond interactions. The number on the arrow represents how much of a percentage the interaction is maintained over the analyzed molecular dynamics time (last 50 ns).

Finally, the insertion of a methyl group in compound **43** allows again a perfect fixed placement within the Fyn binding site for optimal H-bond with Asp148 and Leu17 (Figures 5C and S1C). Conversely, it shows the same detrimental effect in GSK-3β accommodations by pushing our compound slightly out of the pocket and then permitting worse halogen and pi–pi interactions, which in this case were mitigated by the new H-bonds between Arg141 and pyridine and the secondary amino group with Asn186 via water bridges (Figures 5D and S1D).

### Biological Evaluation

Once the structural features responsible for the optimal modulation of the two kinases were identified, we aimed to evaluate the neuromodulatory profiles of the most promising compounds. Particularly, to possibly define the role of the two kinases in this context, we selected the two more selective compounds, **41** for Fyn and **40** for GSK-3β, along with the two more potent dual inhibitors **28** and **43** and the completely inactive compound **26** as negative internal control.

#### Neurotoxicity

A preliminary cell viability assessment was conducted in primary rat cerebellar granule neurons (CGNs), which are considered a reliable model for studying cellular and molecular mechanisms of survival/apoptosis and neurodegeneration/neuroprotection.^20^ Furthermore, primary cells provide higher quality models, and they are more sensitive to drug treatment than immortalized cell lines, as they form synapses and incorporate significant neuromodulatory and trophic inputs. CGN viability was assessed after 24 h treatment of compounds **26**, **28**, **40**, **41**, and **43** at clinically relevant concentrations (i.e., 5, 10, and 25 μM) using MTT assay. As reported in Figure 6, all compounds were not neurotoxic at tested concentrations paired with a slight increase in the registered cell viability in comparison to the control, with marked differences depending on the compounds. Particularly, this effect was amplified for Fyn inhibitors (i.e., **28**, **41**, and **43**). As a confirmation of the facts, a similar trend was also identified for other less active compounds (Figure S3).

**Figure 6:**
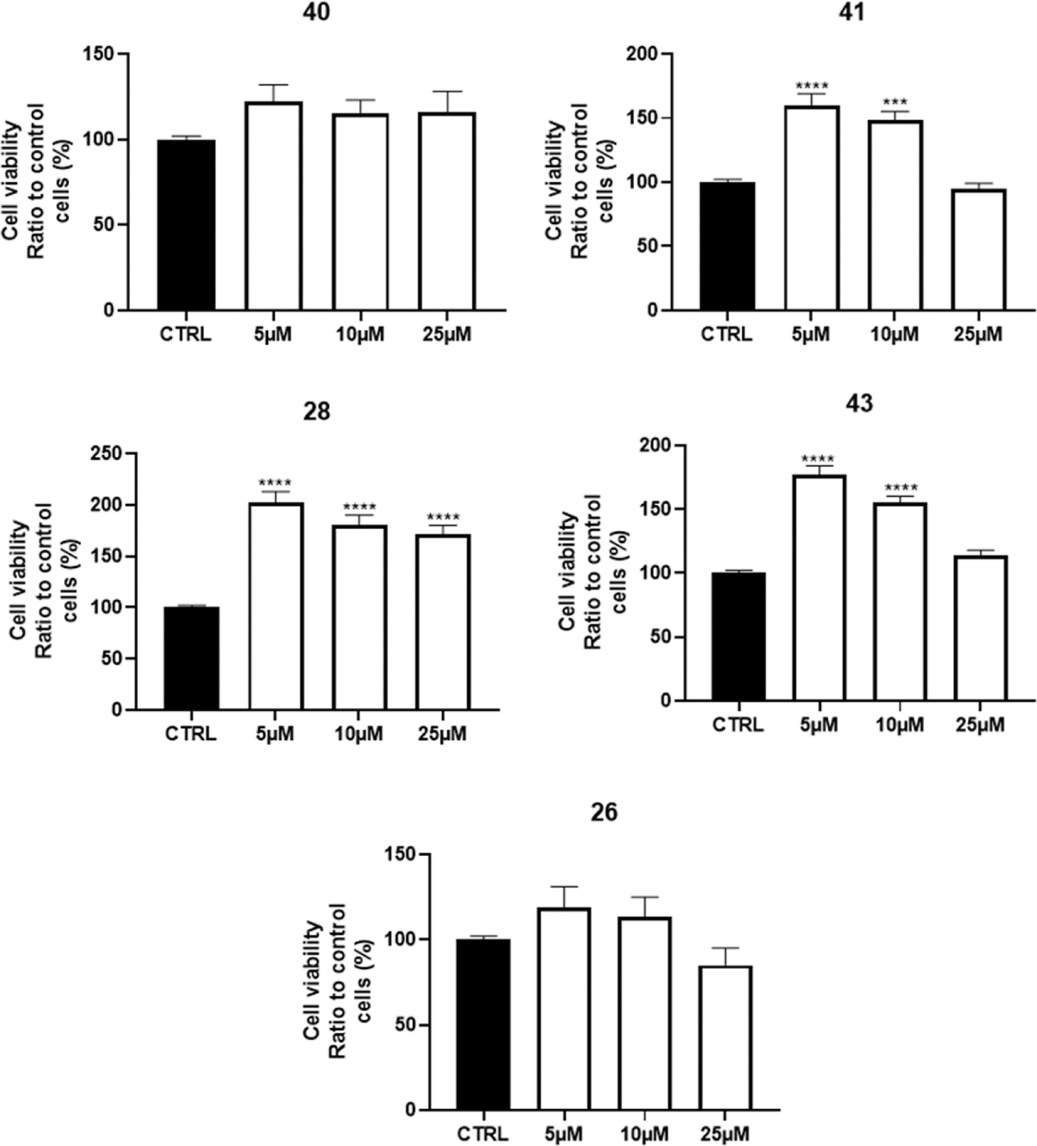
Neurotoxicity of the selected compounds **26**, **28**, **40**, **41**, and **43** at 5, 10, and 25 μM on CGNs, expressed as percentage of cell viability compared to control. *n* = 4; ***p < 0.001, ****p < 0.0001 vs CTRL, One-way ANOVA, Dunnett’s multiple comparison test.

This peculiar behavior could be accountable through the modulation of different cellular pathways, such as the reduction of the physiological cell mortality rate or stimulated hyperproliferation.^21^ To figure out the putative mechanism of action within this kinase inhibitor family, we carried out an in-depth biological investigation dissecting one by one the different neuromodulatory pathways.

#### Neuroprotection

First, we evaluated the neuroprotective properties of the selected compounds in the same cell line, once the expression of Fyn and GSK-3β in this cellular model was verified (Figure S2), after a serum/potassium deprivation experiment, which simulates physiological aging (and mortality), triggering around 20% of cell death.^22^ In particular, 7 days differentiated CGNs were exposed to serum-free BME medium with low extracellular concentration of K^+^ ions and treated at selected doses (i.e., 5, 10, and 25 μM) of compounds for 48 h. Neuronal viability was then determined with an MTT assay. After treatment, all compounds, except the inactive compound **26** and the GSK-3β selective compounds **40** (Figure 7) or **33** (Figure S4), not only completely rescue neurons vitality but even induce an important cell-viability increase. In this case, we verified the important neuroprotective profile for Fyn-active compounds, while GSK-3β selective inhibitors were almost equipotent to inactive compounds in this respect, suggesting that Fyn inhibition plays a crucial role in fighting neuronal aging.

**Figure 7:**
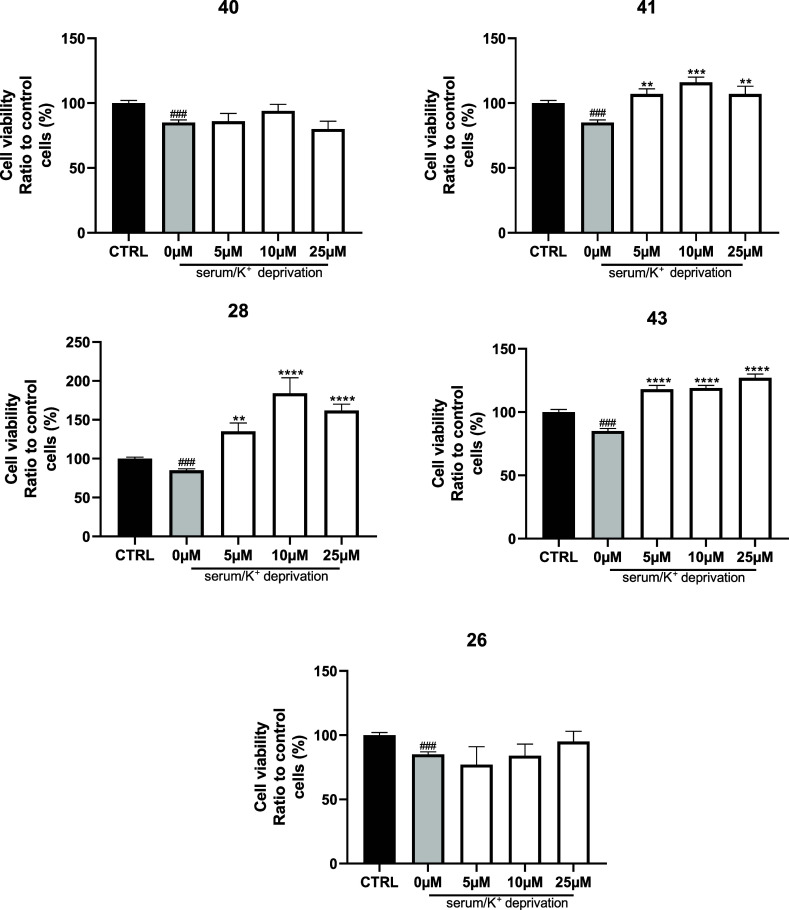
Neuroprotection of the selected compounds **26**, **28**, **40**, **41**, and **43** at 5, 10, and 25 μM on CGNs, expressed as percentage of cell viability compared to control. *n* = 4; * vs no serum no K^+^. One-way ANOVA, Dunnett’s multiple comparison test; # vs CTRL; unpaired *t*-test. **p < 0.01, ***p < 0.001, ****p < 0.0001 vs CTRL.

#### Neural Progenitor Cell Proliferation

Differently from embryonic development, postnatal neurogenesis is limited to some regional regeneration and cognitive function (e.g., in the subventricular zone and subgranular zone of the hippocampal dentate gyrus) with gradual loss of intensity with age. This fact paired with the development of neurodegenerative conditions dramatically decreases the number of existing neurons and limits the brain’s intrinsic capacity to generate new neurons for functional replacement, with the consequent drop in brain function.^23^ Notably, impaired neurogenesis in neurodegenerative diseases may result from reduced proliferation of neural stem cells (NSCs), defective differentiation, or a pathological shift in lineage commitment.^24^ Therefore, the chance to pharmacologically trigger neuroregenerative processes, by influencing both proliferation and fate determination of neural progenitor cells (NPCs), has emerged as promising tool to counteract neurodegeneration.^3,25^ Particularly, NPCs are multipotent neural cells able to proliferate and differentiate into neurons, astrocytes, and oligodendrocytes, and several kinases are considered as master regulators of such neurodevelopmental processes, among which a key role is played by GSK-3β.^14,26^

Based on that, we further investigated the neurogenic and differentiation potential of our kinase inhibitors in cell culture of neurospheres, which are floating pools of NPCs coming from the subventricular zone (SVZ) of six-month-old mice. Particularly, we first analyzed the variation, during 7 days, in number and size of neurosphere population with and without treatment. Neurospheres physiologically grew in number during the first 2–3 days, with a following decrease due to physiological death of those not grown, while afterward they increased in dimension as well as shaped branched neurites. Therefore, single neurospheres were plated and allowed to grow spontaneously after treatment with three different concentrations comparable to the inhibitory potency (i.e., 0.1, 1, and 5 μM) of compounds **26**, **28**, **40**, **41**, and **43** or DMSO as control for 7 days (Figures 8 and S5). Generally, the tested compounds exerted mild neurogenic activity, mainly stimulating early proliferation more than later maturation. Particularly, compounds with higher GSK-3β inhibition (i.e., **28** and **40**, Figure 8 and **27**, Figure S5) demonstrated pronounced neurogenesis, which remained notably significant up to the fifth day at 5 μM, suggesting a pivotal role in inhibiting this kinase to achieve this activity. A premature increase in dimension (i.e., 3–5 days) has been observed for **42** (Figure S5), while noticeable maturation at days 6 and 7 has been verified only for compounds **27** (Figure S5) and **28** (Figure 8). For instance, in Figure 9, the efficiency of treatment with compound **28** can be noticed in comparison with untreated and inactive controls in terms of the boosted number (at day 4) and dimension (at day 7) of neurospheres, while dual inhibitor **43** was not effective in this respect.

**Figure 8:**
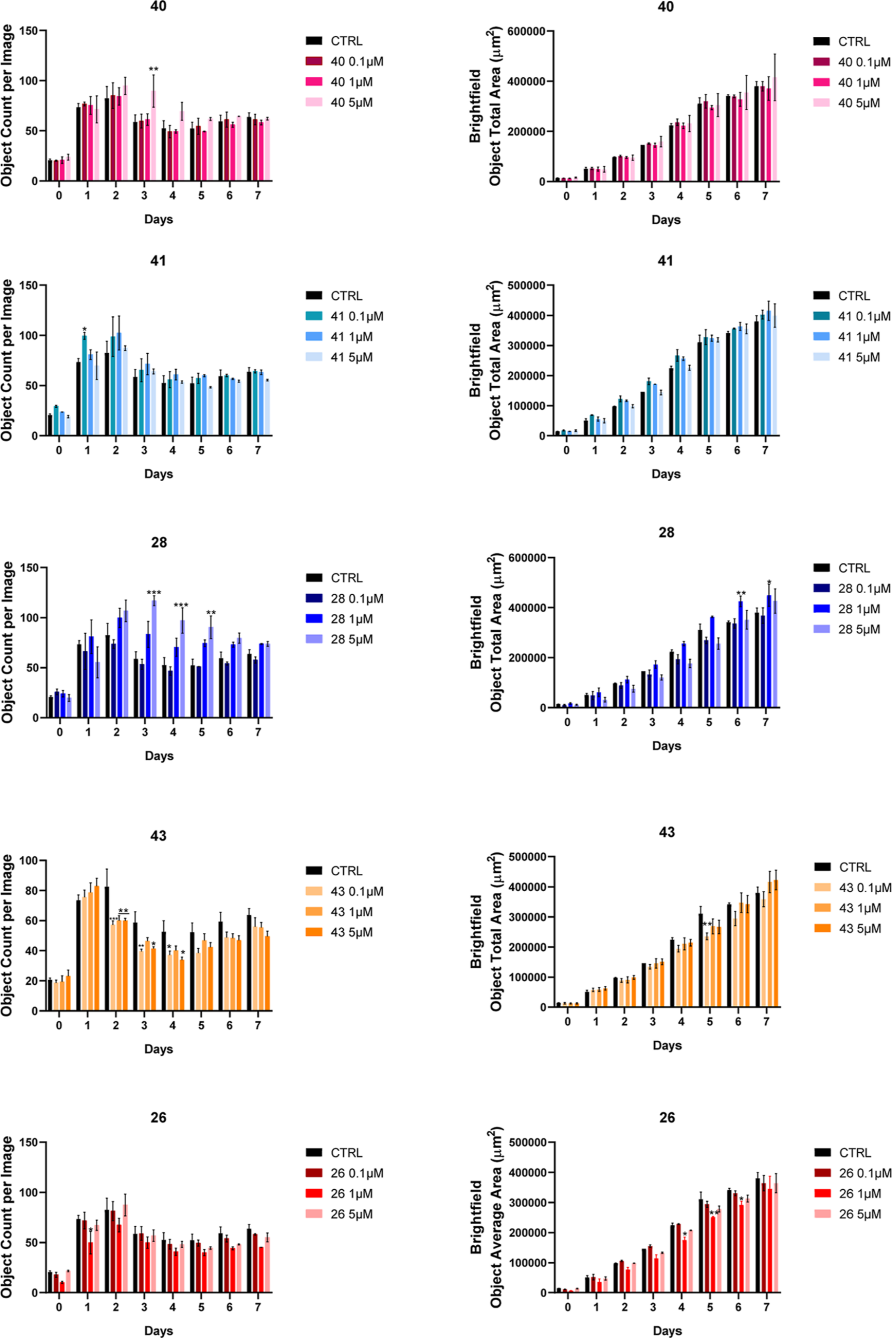
Growth analysis of neurospheres through the evaluation of the number of objects per image (on the left) and the total of the area of the brightfield object in the image (on the right). Single neurospheres were plated (5000 per well) and let spontaneously grow for 7 days. Tested concentrations: 0.1, 1, and 5 μM. Images were acquired every 24 h. *N* = 4 ± SE. Two-way ANOVA, Dunnett’s multiple comparison test. *p < 0.05, **p < 0.01, ***p < 0.001 vs CTRL.

**Figure 9:**
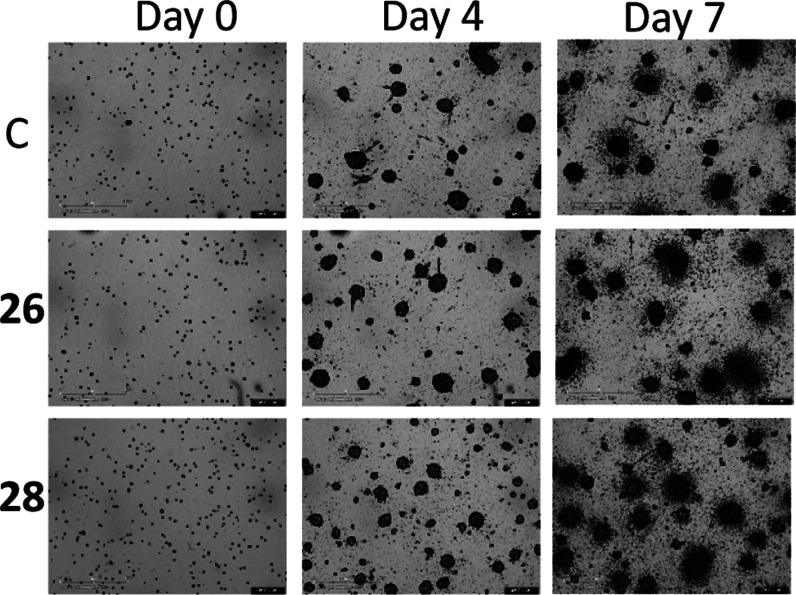
Brightfield images for days 0, 4, and 7 of compounds **26** and **28** at 5 μM vs control.

#### Neural Progenitor Cell Differentiation

Lastly, we move forward to evaluate the neuromodulatory profile of selected compounds that involves the capability to polarize the spontaneous differentiation of neurospheres, which potentially can maturate in neurons or macroglia, such as astrocytes and oligodendrocytes. Modulating NPC differentiation is especially relevant under neurodegenerative conditions as it could offer an intriguing opportunity to achieve polyhedric therapeutic effects. Importantly, neuronal differentiation may help to counteract the progressive neuronal loss, while promoting oligodendrocyte phenotype could support remyelination and neuronal function and the astrocytic polarization may aid to foster a neuroprotective profile.^27^ Particularly, after 7 days of treatment at 1 μM, in the same experimental conditions previously reported, cells were fixed and stained with different antibody markers such as doublecortin (DCX) for the immature neurons, glial fibrillary acidic protein (GFAP) for astrocytes, and oligodendrocyte transcription factor 2 (OLIG2) for oligodendrocyte precursors for immunofluorescence analysis (Figure 10a). Differently from other GSK-3β inhibitors,^15^ our compounds did not show a marked polarization toward a neuron-specific phenotype, except for a mild increment observed after treatment with GSK-3β selective inhibitor **40**, suggesting that the simultaneous Fyn inhibition plays a detrimental role in this respect. Instead, Fyn-active inhibitors induced a specific glial differentiation, mainly astrocytic, while a potent and selective Fyn inhibition (e.g., compound **41**, Figure 10b) was markedly deleterious for oligodendrocytes due to the previously cited pivotal role of Fyn in oligodendrocytes maturation.^28^ To note, this effect was mitigated in response to dual inhibitors **28** and **43** thanks to the beneficial role of simultaneous GSK-3β inhibition in this respect.^29^ Particularly, compounds **41** and **43** (i.e., selective and dual more potent Fyn inhibitors, respectively) notably directed the differentiation toward the astrocytic phenotype, promoting themselves as potential derivatives for counteracting neuroinflammatory processes. By driving the maturation of astrocytes, compounds **41** and **43** could exert neuroprotection through multiple mechanisms, including the secretion of neurotrophic factors, enhancement of antioxidant defenses, regulation of water balance, ion homeostasis, and glutamate buffering and recycling.^30^ Furthermore, astrocytes support the integrity of the blood–brain barrier (BBB) and aid in the refinement of neural networks via synaptic pruning, further reinforcing their role in preserving brain function and structure.^31^ Finally, besides a soft neuron polarization, compound **40** negatively affected astrocytic differentiation and proved to be inactive regarding oligodendrocytes, highlighting the important role of Fyn inhibition positively in the first case and adversely in the latter.

**Figure 10:**
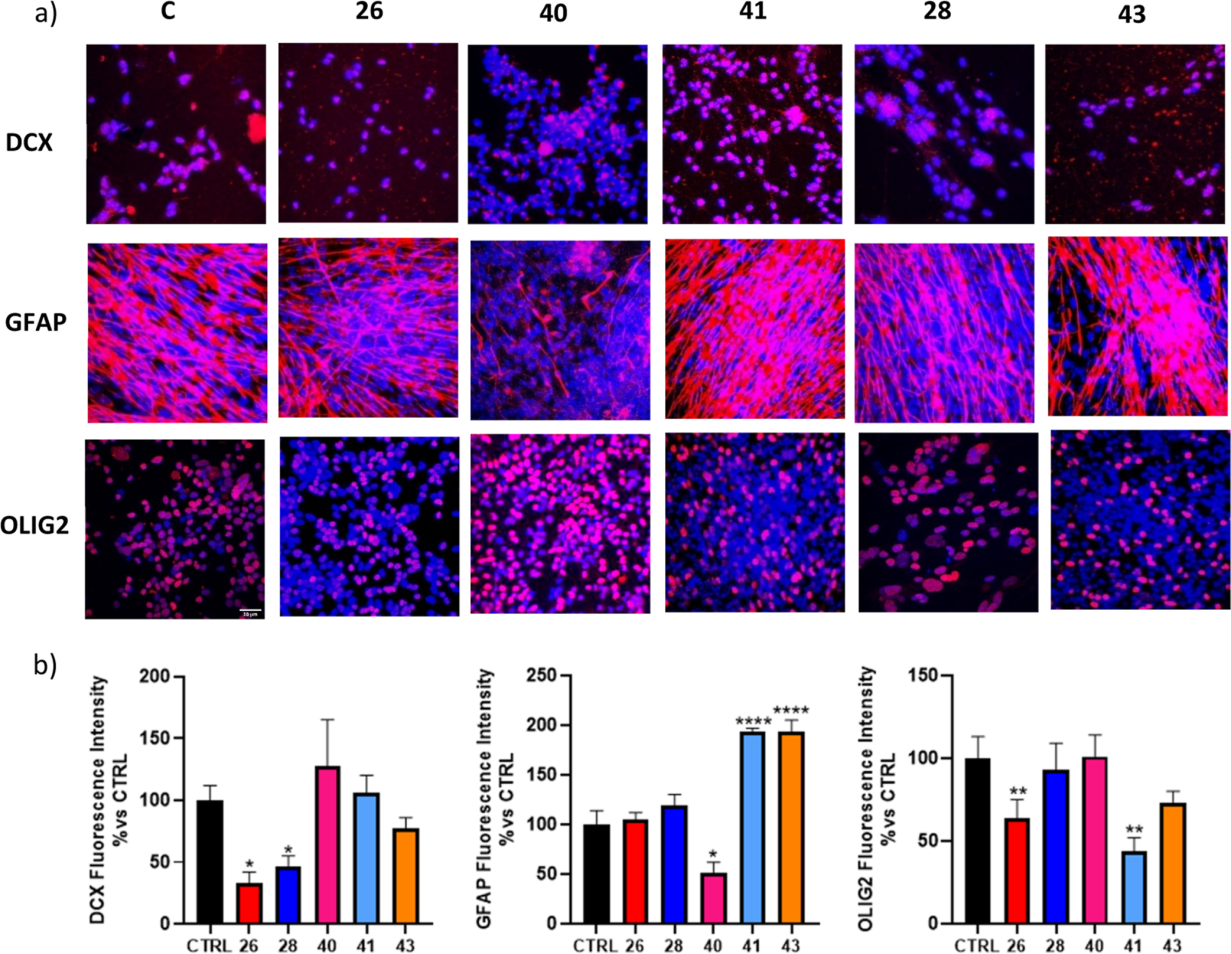
Immunostaining (a) and fluorescence intensity analysis (b) with differentiation markers on spontaneously differentiated neurospheres in the presence of the selected compounds **26**, **28**, **40**, **41**, and **43** (1 μM) or DMSO (control). *N* = 3 ± SE with 3 different fields acquired for each experiment. One-way ANOVA, Dunnett’s multiple comparison test. *p < 0.05, **p < 0.01, ****p < 0.0001 vs CTRL.

#### Immunomodulation

Microglia and astrocytes activation represents the main defense for fighting neuroinflammation in CNS.^32^ After identification of an inflammatory toxic insult, a coordinated and synergic astrocyte-microglia anti-inflammatory response occurs, upon activation, mainly through the modulation of cytokine trafficking as well as a final coordinated deescalation until the resting state. Furthermore, while astrocytes carry out even essential functions to maintain CNS brain homeostasis besides as immune checkpoint supervisors, microglia are considered the main immune effector cells of the brain.^33^ To gain a conclusive answer on the potential role of these kinase inhibitors in counteracting neuroinflammatory processes, after the verified induced astrocytic polarization, the ability of selected compounds to modulate inflammatory response was analyzed by favoring the immunomodulatory switch from neurotoxic M1 phenotype of microglia to phagocytic and neuroprotective M2 phenotype, evaluating inducible nitric oxide synthase (iNOS) and TREM2 as M1 and M2 markers (Figure 11). Particularly, M1-like is activated by inflammatory stimuli and further fuels the inflammatory cascade by increasing proinflammatory molecules such as iNOS. Conversely, M2-like phenotype increases neuroprotective functions through the production of anti-inflammatory cytokines and phagocytosis’ markers.^34^ In this regard, Fyn and GSK-3β inhibition has already been proven to be a promising immunomodulatory strategy.^35^ To this end, compounds **26**, **28**, **40**, **41**, and **43** were tested in immortalized murine microglia N9 at 2.5 and 5 μM, slightly higher concentrations with respect to differentiation studies in neurospheres. This is due to the low level of Fyn detected in this cell line and the different sensitivity and responsiveness of the two proposed cellular models (Figure S2). The tested compounds generally led to a decrease of proinflammatory iNOS as a marker of M1 phenotype, except for inactive kinase inhibitor **26** and GSK-3β-selective **40**. In detail, Fyn-selective **41** and dual inhibitor **28** notably decreased the M1 marker at both tested concentrations, while dual inhibitor **43** significantly reduced the M1 marker only at the highest concentration, suggesting a preferential Fyn inhibition for pursuing anti-inflammatory activity. Parallelly, despite some fluctuations, no significant variations of M2-marker occurred, highlighting the potential of these compounds to modulate glial inflammation and potentially revert proinflammatory phenotype, preserving the phagocytic properties of microglia. Altogether, these experimental outputs suggest the ability to modulate neuroinflammation by interfering with the microglial M1/M2 switch, rather than totally repressing microglia reactivity.

**Figure 11:**
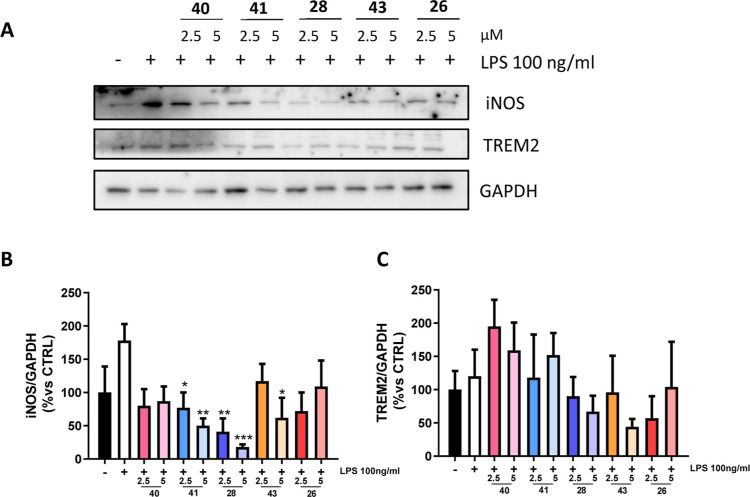
Immunomodulatory effects of the selected compounds **26**, **28**, **40**, **41**, and **43** at reported concentrations in N9 cells were evaluated through Western blot analysis of microglial polarization markers expression (A), with the respective densitometries of iNOS (B) and TREM2 (C) expressions. GAPDH was used as loading control. Densitometric results are expressed as percentage vs cells treated with LPS only and are the mean ± SE of three different experiments (Student’s *t*-test). *p < 0.05, **p < 0.01, ***p < 0.001 vs LPS.

### BBB Permeability

Blood–brain barrier (BBB) crossing represents one of the main criticisms during preclinical development of CNS-targeting drugs. Its continuous and not fenestrated endothelium paired with the presence of several efflux pumps (e.g., *P*-glycoprotein) strongly reduced brain permeability, therefore limiting drug uptake at effective doses in CNS.^36^ In this context, we preliminarily evaluated the physicochemical properties of the two most promising compounds of the series, acting as nanomolar dual inhibitors with immunomodulatory, neurogenic properties for **28** and astrocytic polarizing effect for **43**, in addition to the neuroprotective activities for both compounds (Table S1). If compared with the optimal parameters for CNS drugs,^37^ both compounds almost perfectly fitted within this benchmarks and were in accordance with those of CNS penetrating kinase inhibitors,^38^ with the only exception of a not enough basic profile. Furthermore, to corroborate their potential activity at the central level, we experimentally assessed the BBB permeation of these two compounds by means of a validated *in vitro* model (Figure 12, Table S2). To this aim, the immortalized human brain endothelial cell line (*h*CMEC/D3) was used since it mimics the brain endothelium-restricted permeability and expresses most of the transporters and receptors present in vivo, constituting a valuable *in vitro* model of the human BBB crossing system. Based on a reported protocol,^39^ we exploited the fluorescent dye tetramethyl-rhodamine isothiocyanate (TRITC)-dextran as a marker of passive diffusion through cell–cell junctions, where low TRITC-dextran permeability indicates the integrity of the endothelial monolayer. To note, low permeability to the fluorescent dye was retained after the permeability assays of TRITC-dextran with the tested compounds, confirming that all of them did not influence the endothelial monolayer integrity at a concentration of 100 μM (data not shown). CNS-directed drugs such as antipyrine and donepezil were used as positive controls, while the oligosaccharide inulin was used as a negative control, as well as TRITC-dextran. Compound **43** displayed a prominent apparent permeability (Papp 10^–6^ cm/s) of 32 ± 5.38, which is higher than the two positive controls employed. However, compound **28**, which differs in having only one methyl group less, showed a moderate BBB permeability with an endothelial Papp of 12.4 ± 1.13, almost to the same extent of donepezil (Papp of 15.6 ± 3.1).

**Figure 12:**
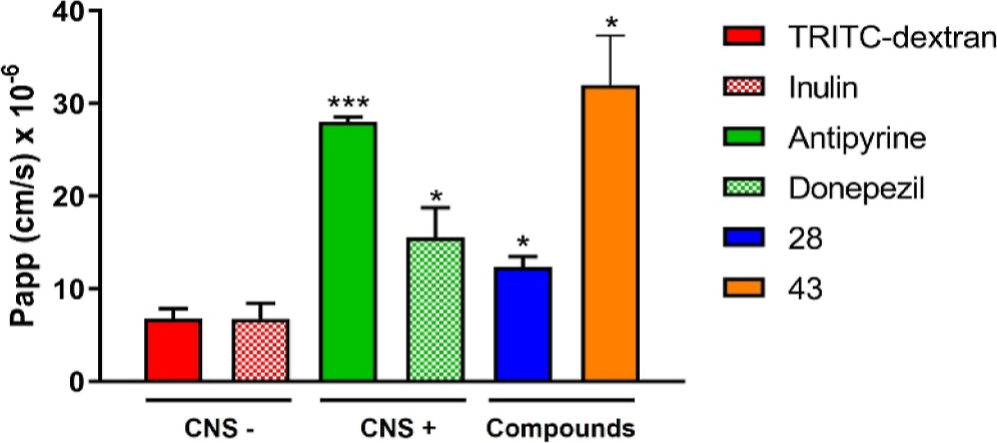
Prediction of the BBB penetration expressed as the permeability coefficient (Papp, cm s^–1^) ± SE. Antipyrine and donepezil were used as positive controls, while TRITC-dextran and inulin as negative controls. *p < 0.05, ***p < 0.001 vs TRITC-dextran; Student’s *t*-test.

### Kinase Selectivity Profiling of Compounds **41** and **43**

Finally, we selected compounds **41**, to fully evaluate its potential because of the lack of selective Fyn inhibitor to date, and **43**, due to its promising biological activities, for a wider kinase selectivity profiling. Particularly, both compounds were evaluated against a panel of 58 human wild-type kinases, representative of the different families covering the kinome, at 10 μM through the Eurofins (Eurofins Cerep, Celle l’Evescault-France) KinaseProfiler platform (Table S3). Notably, both compounds reaffirmed the inhibitory pattern toward Fyn or GSK-3β and revealed promising selectivity, with compound **41** confirming a more restricted selectivity profile compared to compound **43**, which was properly designed to target two kinases belonging to two different families. Interestingly, both compounds at tested concentrations, besides Src family members Fyn and Lyn, completely inhibited LOK and Abl kinases. Particularly, this latter is considered an important mediator of several neurotoxic pathways (e.g., neuroinflammation, oxidative stress or tau, Aβ and α-syn aggregates deposition) and multitarget inhibitors of Src/Bcr-Abl family kinases have already proven significant neuroprotective properties (i.e., dasatinib).^40^ Albeit the role of these different kinases in this respect has to be further investigated, Abl inhibition can confer specific beneficial effects to the herein disclosed kinase inhibitors. Furthermore, other main off-targets (>90% inhibition) for compound **41** turned out to be DRAK1 and MLK1 kinases, while for compound **43** emerged MSK2, Rsk1, CDK9 and PKA, which mainly represented understudied kinases or related to contradicting roles in neurodegenerative processes. To note, some of them (e.g., MLK1, CDK9) were involved in regulating inflammatory pathways, thus their inhibition could contribute to the resulting anti-inflammatory profile of our inhibitors.^41^

### Chemistry

Compound **I** was synthesized as previously reported.^42^ Almost all of the final compounds **1**–**43** were obtained following the same two-step procedure, with variations depending on the introduced substituents. The initial regioselective Friedel–Crafts acylation with bromoacetyl bromide (or 2-bromopropionyl bromide for intermediates **75** and **76**, Scheme 3) activated position 3 of the selected nitrogen-based bicycle: 7-chloroindole for intermediate **73** (Scheme S6), 5-bromoazaindole for **74** and **76** (Scheme 3), 1-substituted 7-azaindole for **71**–**72** (Scheme S5), and unsubstituted 7-azaindole for all the rest. The following Hantzsch cyclization with the proper *N*-substituted thioureas allows us to obtain final aminothiazole derivatives as hydrobromide salts. Where available, we directly exploited commercial thioureas (such as for compounds **1**–**15**, **20**, **22**, and **23**, Scheme 1), otherwise we prepared the required thioureas exploiting a modified three-step microwave-assisted reported procedure (Scheme 2).^43^ Reaction between benzoyl chloride, ammonium thiocyanate, and appropriate amine led to protected thioureas passing through benzoyl isothiocyanate intermediate. Consequent hydrolysis under basic conditions (except for nitro derivative **49**, where acidic conditions were required, and for benzamide **57**, where needed thiourea was directly ready after ammonia addition) resulted in thioureas **45**–**57** which underwent cyclization with **44** to achieve final compounds **17**–**33** (Scheme 2). All of the required amines for thioureas preparation were commercially available with the only exception of **58** which was obtained from the reduction of the corresponding nitrile (Scheme S1). To obtain aniline derivatives **16** and **25**, the nitro group of benzoylated thioureas **59** and **64** was reduced in the presence of iron under acidic conditions and, building on that, final phenyl and benzyl aminothiazole derivatives were obtained following the previously reported procedure (Schemes S2 and S3); furthermore, regarding **16**, Boc-protection of the amino group was needed during cyclization step to obtain a sufficiently pure final compound after carbamate deprotection (Scheme S2). In the case of indole **34** (Scheme S4), the preparation of 2-bromo acetyl chain in position 3 required a two-step strategy: a first acylation with acetyl chloride followed by α-bromination with copper­(II) bromide. For 1-functionalized derivatives **35** and **36** (Scheme S5) the initial N-substitution with proper alkyl halides and sodium hydride, as base, was followed by the same acylation and aminothiazole ring closure. Finally, cyclization of benzyl thiosemicarbazide **78**, obtained from hydrazine addiction to benzyl isothiocyanate, with 7-azaindole-3-carbonitrile **77**, prepared from 7-azaindole-3-carboxaldehyde and hydroxylamine, furnished the 2-amino-1,3,4-thiadiazole core of compound **40** (Scheme 4).

**Scheme 1:**
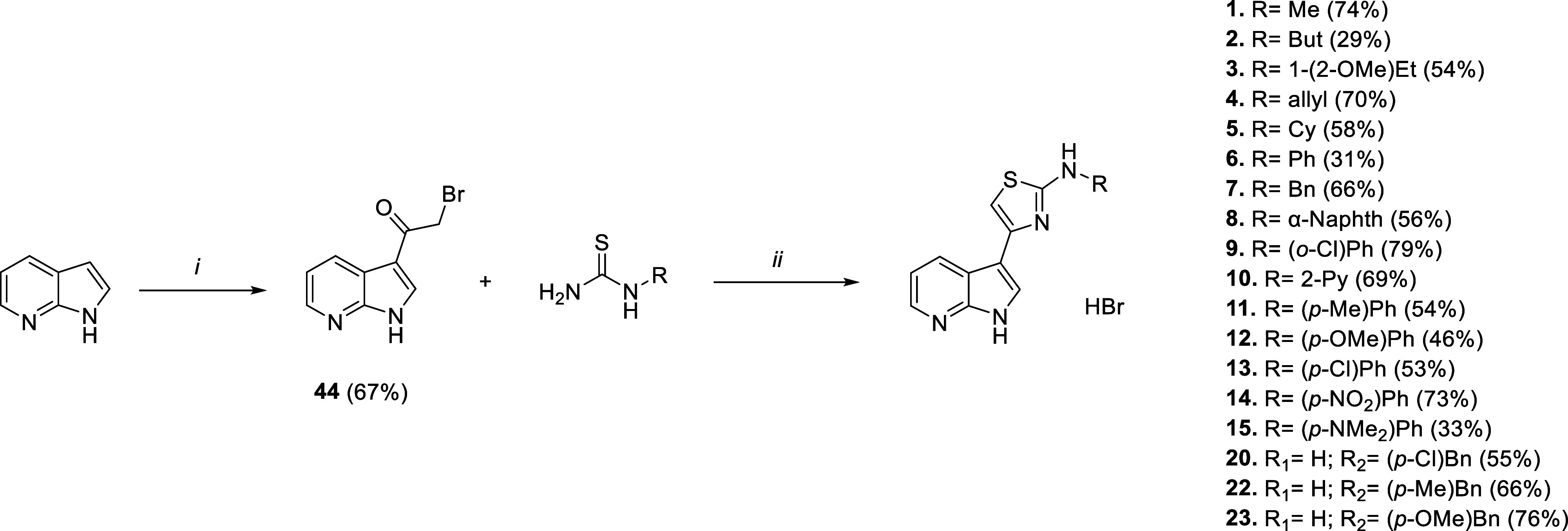
Synthetic Routes for Compounds **1**−**15**, **20**, **22** and **23** ^a^

a Reagents and Conditions: (i) BrAcBr, AlCl_3_, DCM, Reflux, 2 h; (ii) EtOH, Reflux, 1 h.

**Scheme 2:**
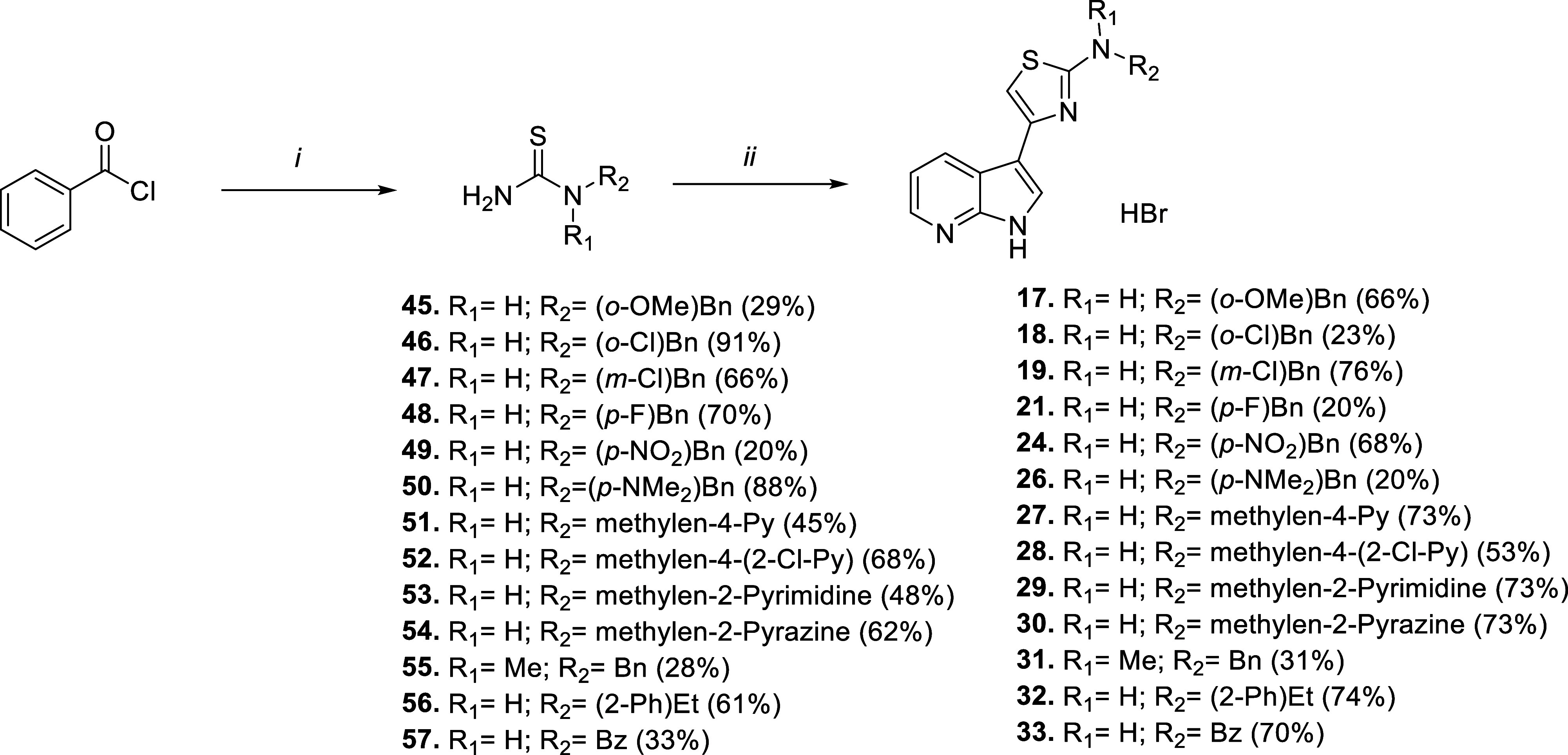
Synthetic Routes for Compounds **17**−**19**, **21**, **24** and **26−33** ^a^

a Reagents and Conditions: (i) a. NH_4_SCN, Acetone, MW, 15 min, 60 °C; b. NHR_1_R_2_, MW, 15 min, 60 °C; c. K_2_CO_3_, H_2_O/EtOH, Reflux, 2 h (for **49** c. HCl, Reflux, 2h; no Step c for **57**); (ii) **44**, EtOH, Reflux, 1 h.

**Scheme 3:**
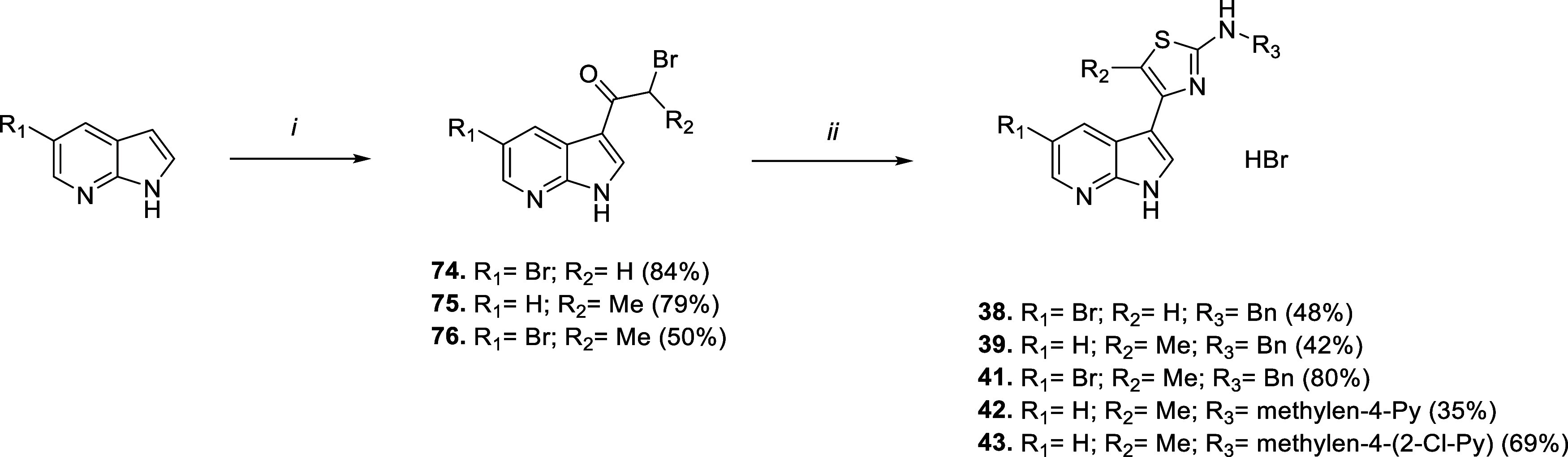
Synthetic Routes for Compounds **38**, **39** and **41−43** ^a^

a Reagents and Conditions: (i) BrAcBr or 2-BrPrBr, AlCl_3_, DCM, Reflux, 2 h; (ii) R_3_-thiourea, EtOH, Reflux, 1 h.

**Scheme 4:**
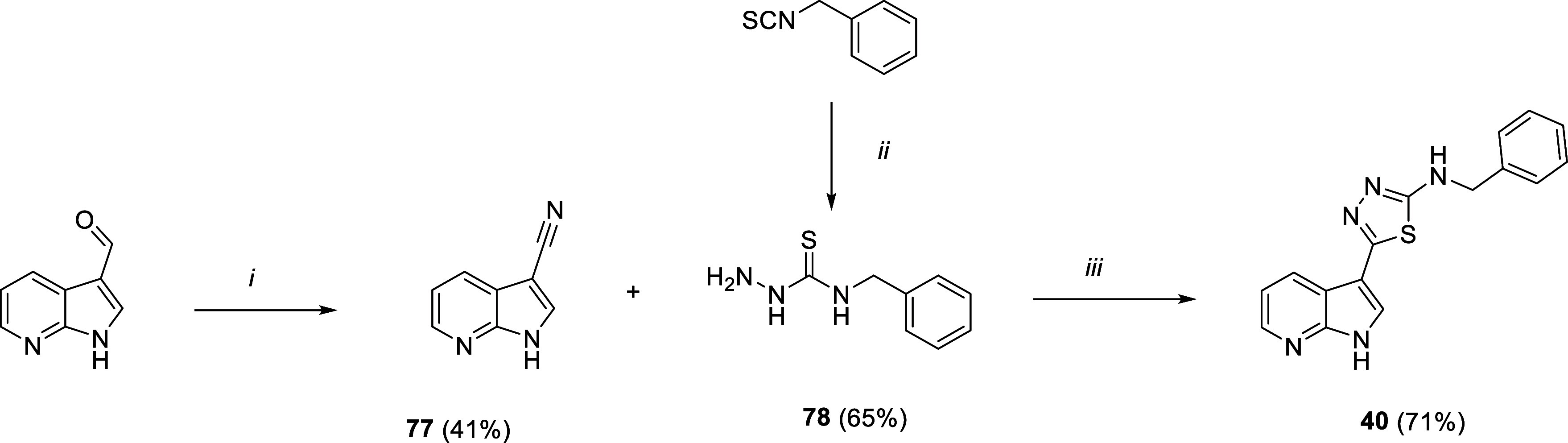
Synthetic Route for Compound **40** ^a^

a Reagents and Conditions: (i) NH_2_OH, HCOOH, HCOONH_4_, Reflux, 2 h; (ii) (NH_2_)_2_, DCM, rt, 2 h; (iii) TFA, Reflux, 2 h.

## Conclusions

The chance to develop late-stage treatments which could promote the recovery of neural trophism by favoring the switch from neurodegenerative to neuroregenerative processes paired with neuroprotective activities emerged as a promising paradigm for CNS-directed drug discovery programs. Pursuing this approach, we identified protein kinases Fyn and GSK-3β as valid targets in this respect due to their multifaceted involvement in both activation and fostering of neurotoxic pathways besides the modulation of neurogenic/neurodevelopmental processes. Thus, in search of Fyn/GSK-3β inhibitors, we have herein sought to develop new dual kinase inhibitors starting from the pharmacophore of the 7-azaindole-3-aminothiazole, endowed with a mild and unbalanced Fyn/GSK-3β kinase inhibitor profile. After deepened SAR studies, we achieved two selective inhibitors, one Fyn-referred and one for GSK-3β (**41** and **40**, respectively), and two dual nanomolar inhibitors (compounds **28** and **43**) that, together with an inactive compound of the series, enabled us to investigate the role of the two kinases regarding neuromodulatory and neuroprotective properties. An initial evaluation in a primary neuron cell culture highlighted no toxicity issues paired with an unusual increase in the registered cell viability, which was amplified in the more potent Fyn inhibitors, stimulating the following biological investigations to define the underlying mechanism. First, the ability to mitigate the physiological senescence and mortality rate was noticed only for Fyn-selective (compound **41**) and dual inhibitors (compounds **28** and **43**), reaffirming an important role for Fyn inhibition in tackling neuronal aging. Second, tested inhibitors proved to trigger both proliferation and neuromodulation of neural progenitor cells, with slightly different behavior depending on their kinase inhibition profile: marked neurogenesis after treatment with GSK-3β, more potent inhibitors **40** and **28** and pronounced glial differentiation with Fyn, more potent inhibitors **41** and **43**. Finally, the selected inhibitors generally tuned inflammatory response by reducing microglial pro-inflammatory activation without repressing its anti-inflammatory reactivity, emerging with remarkable immunomodulatory activities for compounds **28** and **41**.

Although a promising selectivity emerged for **41** and **43**, further improvements in this regard are needed to fully depict kinases involvement. Ultimately, the resulting dual inhibitors **28** and **43**, albeit still not perfectly balanced, represent valuable pharmacological tools due to their polyhedral neuroprotective and neuromodulatory profiles combined with proven *in vitro* BBB-permeability. Furthermore, extensive evaluation under pathological conditions becomes necessary and will be carried out to further verify their therapeutic properties and plausibly lay the ground for the future development of potential dual neuroprotective-neuroregenerative agents.

## Material and Methods

### Chemistry

Chemical reagents were purchased from Merck, TCI, and Fluorochem. Nuclear magnetic resonance spectra (NMR) were recorded at 400 MHz for ^1^H and 100 MHz for ^13^C on a Varian VXR 400 spectrometer or Bruker Avance III 400 system equipped with a BBI probe and Z-gradients in CDCl_3_, DMSO-*d*_6_, or CD_3_OD as solvents. Chemical shifts (d) are given in ppm from tetramethylsilane (TMS) with the solvent resonance as the internal standard (CDCl_3_: δ 7.26, DMSO-*d*_6_: δ 2.50, CD_3_OD: δ 3.31 for ^1^H NMR and CDCl_3_: δ 77.16, DMSO-*d*_6_: δ 39.52, CD_3_OD: δ 49.00 for ^13^C NMR). For ^1^H NMR, data are reported as follows: chemical shift, multiplicity (s = singlet, d = doublet, dd = double of doublets, t = triplet, q = quartet, m = multiplet, p = pentet, dt = doublet of triplets, td = triplet of doublets, tt = triplet of triplets, qd = quartet of doublets, br s = broad singlet), coupling constants (Hz), and integration. Microwave-assisted synthesis was performed by using CEM Discover Standard Precision (SP) apparatus (2.45 GHz, maximum power of 300 W). Melting points were measured in glass capillary tubes on a Stuart SMP-10 apparatus and are uncorrected. Chromatographic separations were performed on silica gel columns through flash or gravity column (Kieselgel 40, 0.040–0.063 mm; Merck) chromatography. Reactions were followed by thin-layer chromatography (TLC) on Merck (0.25 mm) glass-packed precoated silica gel plates (60 F254) that were visualized in an iodine chamber or with a UV lamp, KMnO_4_, or bromocresol green. All names were attributed by Chem BioDraw Ultra 22.2.0. Final compounds mass spectra were recorded on a Waters ACQUITY ARC UHPLC/MS system. All final compounds were pure >95% as determined via UHPLC/MS analyses run on a Waters ACQUITY ARC UHPLC/MS system, consisting of a QDa mass spectrometer equipped with an electrospray ionization interface and a 2489 UV/vis detector. The detected wavelengths (λ) were 254 and 365 nm. The analyses were performed on an XBridge BEH C18 column (10 mm × 2.1 mm i.d., particle size 2.5 μm) with an XBridge BEH C18 VanGuard Cartridge precolumn (5 mm × 2.1 mm i.d., particle size 1.8 μm). The mobile phases were H_2_O (0.1% formic acid) (A) and MeCN (0.1% formic acid) (B). Electrospray ionization in positive and negative modes was applied in the mass scan range of 50–1200 Da. Method and gradients used were the following: hydrophilic gradient: 0–0.5 min, 5% B; 0.5–1.5 min, 25% B; 1.5–2 min, 25% B; 2–3.5 min, 70% B; 3.5–3.9 min, 70% B; 3.90–4 min, 5% B; 4–5.73 min, 5% B; generic gradient: 0–0.78 min, 20% B; 0.78–2.87 min, 20–95% B; 2.87–3.54 min, 95% B; 3.54–3.65 min, 95–20% B; 3.65–5.73, 20% B. Flow rate: 0.8 mL/min. Generic gradient was used for all compounds apart from compounds **10**, **16**, **25**, **27**–**30**, **40**, and **42** that required hydrophilic gradient.

#### General Procedure A: Friedel-Craft Acylation

To a solution of 7-azaindole, or analogues, (1 equiv) in anhydrous DCM (25 mL), AlCl_3_ (3 equiv) was slowly added and the reaction mixture was left stirring for 15 min. Then, the respective acyl bromide (1.1 equiv) was added dropwise and the reaction was monitored by TLC. After 1 h, the reaction was allowed to cool down to room temperature and quenched with water (25 mL). The obtained mixture was extracted, the organic layer dried over sodium sulfate, and the resulting crude purified by column chromatography to obtain the pure product.

#### General Procedure B: Thiourea Preparation

A mixture of benzoyl chloride (1 equiv) and ammonium thiocyanate (1.2 equiv) was stirred in acetone (1–3 mL) under microwave irradiation (60 °C, 250 psi, 80 W) for 15 min. After this time, the opportune substituted benzylamine or aniline (1 equiv) was added and the mixture was irradiated for additional 15 min under the same condition. Then, the reaction mixture was filtered, the precipitate was washed with acetone, and the filtrate concentrated under vacuo. The resulting crude was dissolved in the minimal amount of EtOH (1–2 mL) and a solution of K_2_CO_3_ 2 M (2 equiv) was added dropwise at room temperature. To obtain intermediate **49**, the resulting crude product was directly suspended in HCl 37% (7 mL). The reaction was stirred at reflux for 2h and it was monitored by TLC. After this period, the reaction mixture was cooled down to room temperature and water (10–20 mL) was added dropwise (to obtain intermediate **49** the mixture was previously basified with 30% NH_4_OH_(aq)_ until pH 8). After extraction with DCM (4 × 20 mL), the organic phases were reunited, dried with sodium sulfate, filtered, and concentrated under vacuo. The obtained crude product was purified with column chromatography to give the pure products.

#### General Procedure C: Hantzsch Cyclization

A mixture of bromoacetyl-intermediate (1 equiv) and the opportune thiourea (1 equiv) in EtOH or acetonitrile (2–4 mL) was stirred at reflux for 1 h and the reaction was monitored by TLC. Then, the reaction mixture was cooled to room temperature and then put in an ice and salt bath for 20 min. A colored solid separated out that was filtered and washed using cold EtOH and then dried under vacuum to obtain the pure product.

##### 2-Bromo-1-(1*H*-pyrrolo­[2,3-*b*]­pyridin-3-yl)­ethan-1-one (**44**)

Compound **44** was obtained following general procedure A using 7-azaindole (1.0 g, 8.46 mmol) and bromoacetyl bromide. Compound was eluted with DCM/MeOH/30% NH_4_OH_(aq)_ (9.6/0.4/0.05) which afforded **44** as an off-white solid (1.35 g, 67%). ^1^H NMR (DMSO-*d*_6_, 400 MHz): δ 12.67 (br s, 1H), 8.63 (s, 1H), 8.45 (d, *J* = 8 Hz, 1H), 8.35 (d, *J* = 8 Hz, 1H), 7.29–7.27 (m, 1H), 4.69 (s, 2H). ^13^C NMR (DMSO- *d*_6_, 100 MHz): δ 187.08, 144.70, 136.15, 134.38, 130.38, 118.87, 118.54, 112.72, 33.67.

##### 2-Bromo-1-(5-bromo-1*H*-pyrrolo­[2,3-*b*]­pyridin-3-yl)­ethan-1-one (**74**)

Compound **74** was obtained following general procedure A using 5-bromo-7-azaindole (500 mg, 2.54 mmol) and bromoacetyl bromide. Compound was eluted with DCM/MeOH/30% NH_4_OH_(aq)_ (9.5/0.5/0.05) which afforded **64** as a light-brown solid (680 mg, 84%). ^1^H NMR (DMSO-*d*_6_, 400 MHz): δ 12.90 (br s, 1H), 8.68 (s, 1H), 8.54 (s, 1H), 8.42 (s, 1H), 4.70 (s, 2H). ^13^C NMR (DMSO-*d*_6_, 100 MHz): δ 187.03, 147.89, 145.19, 137.34, 131.68, 119.87, 114.23, 112.16, 33.56.

##### 2-Bromo-1-(1*H*-pyrrolo­[2,3-*b*]­pyridin-3-yl)­propan-1-one (**75**)

Compound **65** was obtained following general procedure A using 7-azaindole (250 mg, 2.12 mmol) and 2-bromopropionyl bromide. Compound was eluted with DCM/MeOH/30% NH_4_OH_(aq)_ (9.8/0.2/0.05) which afforded **65** as a yellow solid (450 mg, 79%). ^1^H NMR (DMSO-*d*_6_, 400 MHz): δ 12.68 (br s, 1H), 8.67 (s, 1H), 8.47 (d, *J* = 7 Hz, 1H), 8.35 (d, *J* = 7 Hz, 1H), 7.30–7.27 (m, 1H), 5.67 (q, *J* = 6.8 Hz, 1H), 1.78 (d, *J* = 6.8 Hz, 3H). ^13^C NMR (DMSO-*d*_6_, 100 MHz): δ 188.82, 149.03, 144.46, 135.05, 129.53, 118.25, 117.95, 111.41, 44.48, 20.42.

##### 2-Bromo-1-(5-bromo-1*H*-pyrrolo­[2,3-*b*]­pyridin-3-yl)­propan-1-one (**76**)

Compound **66** was obtained following general procedure A using 5-bromo-7-azaindole (300 mg, 1.52 mmol) and 2-bromopropionyl bromide. Compound was eluted with DCM/MeOH/30% NH_4_OH_(aq)_ (9.8/0.2/0.05) which afforded **66** as a light-brown solid (250 mg, 50%). ^1^H NMR (DMSO-*d*_6_, 400 MHz): δ 12.89 (br s, 1H), 8.70 (d, *J* = 4 Hz, 1H), 8.55 (d, *J* = 4 Hz, 1H), 8.41–8.40 (m, 1H), 5.63 (q, *J* = 8 Hz, 1H), 1.74 (d, *J* = 8 Hz, 3H). ^13^C NMR (DMSO-*d*_6_, 100 MHz): δ 189.42, 148.01, 145.22, 136.99, 131.81, 120.22, 114.21, 111.52, 44.88, 20.84.

##### 1-(2-Methoxybenzyl)­thiourea (**45**)

Compound **45** was obtained following general procedure B using (2-methoxyphenyl)­methanamine (0.46 mL, 3.56 mmol). Compound was eluted with DCM/MeOH/30% NH_4_OH_(aq)_ (9.5/0.5/0.05) which afforded **45** as a pale-yellow solid (200 mg, 29%). ^1^H NMR (DMSO-*d*_6_, 400 MHz): δ 7.76 (br s, 1H), 7.27–7.22 (m, 1H), 7.19 (d, *J* = 7 Hz, 1H), 7.05 (br s, 2H), 6.98 (d, *J* = 7 Hz, 1H), 6.91 (t, *J* = 7 Hz, 1H), 4.55 (s, 2H), 3.80 (s, 3H). ^13^C NMR (DMSO-*d*_6_, 100 MHz): δ 183.87, 157.18, 133.11, 128.70, 126.99, 120.53, 110.92, 55.75, 43.27.

##### 1-(2-Chlorobenzyl)­thiourea (**46**)

Compound **46** was obtained following general procedure B using (2-chlorophenyl)­methanamine (0.17 mL, 1.42 mmol). Compound was eluted with DCM/MeOH/30% NH_4_OH_(aq)_ (9.7/0.3/0.05) which afforded **46** as a pale-yellow solid (260 mg, 91%). ^1^H NMR (DMSO-*d*_6_, 400 MHz): δ 7.96 (br s, 1H), 7.42 (d, *J* = 8 Hz, 1H), 7.32–7.26 (m, 3H), 7.18 (br s, 2H), 4.68 (s, 2H). ^13^C NMR (DMSO-*d*_6_, 100 MHz): δ 184.23, 136.88. 132.38, 129.52, 129.41, 129.05, 127.52, 45.61.

##### 1-(3-Chlorobenzyl)­thiourea (**47**)

Compound **47** was obtained following general procedure B using (3-chlorophenyl)­methanamine (0.46 mL, 3.56 mmol). Compound was eluted with DCM/MeOH/30% NH_4_OH_(aq)_ (9.6/0.4/0.05) which afforded **47** as a pale-yellow solid (470 mg, 66%). ^1^H NMR (DMSO-*d*_6_, 400 MHz): δ 8.04 (br s, 1H), 7.38–7.29 (m, 3H), 7.24 (d, *J* = 7.6 Hz, 1H) 7.16 (br s, 2H), 4.65 (s, 2H). ^13^C NMR (DMSO-*d*_6_, 100 MHz): δ 184.09, 142.59, 133.34, 130.57, 127.37, 127.17, 126.34, 47.09.

##### 1-(4-Fluorobenzyl)­thiourea (**48**)

Compound **48** was obtained following general procedure B using (4-fluorophenyl)­methanamine (0.12 mL, 1.01 mmol). Compound was eluted with DCM/MeOH/30% NH_4_OH_(aq)_ (9.5/0.5/0.05) which afforded **48** as a pale-yellow solid (130 mg, 70%). ^1^H NMR (DMSO-*d*_6_, 400 MHz): δ 8.04 (br s, 1H), 7.34–7.31 (m, 2H), 7.17–7.12 (m, 4H), 4.60 (s, 2H). ^13^C NMR (DMSO-*d*_6_, 100 MHz): δ 183.90, 162.85, 129.71, 115.51 (2C) 115.29 (2C), 47.05.

##### 1-(4-Nitrobenzyl)­thiourea (**49**)

Compound **49** was obtained following general procedure B using (4-nitrobenzyl)­methanamine (671 mg, 3.56 mmol). Compound was eluted with DCM/MeOH/30% NH_4_OH_(aq)_ (9.5/0.5/0.05) which afforded **49** as a pale-yellow solid (150 mg, 20%). ^1^H NMR (DMSO-*d*_6_, 400 MHz): δ 8.17 (d, *J* = 8 Hz, 2H), 8.12 (br s, 1H), 7.49 (d, *J* = 8 Hz, 2H), 7.22 (br s, 2H), 4.75 (s, 2H). ^13^C NMR (DMSO-*d*_6_, 100 MHz): δ 182.50, 146.82, 128.60, 128.48 (2C), 127.85, 123.84 (2C), 47.06.

##### 1-(4-(Dimethylamino)­benzyl)­thiourea (**50**)

Compound **50** was obtained following general procedure B using 4-(aminomethyl)-*N*,*N*-dimethylaniline (0.34 mL, 2.33 mmol). Compound was eluted with DCM/MeOH/30% NH_4_OH_(aq)_ (9.8/0.2/0.05) which afforded **50** as a pale-yellow solid (283 mg, 88%). ^1^H NMR (DMSO-*d*_6_, 400 MHz): δ 7.74 (br s, 1H), 7.09 (d, *J* = 8 Hz, 2H), 6.91 (br s, 2H), 6.65 (d, *J* = 8 Hz, 2H), 4.42 (s, 2H), 2.82 (s, 6H). ^13^C NMR (DMSO-*d*_6_, 100 MHz): δ 183.43, 150.17, 128.93 (2C), 126.81, 112.78 (2C), 47.71, 40.71 (2C).

##### 1-(Pyridin-4-ylmethyl)­thiourea (**51**)

Compound **51** was obtained following general procedure B using 4-pyridylmethaneamine (0.46 mL, 3.56 mmol). Compound was eluted with DCM/MeOH/30% NH_4_OH_(aq)_ (8.7/1.3/0.1) which afforded **51** as a pale-yellow solid (260 mg, 45%). ^1^H NMR (DMSO-*d*_6_, 400 MHz): δ 8.49 (d, *J* = 5.6 Hz, 2H), 7.24 (d, *J* = 5.6 Hz, 2H), 4.67 (s, 2H). ^13^C NMR (DMSO-*d*_6_, 100 MHz): δ 182.45, 128.83 (2C), 127.58, 127.50 (2C), 56.31.

##### 1-((2-Chloropyridin-4-yl)­methyl)­thiourea (**52**)

Compound **52** was obtained following general procedure B using compound **58** (310 mg, 2.17 mmol). Compound was eluted with DCM/MeOH/30% NH_4_OH_(aq)_ (9.5/0.5/0.05) which afforded **52** as a yellowish solid (298 mg, 68%). ^1^H NMR (DMSO-*d*_6_, 400 MHz): δ 8.31 (d, *J* = 4.0 Hz, 1H), 8.07 (br s, 1H), 7.29–7.23 (m, 4H) 4.65 (s, 2H). ^13^C NMR (DMSO-*d*_6_, 100 MHz): δ 184.45, 153.67, 150.69, 150.13, 122.51, 121.93, 46.31.

##### 1-(Pyrimidin-2-ylmethyl)­thiourea (**53**)

Compound **53** was obtained following general procedure B using pyrimidin-2-ylmethanamine (388 mg, 3.56 mmol). Compound was eluted with DCM/MeOH/30% NH_4_OH_(aq)_ (9.5/0.5/0.05) which afforded **53** as a pale-yellow solid (280 mg, 48%). ^1^H NMR (DMSO-*d*_6_, 400 MHz): δ 8.75 (d, *J* = 7.0 Hz, 2H), 8.01 (br s, 1H), 7.38 (t, *J* = 7.0 Hz, 1H), 7.26 (br s, 2H), 4.79 (s, 2H). ^13^C NMR (DMSO-*d*_6_, 100 MHz): δ 183.91, 166.77, 157.74 (2C), 120.29, 50.32.

##### 1-(Pyrazin-2-ylmethyl)­thiourea (**54**)

Compound **54** was obtained following general procedure B using pyrazin-2-ylmethanamine (388 mg, 3.56 mmol). Compound was eluted with DCM/MeOH/30% NH_4_OH_(aq)_ (9.5/0.5/0.05) which afforded **54** as a pale-yellow solid (370 mg, 62%). ^1^H NMR (DMSO-*d*_6_, 400 MHz): δ 8.56–8.51 (m, 3H), 8.13 (br s, 1H), 7.27 (br s, 2H), 4.75 (s, 2H). ^13^C NMR (DMSO-*d*_6_, 100 MHz): δ 184.25, 154.48, 144.26, 143.83, 143.49, 47.52.

##### 1-Benzyl-1-methylthiourea (**55**)

Compound **55** was obtained following general procedure B using *N*-methylbenzylamine (0.46 mL, 3.56 mmol). Compound was eluted with DCM/MeOH/30% NH_4_OH_(aq)_ (9.8/0.2/0.05) which afforded **55** as a pale-yellow solid (180 mg, 28%). ^1^H NMR (DMSO-*d*_6_, 400 MHz): δ 7.39 (br s, 2H) 7.36–7.32 (m, 2H), 7.27–7.24 (m, 3H), 4.99 (s, 2H), 3.33 (s, 3H). ^13^C NMR (DMSO-*d*_6_, 100 MHz): δ 184.45, 150.74, 150.00, 149.85, 122.57, 122.46, 121.69, 46.61, 42.22.

##### 1-Phenethylthiourea (**56**)

Compound **56** was obtained following general procedure B using 2-phenylethan-1-amine (0.46 mL, 3.56 mmol). Compound was eluted with DCM/MeOH/30% NH_4_OH_(aq)_ (9.7/0.3/0.05) which afforded **56** as a white solid (390 mg, 61%). ^1^H NMR (DMSO-*d*_6_, 400 MHz): δ 7.53 (br s, 1H), 7.30–7.26 (m, 2H), 7.22–7.17 (m, 3H), 6.94 (br s, 2H), 3.57–3.56 (m, 2H), 2.76 (t, *J* = 6.0 Hz, 2H). ^13^C NMR (DMSO-*d*_6_, 100 MHz): δ 183.59, 139.78, 129.08 (2C), 128.75 (2C), 126.53, 45.70, 35.31.

##### *N*-Carbamothioylbenzamide (**57**)

A mixture of benzoyl chloride (0.41 mL, 3.56 mmol) and ammonium thiocyanate (298 mg, 3.92 mmol) was suspended in acetone (2 mL) and stirred under microwave irradiation (60 °C, 80 W, 250 psi) for 15 min. The mixture was then filtered, the precipitate was washed with acetone, and the filtrate concentrated under vacuo. The resulting crude product was further suspended in an aqueous ammonia 30% solution (1.78 mL) with acetone (2 mL) and stirred at room temperature for 30 min. After completion, the reaction mixture was then placed at 0 °C and cold ethanol was added dropwise. The precipitate formed was filtered, washed with cold EtOH, and dried under vacuum to obtain the pure product as an off-white solid (210 mg, 33%). ^1^H NMR (DMSO-*d*_6_, 400 MHz): δ 11.20 (br s, 1H), 9.82 (br s, 1H), 9.53 (br s, 1H), 7.90–7.88 (m, 2H), 7.62–7.58 (m, 1H), 7.47 (t, *J* = 8.0 Hz, 2H). ^13^C NMR (DMSO-*d*_6_, 100 MHz): δ 182.49, 168.20, 133.39, 132.68, 128.99, 128.81.

##### 1*H*-Pyrrolo­[2,3-*b*]­pyridine-3-carbonitrile (**77**)

To a solution of 7-azaindole-3-carbaldehyde (250 mg, 1.71 mmol) in formic acid (1.7 mL), hydroxylamine hydrochloride (170 mg, 2.57 mmol), and ammonium formate (220 mg, 3.42 mmol) were added, and the reaction was left stirring at reflux for 2 h, monitored by TLC. After completion, the reaction was cooled down to room temperature, and water (5 mL) was added dropwise. The obtained precipitate was filtered, washed with water, and dried under vacuum to obtain the pure product as a white powder (100 mg, 41%). ^1^H NMR (DMSO-*d*_6_, 400 MHz): δ 12.83 (br s, 1H), 8.44 (s 1H), 8.42 (d, *J* = 4.8 Hz, 1H), 8.12 (d, *J* = 8.4 Hz, 1H), 7.31–7.28 (m, 1H). ^13^C NMR (DMSO-*d*_6_, 100 MHz): δ 147.38, 145.02, 135.51, 127.31, 118.87, 117.86, 115.56, 83.24.

##### *N*-Benzylhydrazinecarbothioamide (**78**)

Benzylisothiocyanate (250 mg, 1.68 mmol) and hydrazine hydrate (250 mg, 5.03 mmol) were suspended in DCM (1.5 mL), and the reaction was stirred for 2 h at room temperature and monitored by TLC. The resulting precipitate was filtered, washed with DCM, and dried in vacuo to obtain the pure compound as a yellow solid (200 mg, 65%). ^1^H NMR (DMSO-*d*_6_, 400 MHz): δ 8.70 (br s,1H), 8.25 (br s, 1H), 7.29–7.24 (m, 4H), 7.20–7.17 (m, 1H), 4.68 (d, *J* = 4.0 Hz, 1H), 4.47 (br s, 2H). ^13^C NMR (DMSO-*d*_6_, 100 MHz): δ 182.01, 140.23, 128.51 (2C), 127.75 (2C), 127.07, 46.57.

##### *N*-Methyl-4-(1*H*-pyrrolo­[2,3-*b*]­pyridin-3-yl)­thiazol-2-amine Hydrobromide (**1**)

Compound **1** was obtained following general procedure C using **44** (150 mg, 0.63 mmol) and 1-methyl thiourea (57 mg, 0.63 mmol), affording it as a yellowish solid (145 mg, 74%). ^1^H NMR (DMSO-*d*_6_, 400 MHz): δ 12.29 (br s, 1H), 9.31 (br s, 1H), 8.38–8.36 (m, 2H), 8.11 (s, 1H), 7.28 (dd, ^1^ *J* = 4.8 Hz, ^2^ *J* = 5.2 Hz, 1H), 7.10 (s, 1H), 3.07 (s, 3H). ^13^C NMR (DMSO-*d*_6_, 100 MHz): δ 169.48, 147.28, 142.73, 136.01, 129.03, 126.26, 117.35, 116.57, 105.04, 98.99, 31.99. MS [ESI+] *m*/*z*: 231.11 [M + H]^+^.

##### *N*-Butyl-4-(1*H*-pyrrolo­[2,3-*b*]­pyridin-3-yl)­thiazol-2-amine Hydrobromide (**2**)

Compound **2** was obtained following general procedure C using **44** (100 mg, 0.42 mmol) and 1-butylthiourea (55 mg, 0.42 mmol), affording it as a yellowish solid (44 mg, 29%). ^1^H NMR (DMSO-*d*_6_, 400 MHz): δ 12.26 (br s, 1H), 9.29 (br s, 1H), 8.36 (d, *J* = 4.8 Hz, 2H), 8.06 (d, *J* = 6.0 Hz, 1H), 7.29–7.26 (m, 1H), 7.06 (d, *J* = 4.0 Hz, 1H), 3.45–3.44 (m, 2H), 1.65–1.59 (m, 2H), 1.43–1.37 (m, 2H), 0.93 (t, *J* = 7.2 Hz, 3H). ^13^C NMR (DMSO-*d*_6_, 100 MHz): δ 168.40, 147.12, 142.53, 128.91, 126.04, 117.28, 116.38, 116.06, 105.18, 98.75, 45.38, 29.96, 19.33, 13.46. MS [ESI+]: 273.12 [M + H]^+^.

##### *N*-(2-Methoxyethyl)-4-(1*H*-pyrrolo­[2,3-*b*]­pyridin-3-yl)­thiazol-2-amine Hydrobromide (**3**)

Compound **3** was obtained following general procedure C using **44** (150 mg, 0.63 mmol) and 1-(4-methoxyethyl) thiourea (85 mg, 0.63 mmol), affording it as an orange solid (121 mg, 54%). ^1^H NMR (DMSO-*d*_6_, 400 MHz): δ 12.31 (br s, 1H), 9.30 (br s, 1H), 8.40 (d, *J* = 7.6 Hz, 2H), 8.38 (d, *J* = 4.0 Hz, 2H) 8.08 (s, 1H), 7.31–7.27 (m, 1H), 7.06 (s, 1H), 3.65 (d, *J* = 4.2 Hz, 2H), 3.58 (t, *J* = 4.2 Hz, 2H), 3.32 (s, 3H). ^13^C NMR (DMSO-*d*_6_, 100 MHz): δ 168.76, 146.85, 142.28, 129.50, 126.25, 126.21, 117.67, 116.50, 105.52, 99.20, 69.80, 58.17, 45.40. MS [ESI+]: 275.12 [M + H]^+^.

##### *N*-Allyl-4-(1*H*-pyrrolo­[2,3-*b*]­pyridin-3-yl)­thiazol-2-amine Hydrobromide (**4**)

Compound **4** was obtained following general procedure C using **44** (150 mg, 0.63 mmol) and 1-allyl thiourea (73 mg, 0.63 mmol), affording it as a yellow solid (149 mg, 70%). ^1^H NMR (DMSO-*d*_6_, 400 MHz): δ 12.35 (br s, 1H), 9.31 (br s, 1H), 8.43 (d, *J* = 8.0 Hz, 1H), 8.37 (d, *J* = 4.6 Hz, 1H), 8.08 (s, 1H), 7.30 (dd, ^1^ *J* = 4.6 Hz, ^2^ *J* = 8.0 Hz, 1H), 7.09 (s, 1H), 5.98–5.88 (m, 1H), 5.34 (d, *J* = 17.2 Hz, 1H), 5.24 (d, *J* = 10.8 Hz, 1H), 4.10 (d, *J* = 5.2 Hz, 2H). ^13^C NMR (DMSO-*d*_6_, 100 MHz): δ 168.83, 146.55, 142.02, 136.99, 133.05, 130.12, 126.42, 118.06, 117.50, 116.65, 106.09, 99.56, 47.80. MS [ESI+] *m*/*z*: 257.15 [M + H]^+^.

##### *N*-Cyclohexyl-4-(1*H*-pyrrolo­[2,3-*b*]­pyridin-3-yl)­thiazol-2-amine Hydrobromide (**5**)

Compound **5** was obtained following general procedure C using **44** (150 mg, 0.63 mmol) and 1-cyclohexyl thiourea (100 mg, 0.63 mmol), affording it as a pale-yellow solid (138 mg, 58%). ^1^H NMR (DMSO-*d*_6_, 400 MHz): δ 12.29 (br s, 1H), 9.44 (br s, 1H), 8.35–8.33 (m, 2H), 8.08 (s, 1H), 7.29–7.26 (m, 1H), 7.04 (s, 1H), 3.78–3.76 (m, 1H), 1.99–1.95 (m, 2H), 1.76–1.69 (m, 2H), 1.60–1.57 (m, 1H), 1.38–133 (m, 4H), 1.24–1.18 (m, 1H). ^13^C NMR (DMSO-*d*_6_, 100 MHz): δ 167.75, 147.57, 143.04, 135.90, 129.46, 126.80, 117.93, 116.95, 105.36, 99.57, 55.08, 32.12, 25.25, 24.49. MS [ESI+] *m*/*z*: 299.23 [M + H]^+^.

##### *N*-Phenyl-4-(1*H*-pyrrolo­[2,3-*b*]­pyridin-3-yl)­thiazol-2-amine (**6**)

Compound **6** was obtained following general procedure C using **44** (150 mg, 0.63 mmol) and 1-phenyl thiourea (96 mg, 0.63 mmol), affording it as a yellow solid (73 mg, 31%). ^1^H NMR (DMSO-*d*_6_, 400 MHz): δ 12.49 (br s, 1H), 10.32 (br s, 1H), 8.82 (d, *J* = 7.6 Hz, 1H), 8.44 (d, *J* = 5.2 Hz, 1H), 8.09 (s, 1H), 7.73 (d, *J* = 8.0 Hz, 2H), 7.44 (dd, *J* ^1^ = 5.2 Hz, *J* ^2^ = 7.8 Hz, 1H), 7.36 (t, *J* = 8.0 Hz, 2H), 7.22 (s, 1H), 6.98 (t, *J* = 7.2 Hz, 1H). ^13^C NMR (DMSO-*d*_6_, 100 MHz): δ 163.14, 144,43, 141.23, 139.02, 132.59, 129.18, 129.09 (2C), 125.88, 121.93, 121.34, 119.65, 117.35, 117.05 (2C), 116.11, 111.33, 100.58. MS [ESI+] *m*/*z*: 293.13 [M + H]^+^.

##### *N*-Benzyl-4-(1*H*-pyrrolo­[2,3-*b*]­pyridin-3-yl)­thiazol-2-amine Hydrobromide (**7**)

Compound **7** was obtained following general procedure C using **44** (150 mg, 0.63 mmol) and 1-benzyl thiourea (105 mg, 0.63 mmol), affording it as a pale-yellow solid (124 mg, 51%). ^1^H NMR (DMSO-*d*_6_, 400 MHz): δ 12.37 (br s, 1H), 9.48 (br s, 1H), 8.44 (d, *J* = 8.0 Hz, 1H), 8.38 (d, *J* = 4.8 Hz, 1H), 8.11 (s, 1H), 7.45–7.37 (m, 4H), 7.33–7.28 (m, 2H), 7.07 (s, 1H), 4.68 (s, 2H). ^13^C NMR (DMSO-*d*_6_, 100 MHz): δ 168.67, 146.07, 141.41, 137.07, 130.39, 128.61 (2C), 128.48, 127.75 (2C), 127.63, 127.54, 126.21, 118.16, 116.40, 99.31, 48.78. MS [ESI+] *m*/*z*: 307.13 [M + H]^+^.

##### *N*-(Naphthalen-1-yl)-4-(1*H*-pyrrolo­[2,3-*b*]­pyridin-3-yl)­thiazol-2-amine Hydrobromide (**8**)

Compound **8** was obtained following general procedure C using **44** (150 mg, 0.63 mmol) and 1-naphthyl thiourea (127 mg, 0.63 mmol), affording it as a pinkish solid (150 mg, 56%). ^1^H NMR (DMSO-*d*_6_, 400 MHz): δ 12.60 (br s, 1H), 10.41 (br s, 1H), 8.81 (d, *J* = 7.6 Hz, 1H), 8.47 (d, *J* = 5.6 Hz, 1H), 8.32–8.30 (m, 1H), 8.26 (d, *J* = 7.6 Hz, 1H), 8.106 (s, 1H), 7.98–7.96 (m, 1H), 7.73 (d, *J* = 8 Hz, 1H), 7.60–7.57 (m, 3H), 7.46–7.43 (m, 1H), 7.26 (s, 1H). ^13^C NMR (DMSO-*d*_6_, 100 MHz): δ 166.42, 143.25, 143.05, 138.48, 136.77, 134.49, 134.20, 128.76, 126.71, 126.65, 126.57, 126.31, 124.43, 122.68, 120.74, 118.10, 117.90, 116.53, 111.14, 101.95. MS [ESI+] *m*/*z*: 343.14 [M + H]^+^.

##### *N*-(2-Chlorophenyl)-4-(1*H*-pyrrolo­[2,3-*b*]­pyridin-3-yl)­thiazol-2-amine Hydrobromide (**9**)

Compound **9** was obtained following general procedure C using **44** (150 mg, 0.63 mmol) and 1-(2-chlorophenyl)­thiourea (118 mg, 0.63 mmol), affording it as a yellow solid (203 mg, 79%). ^1^H NMR (DMSO-*d*_6_, 400 MHz): δ 12.65 (br s, 1H), 8.84 (d, *J* = 8.0 Hz, 1H), 8.47 (d, *J* = 4.8 Hz, 1H), 8.39 (d, *J* = 8.0 Hz, 1H), 8.10 (s, 1H), 7.51–739 (m, 3H), 7.31 (s, 1H), 7.11–7.07 (m, 1H). ^13^C NMR (DMSO-*d*_6_, 100 MHz): δ 164.26, 138.06, 137.44, 130.27, 130.14, 128.30, 128.19, 126.41, 124.21, 123.29, 122.49, 121.96, 116.60, 116.50, 111.65, 102.73. MS [ESI+] *m*/*z*: 327.04 [M + H]^+^.

##### *N*-(Pyridin-2-yl)-4-(1*H*-pyrrolo­[2,3-*b*]­pyridin-3-yl)­thiazol-2-amine Hydrobromide (**10**)

Compound **10** was obtained following general procedure C using **44** (100 mg, 0.42 mmol) and 2-pyridyl thiourea (50 mg, 0.42 mmol) in anhydrous acetonitrile, affording it as a yellow solid (109 mg, 69%). ^1^H NMR (DMSO-*d*_6_, 400 MHz): δ 12.76 (br s, 1H), 11.89 (br s, 1H), 8.98 (d, *J* = 7.2 Hz, 1H), 8.50 (d, *J* = 5.6 Hz, 1H), 8.39 (d, *J* = 3.6 Hz, 1H), 8.21 (s, 1H), 7.86 (t, *J* = 6.8 Hz, 1H), 7.53–7.49 (m, 1H), 7.47 (s, 1H) 7.25 (d, *J* = 8.4 Hz, 1H), 7.07–7.04 (m, 1H). ^13^C NMR (DMSO-*d*_6_, 100 MHz): δ 160.05, 151.22, 145.02, 142.91, 142.70, 140.01, 137.86, 134.81, 126.94, 121.07, 116.89, 116.48, 112.26, 111.58, 105.11. MS [ESI+] *m*/*z*: 294.22 [M + H]^+^.

##### 4-(1*H*-Pyrrolo­[2,3-*b*]­pyridin-3-yl)-*N*-(*p*-tolyl)­thiazol-2-amine Hydrobromide (**11**)

Compound **11** was obtained following general procedure C using **44** (150 mg, 0.63 mmol) and 1-(4-methylphenyl)-2-thiourea (105 mg, 0.63 mmol), affording it as a pale-yellow solid (131 mg, 54%). ^1^H NMR (DMSO-*d*_6_, 400 MHz): δ 12.76 (br s, 1H), 10.30 (br s, 1H), 8.93 (d, *J* = 8.0 Hz, 1H), 8.49 (d, *J* = 5.6 Hz, 1H), 8.11 (s, 1H), 7.57 (d, *J* = 8.4 Hz, 2H), 7.53–7.49 (m, 1H), 7.22 (s, 1H), 7.13 (d, *J* = 8.4 Hz, 2H), 2.22 (s, 3H). ^13^C NMR (DMSO-*d*_6_, 100 MHz): δ 164.20, 143.54, 141.89, 139.01, 137.19, 135.37, 131.00, 129.92 (2C), 126.99, 121.50, 117.94 (2C), 116.53, 111.95, 101.47, 20.83. MS [ESI+] *m*/*z*: 307.13 [M + H]^+^.

##### *N*-(4-Methoxyphenyl)-4-(1*H*-pyrrolo­[2,3-*b*]­pyridin-3-yl)­thiazol-2-amine Hydrobromide (**12**)

Compound **12** was obtained following general procedure C using **44** (100 mg, 0.42 mmol) and 1-(4-methoxyphenyl)­thiourea (64 mg, 0.42 mmol), affording it as a yellowish solid (77 mg, 46%). ^1^H NMR (DMSO-*d*_6_, 400 MHz): δ 12.51 (br s, 1H), 10.17 (br s, 1H), 8.78 (d, *J* = 7.2 Hz, 1H), 8.42 (d, *J* = 6 Hz, 1H), 8.04 (s, 1H), 7.59 (d, *J* = 9.2 Hz, 2H), 7.44–7.40 (m, 1H), 7.14 (s, 1H), 6.94 (d, *J* = 9.2 Hz, 2H), 3.71 (s, 3H). ^13^C NMR (DMSO-*d*_6_, 100 MHz): δ 164.80, 155.01, 143.63, 138.74, 134.77, 133.75, 126.55, 120.48, 119.91 (2C), 116.56, 114.82 (2C), 111.41, 111.39, 100.62, 55.71. MS [ESI+] *m*/*z*: 323.14 [M + H]^+^.

##### *N*-(4-Chlorophenyl)-4-(1*H*-pyrrolo­[2,3-*b*]­pyridin-3-yl)­thiazol-2-amine Hydrobromide (**13**)

Compound **13** was obtained following general procedure C using **44** (150 mg, 0.63 mmol) and 1-(4-chlorophenyl)­thiourea (118 mg, 0.63 mmol), affording it as a yellowish solid (135 mg, 53%). ^1^H NMR (DMSO-*d*_6_, 400 MHz): δ 12.69 (br s, 1H), 10.48 (br s, 1H), 8.88 (d, *J* = 6.8 Hz, 1H), 8.47 (d, *J* = 5.2 Hz, 1H), 8.12 (s, 1H), 7.76 (d, *J* = 9.0 Hz, 2H), 7.51–7.48 (m, 1H), 7.37 (d, *J* = 9.0 Hz, 2H), 7.27 (s, 1H). ^13^C NMR (DMSO-*d*_6_, 100 MHz): δ 163.23, 144.41, 142.57, 140.53, 137.68, 134.85, 129.29 (2C), 126.94, 124.97, 121.13, 118.88 (2C), 116.59, 112.11, 102.02. MS [ESI+] *m*/*z*: 327.14 [M + H]^+^.

##### *N*-(4-Nitrophenyl)-4-(1*H*-pyrrolo­[2,3-*b*]­pyridin-3-yl)­thiazol-2-amine Hydrobromide (**14**)

Compound **14** was obtained following general procedure C using **44** (100 mg, 0.42 mmol) and 1-(4-nitrophenyl)-2-thiourea (83 mg, 0.42 mmol), affording it as a yellow solid (128 mg, 73%). ^1^H NMR (DMSO-*d*_6_, 400 MHz): δ 12.68 (br s, 1H), 11.17 (br s, 1H), 8.84 (d, *J* = 8.0 Hz, 1H), 8.45 (d, *J* = 5.6 Hz, 1H), 8.24 (d, *J* = 9.2 Hz, 2H), 8.18 (s, 1H), 7.95 (d, *J* = 9.2 Hz, 2H), 7.50–7.47 (m, 1H), 7.42 (s, 1H). ^13^C NMR (DMSO-*d*_6_, 100 MHz): δ 162.07, 147.37, 144.99, 143.32, 140.59, 138.32, 134.12, 127.10, 126.05 (2C), 120.64, 116.70, 116.66 (2C), 111.73, 103.67. MS [ESI+] *m*/*z*: 338.23 [M + H]^+^.

##### *N* ^1^-(4-(1H-Pyrrolo­[2,3-*b*]­pyridin-3-yl)­thiazol-2-yl)-*N*,^4^ *N* ^4^-dimethylbenzene-1,4-diamine Hydrobromide (**15**)

Compound **15** was obtained following general procedure C using **44** (150 mg, 0.63 mmol) and 1-(4-dimethylamino)-thiourea (123 mg, 0.63 mmol), affording it as a dark-green solid (87 mg, 33%). ^1^H NMR (DMSO-*d*_6_, 400 MHz): δ 12.59 (br s, 1H), 10.68 (br s, 1H), 8.81 (d, *J* = 8.0 Hz, 1H), 8.44 (d, *J* = 5.2 Hz, 1H), 8.11 (s, 1H), 7.89 (d, *J* = 9.2 Hz, 2H), 7.73 (d, *J* = 9.2 Hz, 2H), 7.46–7.43 (m, 1H), 7.29 (s, 1H), 3.15 (s, 6H). ^13^C NMR (DMSO-*d*_6_, 100 MHz): δ 162.97, 144.73, 143.97, 142.19, 138.93, 136.46, 133.44, 126.69, 122.09, 120.29, 117.98 (2C), 116.57 (2C), 111.75, 102.06, 46.39 (2C). MS [ESI+] *m*/*z*: 336.19 [M + H]^+^.

##### *N*-(2-Methoxybenzyl)-4-(1*H*-pyrrolo­[2,3-*b*]­pyridin-3-yl)­thiazol-2-amine Hydrobromide (**17**)

Compound **17** was obtained following general procedure C using **44** (185 mg, 0.82 mmol) and **45** (160 mg, 0.82 mmol), affording it as a dark-yellow solid (180 mg, 66%). ^1^H NMR (DMSO-*d*_6_, 400 MHz): δ 12.33 (br s, 1H), 9.42 (br s, 1H), 8.39–8.34 (m, 2H), 8.10 (s, 1H), 7.37–7.25 (m, 3H), 7.06–7.03 (m, 2H), 6.95 (t, *J* = 8.0 Hz, 1H), 4.63 (s, 2H), 3.82 (s, 3H). ^13^C NMR (DMSO-*d*_6_, 100 MHz): δ 169.06, 157.56, 147.03, 142.46, 130.11, 129.76, 129.29, 126.64, 124.51, 120.77, 118.13, 116.89, 111.39, 99.55, 55.96, 45.04. MS [ESI+] *m*/*z*: 337.32 [M + H]^+^.

##### *N*-(2-Chlorobenzyl)-4-(1*H*-pyrrolo­[2,3-*b*]­pyridin-3-yl)­thiazol-2-amine Hydrobromide (**18**)

Compound **18** was obtained following general procedure C using **44** (155 mg, 0.65 mmol) and **46** (130 mg, 0.65 mmol), affording it as a yellow solid (62 mg, 23%). ^1^H NMR (DMSO-*d*_6_, 400 MHz): δ 12.25 (br s, 1H), 8.94 (br s, 1H), 8.47 (d, *J* = 7.6 Hz, 1H), 8.35 (d, *J* = 4.8 Hz, 1H) 7.98 (s, 1H), 7.55–7.49 (m, 2H), 7.38–7.32 (m, 2H), 7.27–7.25 (m, 1H), 7.01 (s, 1H), 4.71 (s, 2H). ^13^C NMR (DMSO-*d*_6_, 100 MHz): δ 167.95, 140.71, 135.01, 132.38, 130.62, 129.32, 129.21, 129.05, 127.18, 125.44, 118.28, 115.93, 99.08, 45.90. MS [ESI+] *m*/*z*: 341.13 [M + H]^+^.

##### *N*-(3-Chlorobenzyl)-4-(1*H*-pyrrolo­[2,3-*b*]­pyridin-3-yl)­thiazol-2-amine Hydrobromide (**19**)

Compound **19** was obtained following general procedure C using **44** (203 mg, 0.85 mmol) and **47** (170 mg, 0.85 mmol), affording it as a yellow solid (272 mg, 76%). ^1^H NMR (DMSO-*d*_6_, 400 MHz): δ 12.40 (br s, 1H), 9.24 (br s, 1H), 8.49 (d, *J* = 8.0 Hz, 1H), 8.38 (d, *J* = 3.2, 1H), 8.08 (s, 1H), 7.52 (s, 1H), 7.42–7.35 (m, 3H), 7.33–7.30 (m, 1H), 7.06 (s, 1H), 4.67 (s, 2H). ^13^C NMR (DMSO-*d*_6_, 100 MHz): δ 168.89, 145.78, 141.07, 140.73, 133.58, 131.58, 130.86, 127.92, 127.82, 126.75, 126.51, 119.05, 116.69, 99.88, 48.28. MS [ESI+] *m*/*z*: 341.42 [M + H]^+^.

##### *N*-(4-Chlorobenzyl)-4-(1*H*-pyrrolo­[2,3-*b*]­pyridin-3-yl)­thiazol-2-amine Hydrobromide (**20**)

Compound **20** was obtained following general procedure C using **44** (150 mg, 0.63 mmol) and 1-(4-chlorobenzyl)­thiourea (127 mg, 0.63 mmol), affording it as a pale-yellow solid (147 mg, 55%). ^1^H NMR (DMSO-*d*_6_, 400 MHz): δ 12.43 (br s, 1H), 9.38 (br s, 1H), 8.46 (d, *J* = 7.2 Hz, 1H), 8.37 (d, *J* = 3.6 Hz, 1H), 8.09 (s, 1H), 7.45–7.40 (m, 4H), 7.32–7.28 (m, 1H), 7.07 (s, 1H), 4.65 (s, 2H). ^13^C NMR (DMSO-*d*_6_, 100 MHz): δ 168.95, 145.54, 141.04, 136.74, 132.53, 131.67, 130.01 (2C), 128.93 (2C), 126.77, 119.08, 116.76, 107.41, 100.14, 48.33. MS [ESI+] *m*/*z*: 341.14 [M + H]^+^.

##### *N*-(4-Fluorobenzyl)-4-(1*H*-pyrrolo­[2,3-*b*]­pyridin-3-yl)­thiazol-2-amine Hydrobromide (**21**)

Compound **21** was obtained following general procedure C using **44** (150 mg, 0.63 mmol) and **48** (116 mg, 0.63 mmol), affording it as a pale-yellow solid (52 mg, 20%). ^1^H NMR (DMSO-*d*_6_, 400 MHz): δ 12.35 (br s, 1H), 9.28 (br s, 1H), 8.47 (d, *J* = 8.0 Hz, 1H), 8.38 (d, *J* = 4.8 Hz, 1H), 8.06 (s, 1H), 7.50–7.47 (m, 2H), 7.32–7.29 (m, 1H), 7.24–7.20 (m, 2H), 7.05 (s, 1H), 4.64 (s, 2H). ^13^C NMR (DMSO-*d*_6_, 100 MHz): δ 168.88, 162.01 (d, ^1^ *J*_C–F_ = 241.5 Hz), 146.24, 141.56, 134.00, 131.08 (d, *J*_C–F_ = 6 Hz), 130.24 (d, ^3^ *J*_C–F_ = 8.4 Hz, 2C), 126.45, 118.74, 116.74, 115.75 (d, ^2^ *J*_C–F_ = 21.9 Hz, 2C), 99.77, 48.27. ^19^F NMR (DMSO-*d*_6_, 377 MHz): δ −115.09. MS [ESI+] *m*/*z*: 325.13 [M + H]^+^.

##### *N*-(4-Methylbenzyl)-4-(1*H*-pyrrolo­[2,3-*b*]­pyridin-3-yl)­thiazol-2-amine Hydrobromide (**22**)

Compound **22** was obtained following general procedure C using **44** (150 mg, 0.63 mmol) and 1-(4-methylbenzyl)­thiourea (114 mg, 0.63 mmol), affording it as a yellow solid (166 mg, 66%). ^1^H NMR (DMSO-*d*_6_, 400 MHz): δ 12.34 (br s, 1H), 9.45 (br s, 1H), 8.42 (d, *J* = 8.0 Hz, 1H), 8.38 (d, *J* = 4.4 Hz, 1H), 8.08 (s, 1H), 7.33–7.28 (m, 3H), 7.20 (d, *J* = 8.4 Hz, 2H), 7.06 (s, 1H), 4.62 (s, 2H), 2.29 (s, 3H). ^13^C NMR (DMSO-*d*_6_, 100 MHz): δ 169.01, 146.57, 142.01, 137.30, 134.20, 130.61, 129.56 (2C), 129.42, 128.18 (2C), 127.96, 126.62, 118.46, 116.84, 99.78, 48.99, 21.15. MS [ESI+] *m*/*z*: 321.14 [M + H]^+^.

##### *N*-(4-Methoxybenzyl)-4-(1*H*-pyrrolo­[2,3-*b*]­pyridin-3-yl)­thiazol-2-amine Hydrobromide (**23**)

Compound **23** was obtained following general procedure C using **44** (150 mg, 0.63 mmol) and 1-(4-methoxybenzyl)­thiourea (124 mg, 0.63 mmol), affording it as a pale-yellow solid (199 mg, 76%). ^1^H NMR (DMSO-*d*_6_, 400 MHz): δ 12.39 (br s, 1H), 9.57 (br s, 1H), 8.43 (d, *J* = 7.6 Hz, 1H), 8.38 (d, *J* = 5.2 Hz, 1H), 8.13 (s, 1H), 7.38 (d, *J* = 8.8 Hz, 2H), 7.32–7.29 (m, 1H), 7.09 (s, 1H), 6.95 (d, *J* = 8.8 Hz, 2H), 4.61 (s, 2H), 3.74 (s, 3H). ^13^C NMR (DMSO-*d*_6_, 100 MHz): δ 168.91, 159.30, 146.62, 142.11, 130.52, 129.72 (2C), 128.89, 126.76, 118.39, 116.88, 114.43 (2C), 106.34, 99.82, 55.59, 48.81. MS [ESI+] *m*/*z*: 337.24 [M + H]^+^.

##### *N*-(4-Nitrobenzyl)-4-(1*H*-pyrrolo­[2,3-*b*]­pyridin-3-yl)­thiazol-2-amine Hydrobromide (**24**)

Compound **24** was obtained following general procedure C using **44** (102 mg, 0.43 mmol) and **49** (90 mg, 0.43 mmol), affording it as a green solid (127 mg, 68%). ^1^H NMR (DMSO-*d*_6_, 400 MHz): δ 12.46 (br s, 1H), 9.35 (br s, 1H), 8.50 (d, *J* = 8 Hz, 1H), 8.38 (d, *J* = 4 Hz, 1H), 8.12 (s, 1H), 7.48–7.41 (m, 4H), 7.33–7.25 (m, 1H), 7.09 (s, 1H), 4.70 (s, 2H). ^13^C NMR (DMSO-*d*_6_, 100 MHz): δ 168.72, 147.16, 146.93, 145.36, 140.53, 132.05, 128.96 (2C), 126.28, 124.05 (2C), 119.36, 116.53, 99.91, 48.05. MS [ESI+] *m*/*z*: 352.23 [M + H]^+^.

##### *N*-(4-(Dimethylamino)­benzyl)-4-(1*H*-pyrrolo­[2,3-*b*]­pyridin-3-yl)­thiazol-2-amine Hydrobromide (**26**)

Compound **26** was obtained following general procedure C using **44** (198 mg, 0.83 mmol) and **50** (173 mg, 0.83 mmol), affording it as a light-blue solid (70 mg, 20%). mp 155–156 °C. ^1^H NMR (DMSO-*d*_6_, 400 MHz): δ 12.11 (br s, 1H), 9.01 (br s, 1H), 8.39 (d, *J* = 8.0 Hz, 1H), 8.33 (d, *J* = 4.0 Hz, 1H), 7.97 (s, 1H), 7.39 (d, *J* = 8.0 Hz, 2H), 7.25–7.22 (m, 1H), 7.10–7.09 (m, 2H), 6.97 (s, 1H), 4.55 (s, 2H), 2.99 (s, 6H). ^13^C NMR (DMSO-*d*_6_, 100 MHz): δ 168.58, 147.71, 147.69, 142.67, 132.87, 129.78, 129.75, 129.38, 125.76, 117.97, 116.63, 116.05, 115.63, 99.07, 48.43, 42.52. MS [ESI+] *m*/*z*: 350.42 [M + H]^+^.

##### *N*-(Pyridin-4-ylmethyl)-4-(1*H*-pyrrolo­[2,3-*b*]­pyridin-3-yl)­thiazol-2-amine Hydrobromide (**27**)

Compound **27** was obtained following general procedure C using **44** (143 mg, 0.60 mmol) and **51** (100 mg, 0.60 mmol), affording it as a yellowish solid (170 mg, 73%). ^1^H NMR (DMSO-*d*_6_, 400 MHz): δ 11.86 (br s, 1H), 8.83 (d, *J* = 6.4 Hz, 2H), 8.57 (br s, 1H), 8.22–8.19 (m, 2H), 7.97 (d, *J* = 6.4 Hz, 2H), 7.75 (s, 1H), 7.09 (t, *J* = 6.4 Hz, 1H), 6.90 (s, 1H), 4.82 (s, 2H). ^13^C NMR (DMSO-*d*_6_, 100 MHz): δ 167.64, 159.88, 148.15, 145.29, 142.93 (2C), 142.55, 129.58, 125.17 (2C), 124.85, 117.88, 116.26, 110.63, 99.23, 47.40. MS [ESI+] *m*/*z*: 308.20 [M + H]^+^.

##### *N*-((2-Chloropyridin-4-yl)­methyl)-4-(1*H*-pyrrolo­[2,3-*b*]­pyridin-3-yl)­thiazol-2-amine Hydrobromide (**28**)

Compound **28** was obtained following general procedure C using **44** (96 mg, 0.4 mmol) and **52** (80 mg, 0.4 mmol), affording it as a yellow solid (89 mg, 53%). mp 164–165 °C. ^1^H NMR (DMSO-*d*_6_, 400 MHz): δ 12.41 (br s, 1H), 8.90 (br s, 1H), 8.50 (d, *J* = 7.6 Hz, 1H), 8.36 (d, *J* = 5.2 Hz, 2H), 8.00 (s, 1H), 7.53 (s, 1H), 7.43 (d, *J* = 4.8 Hz, 1H), 7.31–7.28 (m, 1H), 7.02 (s, 1H), 4.66 (s, 2H). ^13^C NMR (DMSO-*d*_6_, 100 MHz): δ 168.58, 152.56, 150.87, 150.39, 144.68, 139.92, 132.73, 126.31, 123.04 (2C), 122.23 (2C), 119.74, 116.48, 100.21, 47.07. MS [ESI+] *m*/*z*: 342.23 [M + H]^+^.

##### *N*-(Pyrimidin-2-ylmethyl)-4-(1*H*-pyrrolo­[2,3-*b*]­pyridin-3-yl)­thiazol-2-amine Hydrobromide (**29**)

Compound **29** was obtained following general procedure C using **44** (185 mg, 0.77 mmol) and **53** (130 mg, 0.77 mmol), affording it as a light-green solid (220 mg, 73%). ^1^H NMR (DMSO-*d*_6_, 400 MHz): δ 12.39 (br s, 1H), 9.58 (br s, 1H), 8.84 (d, *J* = 5.2 Hz, 2H), 8.43 (d, *J* = 8.4 Hz, 1H), 8.37 (d, *J* = 5.2 Hz, 1H), 8.11 (s, 1H), 7.47 (t, *J* = 5.2 Hz, 1H), 7.32–7–7.29 (m, 1H), 7.10 (s, 1H), 4.94 (s, 2H). ^13^C NMR (DMSO-*d*_6_, 100 MHz): δ 168.93, 165.06, 157.45 (2C), 145.78, 141.20, 137.13, 130.16, 126.00, 120.12, 117.93, 116.18, 106.14, 99.30, 50.30. MS [ESI+] *m*/*z*: 309.30 [M + H]^+^.

##### *N*-(Pyrazin-2-ylmethyl)-4-(1*H*-pyrrolo­[2,3-*b*]­pyridin-3-yl)­thiazol-2-amine Hydrobromide (**30**)

Compound **24** was obtained following general procedure C using **44** (185 mg, 0.77 mmol) and **54** (130 mg, 0.77 mmol), affording it as a brownish solid (220 mg, 73%). ^1^H NMR (DMSO-*d*_6_, 400 MHz): δ 12.46 (br s, 1H), 9.27 (br s, 1H), 8.78 (s, 1H), 8.64 (s, 1H), 8.57 (d, *J* = 2.0 Hz, 1H), 8.51 (d, *J* = 8.0 Hz, 1H), 8.38 (d, *J* = 5.0 Hz, 1H), 8.10 (s, 1H), 7.32 (dd, ^1^ *J* = 5.0 Hz, ^2^ *J* = 8.0 Hz, 1H), 7.08 (s, 1H), 4.86 (s, 2H). ^13^C NMR (DMSO-*d*_6_, 400 MHz): δ 169.04, 153.32, 145.26, 144.53, 144.26, 144.07, 140.59, 132.00, 126.64, 119.28, 116.66, 100.10, 48.32. MS [ESI+] *m*/*z*: 309.30 [M + H]^+^.

##### *N*-Benzyl-*N*-methyl-4-(1*H*-pyrrolo­[2,3-*b*]­pyridin-3-yl)­thiazol-2-amine Hydrobromide (**31**)

Compound **31** was obtained following general procedure C using **44** (225 mg, 0.94 mmol) and **55** (170 mg, 0.94 mmol), affording it as a yellow solid (118 mg, 31%). ^1^H NMR (DMSO-*d*_6_, 400 MHz): δ 12.50 (br s, 1H), 8.70 (d, *J* = 8.0 Hz, 1H), 8.39 (d, *J* = 5.2 Hz, 1H), 8.05 (s, 1H), 7.37–7.29 (m, 5H), 7.28–7.26 (m, 1H), 7.10 (s, 1H), 4.79 (s, 2H), 3.12 (s, 3H). ^13^C NMR (DMSO-*d*_6_, 100 MHz): δ 170.09, 139.09, 137.12, 133.63, 129.07 (2C), 127.96 (2C), 127.92, 126.57, 120.38, 116.52, 100.40, 56.41, 39.16. MS [ESI+] *m*/*z*: 321.23 [M + H]^+^.

##### *N*-Phenethyl-4-(1*H*-pyrrolo­[2,3-*b*]­pyridin-3-yl)­thiazol-2-amine Hydrobromide (**32**)

Compound **32** was obtained following general procedure C using **44** (259 mg, 1.08 mmol) and **56** (195 mg, 1.08 mmol), affording it as a light-yellow solid (321 mg, 74%). ^1^H NMR (DMSO-*d*_6_, 400 MHz): δ 12.29 (br s, 1H), 9.23 (br s, 1H), 8.40–8.35 (m, 2H), 8.06 (s, 1H), 7.33–7.21 (m, 6H), 7.04 (s, 1H), 3.69 (t, *J* = 7.0 Hz, 2H), 2.96 (t, *J* = 7.0 Hz, 2H). ^13^C NMR (DMSO-*d*_6_, 100 MHz): δ 168.89, 147.28, 142.69, 138.91, 129.85, 129.28 (2C), 128.88 (2C), 126.91, 126.60, 118.03, 116.83, 99.43, 47.43, 34.59. MS [ESI+] *m*/*z*: 321.23 [M + H]^+^.

##### *N*-(4-(1*H*-Pyrrolo­[2,3-*b*]­pyridin-3-yl)­thiazol-2-yl)­benzamide Hydrobromide (**33**)

Compound **33** was obtained following general procedure C using **44** (186 mg, 0.78 mmol) and **57** (140 mg, 0.78 mmol), affording it as a yellowish solid (220 mg, 70%). ^1^H NMR (DMSO-*d*_6_, 400 MHz): δ 12.59 (br s, 2H), 9.00 (d, *J* = 8.0 Hz, 1H), 8.46 (d, *J* = 4.0 Hz, 1H), 8.10 (d, *J* = 7.6 Hz, 2H), 7.63–7.52 (m, 5H), 7.47–7.44 (m, 1H). ^13^C NMR (DMSO-*d*_6_, 100 MHz): δ 165.64, 158.66, 143.94, 143.17, 138.37, 134.68, 133.09, 132.49, 129.04, 128.62, 126.34, 120.94, 116.46, 117.77, 107.02. MS [ESI+] *m*/*z*: 321.21 [M + H]^+^.

##### *N*-Benzyl-4-(5-bromo-1*H*-pyrrolo­[2,3-*b*]­pyridin-3-yl)­thiazol-2-amine Hydrobromide (**38**)

Compound **38** was obtained following general procedure C using **74** (140 mg, 0.44 mmol) and *N*-benzyl thiourea (70 mg, 0.44 mmol), affording it as a yellow solid (98 mg, 48%). ^1^H NMR (DMSO-*d*_6_, 400 MHz): δ 12.37 (br s, 1H), 9.78 (br s, 1H), 8.46 (d, *J* = 2.3 Hz, 1H), 8.37 (d, *J* = 2.2 Hz t, 1H), 8.12 (d, *J* = 2.8 Hz, 1H), 7.45–7.38 (m, 4H), 7.32 (t, *J* = 7 Hz, 1H), 7.13 (s, 1H), 4.69 (s, 2H). ^13^C NMR (DMSO-*d*_6_, 400 MHz): δ 169.14, 147.21, 144.19, 136.90, 130.39, 129.10 (2C), 128.20, 128.18 (2C), 127.99, 118.85, 112.27, 100.20, 49.37. MS [ESI+] *m*/*z*: 387.10 [M + H]^+^.

##### *N*-Benzyl-5-methyl-4-(1*H*-pyrrolo­[2,3-*b*]­pyridin-3-yl)­thiazol-2-amine Hydrobromide (**39**)

Compound **39** was obtained following general procedure C using **75** (137 mg, 0.51 mmol) and *N*-benzyl thiourea (80 mg, 0.51 mmol), affording it as a yellowish solid (85 mg, 42%). ^1^H NMR (DMSO-*d*_6_, 400 MHz): δ 12.36 (br s, 1H), 9.89 (br s, 1H), 8.34 (d, *J* = 4.9 Hz, 1H), 8.01 (d, *J* = 8.0 Hz, 1H), 7.85 (s, 1H), 7.41 (d, *J* = 4.6 Hz, 4H), 7.36–7.32 (m, 1H), 7.21–7.18 (m, 1H), 4.65 (s, 2H), 2.24 (s, 2H). ^13^C NMR (DMSO-*d*_6_, 100 MHz): δ 166.02, 146.58, 142.32, 136.35, 129.35, 129.31, 128.53 (2C), 127.60, 127.55 (2C), 127.10, 119.44, 118.68, 116.12, 113.60, 48.20, 11.58. MS [ESI+] *m*/*z*: 321.19 [M + H]^+^.

##### *N*-Benzyl-4-(5-bromo-1*H*-pyrrolo­[2,3-*b*]­pyridin-3-yl)-5-methylthiazol-2-amine Hydrobromide (**41**)

Compound **41** was obtained following general procedure C using **76** (120 mg, 0.36 mmol) and *N*-benzyl thiourea (60 mg, 0.36 mmol), affording it as a light-brown solid (138 mg, 80%). mp 254–256 °C. ^1^H NMR (DMSO-*d*_6_, 400 MHz): δ 12.43 (br s, 1H), 9.90 (br s, 1H), 8.37 (d, *J* = 2.2 Hz, 1H), 8.24 (s, 1H), 7.90 (d, *J* = 2.8 Hz, 1H), 7.45–7.42 (m, 4H), 7.40–7–32 (m, 1H), 4.64 (s, 2H), 2.24 (s, 3H). ^13^C NMR (DMSO-*d*_6_, 100 MHz): δ 166.62, 146.84, 144.07, 136.90, 136.84, 130.73, 129.11 (2C), 128.20, 128.06 (2C), 121.62, 120.34, 114.54, 111.87, 48.79, 12.09. MS [ESI+] *m*/*z*: 399.24 [M + H]^+^.

##### 5-Methyl-*N*-(pyridin-4-ylmethyl)-4-(1*H*-pyrrolo­[2,3-*b*]­pyridin-3-yl)­thiazol-2-amine Hydrobromide (**42**)

Compound **42** was obtained following general procedure C using **75** (152 mg, 0.6 mmol) and **51** (100 mg, 0.6 mmol), affording it as a pinkish solid (84 mg, 35%). ^1^H NMR (DMSO-*d*_6_, 400 MHz): δ 11.87 (br s, 1H), 8.82 (d, *J* = 6.6 Hz, 2H), 8.43 (br s, 1H), 8.19 (dd, ^1^ *J* = 4.7 Hz, ^2^ *J* = 1.7 Hz, 1H), 7.92 (d, *J* = 6.6 Hz, 2H), 7.82 (dd, ^1^ *J* = 7.9 Hz, ^2^ *J* = 1.7 Hz, 1H), 7.57 (s, 1H), 6.96 (dd, ^1^ *J* = 7.9 Hz, ^2^ *J* = 4.7 Hz, 1H), 4.75 (s, 2H), 2.30 (s, 3H). ^13^C NMR (DMSO-*d*_6_, 100 MHz): δ 164.35, 159.59, 147.70, 143.47 (2C), 142.70, 139.91, 130.37, 124.87 (2C), 124.74, 119.32, 115.83, 113.39, 109.25, 47.15, 12.37. MS [ESI+] *m*/*z*: 322.23 [M + H]^+^.

##### *N*-((2-Chloropyridin-4-yl)­methyl)-5-methyl-4-(1*H*-pyrrolo­[2,3-*b*]­pyridin-3-yl)­thiazol-2-amine Hydrobromide (**43**)

Compound **43** was obtained following general procedure C using **75** (101 mg, 0.40 mmol) and **52** (80 mg, 0.40 mmol), affording it as a yellow solid (120 mg, 69%). mp 190–191 °C. ^1^H NMR (DMSO-*d*_6_, 400 MHz): δ 12.25 (br s, 1H), 8.95 (br s, 1H), 8.42 (d, *J* = 5.2 Hz, 1H), 8.33 (dd, ^1^ *J* = 5.0 Hz, ^2^ *J* = 1.2 Hz, 1H), 8.07 (d, *J* = 8.0 Hz, 1H), 7.75 (d, *J* = 2.0 Hz, 1H), 7.54 (s, 1H), 7.44 (dd, ^1^ *J* = 5.0 Hz, ^2^ *J* = 1.4 Hz, 1H), 7.18 (dd, ^1^ *J* = 5.0 Hz, ^2^ *J* = 8.0 Hz, 1H), 4.64 (s, 2H), 2.32 (s, 3H). ^13^C NMR (DMSO-*d*_6_, 100 MHz): δ 166.02, 151.90, 150.94, 150.48, 141.19, 131.84, 131.32, 127.02, 122.95, 122.07, 120.36, 116.28, 114.63. 46.86. 12.21. MS [ESI+] *m*/*z*: 356.15 [M + H]^+^.

##### *N*-Benzyl-5-(1*H*-pyrrolo­[2,3-*b*]­pyridin-3-yl)-1,3,4-thiadiazol-2-amine (**40**)

In a pressure tube, a mixture of **77** (100 mg, 0.7 mmol) and **78** (317 mg, 1.75 mmol) was dissolved in trifluoroacetic acid (2.5 mL), refluxed for 3 h, and monitored by TLC. After completion, the reaction was cooled down to room temperature and diluted with water (3 mL). Then, 30% ammonia aqueous solution was added dropwise until pH = 8. The resulting precipitate was filtered, washed with EtOAc, and dried to obtain the pure product **40** as a yellow solid (156 mg, 71%). mp 265–267 °C. ^1^H NMR (DMSO-*d*_6_, 400 MHz): δ 12.17 (br s, 1H), 8.43 (dd, ^1^ *J* = 7.8 Hz, ^2^ *J* = 1.5 Hz, 1H), 8.32–8.26 (m, 2H), 8.00 (s, 1H), 7.40–7.33 (m, 4H), 7.28–7.19 (m, 2H), 4.54 (d, *J* = 4.8 Hz, 2H). ^13^C NMR (DMSO-*d*_6_, 100 MHz): δ 166.59, 152.07, 149.08, 144.43, 139.25, 129.54, 128.80 (2C), 127.97 (2C), 127.53, 127.30, 117.34, 117.10, 106.60, 48.51. MS [ESI+] *m*/*z*: 308.09 [M + H]^+^.

### Kinase Assays

GSK-3β and FYNα kinase assays were run in 384-well microplates (OptiPlate-384, White, PerkinElmer) in a total reaction volume of 20 μL. The inhibitory potency against human recombinant GSK-3β and FYNα (Carna Biosciences) was evaluated using the LANCE Ultra (PerkinElmer) time-resolved fluorescence resonance energy transfer (TR-FRET) by measuring the phosphorylation of the ULight-labeled substrate, according to the manufacturer’s instructions. The synthetic peptide surrounding Ser641 of human muscle glycogen synthase (ULight-GS (Ser641/pSer657)) and the synthetic 28-amino acid peptide containing eight Tyr residues placed in different amino acid contexts (ULightTM-TK (PT66)) were selected as specific substrates for GSK-3β and FYNα, respectively. Briefly, test compounds and staurosporine (reference compound) or DMSO (control) are mixed with the enzyme (GSK-3β: 2 nM and FYNα: 1 nM) in a buffer containing 50 mM HEPES (pH 7.5), 1 mM EGTA, 10 mM MgCl_2_, 2 mM DTT, and 0.01% Tween-20. The reaction is initiated by adding 50 nM of the substrate and ATP at a final concentration, determined experimentally for each new ATP stock solution prepared, near the *K*_m_ value of the enzyme for ATP (e.g., GSK-3β: 1.2 μM or FYN-α: 8 μM), and the mixture is incubated for 60 or 90 min, respectively, at 23 °C. Following incubation, the reaction is stopped by adding 6 mM EDTA. After 5 min, the antiphospho antibody labeled with europium chelate is added. After 1 more hour, the kinase reaction is monitored by irradiation at 320 nm and the fluorescence measured at 615 and 665 nm, using EnVision 2014 Multilabel Reader (PerkinElmer). The calculated signal ratio at 665/615 nm is proportional to the extent of ULight-substrate phosphorylation. The compounds were tested at 11 different concentrations ranging from 3 nM up to 100 μM in triplicate. For a few compounds with poor solubility or potency, percentage of inhibition at one concentration (50 μM) was determined. The results were expressed as a percent inhibition of the control enzyme activity.

To study the GSK-3β and FYNα kinetics, the reaction mixture, varying concentrations of ATP (0.75, 1.5, 3, 6, and 12 μM or 1.5, 3, 6, 12, and 24 μM for GSK-3β and FYNα, respectively) versus test compound 43 (0.75 μM and 4.5 μM against GSK-3β and 0.05 and 0.3 μM against FYNα) were incubated for 5, 15, 30, and 60 min (and 90 min for FYNα only) at 23 °C, followed by the addition of 6 mM EDTA and the antiphospho-GS or antiphospho-TK antibodies, for GSK-3β and FYNα respectively, according to the manufacturer’s protocol. Initial velocities (*V*_0_) were determined and fitted to the Michaelis–Menten equation. To directly visualize the compound **43** inhibition mode, a Lineweaver–Burk plot was generated according to the values obtained from the Michaelis–Menten analysis. The slope corresponded to *K*_m_/*V*_max_, the intercept on the vertical axis corresponded to 1/*V*_max_, and the intercept on the horizontal axis corresponded to −1/*K*_m_. Moreover, at the reciprocal of the smallest value of substrate concentration (*X* = 1/[*S*_min_]) was associated a value of *Y* representing the equation *Y* = (1/*V*_max_)­(1.0 + *K*_m_/[*S*_min_]). Graphs and data analysis were performed by using GraphPad Prism 8 software. The kinase selectivity profiles of compounds **41** and **43** were evaluated at single concentration (i.e., 10.0 μM) using the gold standard radiometric kinase assay through the Eurofins KinaseProfiler platform.

#### Analysis of the Biological Data

Dose–response curves were run in at least three independent experiments, performed in three technical replicates. IC_50_ values (concentrations causing half-maximal enzyme inhibition) were determined by nonlinear regression analysis of the Log­[concentration]/response curves generated with mean replicate values using a four parameter Hill equation curve fitting with GraphPad Prism 8 (GraphPad Software Inc., CA-USA).

### Computational Studies

The structures 2DQ7 ^44^ for FYN and 6Y9R^45^ for GSK-3β were retrieved from the Protein Data Bank and aligned for comparative analysis of docking results. Subsequently, the proteins underwent preparation using the Protein Preparation Wizard tool within the Schrödinger suite (Maestro Version 13.1.141, MMshare Version 5.7.141, Release 2022-1), employing default parameters and the OPLS4 force field for minimization.

Grids for docking were constructed using Glide, with a center on staurosporine for FYN and 1*H*-indazole-3-carboxamide inhibitor for GSK-3β. The grid size was chosen to accommodate ligands of up to 20 Å in length. Ligands were sketched in Maestro and subjected to ligand preparation using the LigPrep tool, employing the OPLS4 force field for minimization. Various protonation states at pH 7 ± 2, as well as tautomers and stereoisomers, were generated. The prepared ligand library underwent molecular docking on both proteins using Glide in SP mode.

Subsequently, complexes obtained from traditional docking for compounds **28**, **40**, **41**, and **43** were utilized as a starting point for molecular dynamics simulations. Each system was built using the System Builder tool, employing the TIP4P solvation model within an orthorhombic box with a 10 Å margin. Automatic neutralization and the OPLS4 force field were applied.

Molecular dynamics simulations were conducted with Desmond for 100 ns, saving coordinates every 100 ps at 300 K and 1.01325 bar (*NPT* ensemble). Before the simulation, each system was relaxed using the default protocol within the software. Trajectory analysis was performed using the Simulation Interaction Diagram tool. Binding mode images were generated using PyMOL (ver. 2.5.0), while interaction profiles over time were extracted from the report generated by the Simulation Interaction Diagram tool, focusing on the last 50 ns of simulation.

### Biological Evaluation

#### Cell Viability in Primary Cerebellar Granule Cells (CGNs)

Primary cerebellar granule cells were dissociated from cerebella and plated on 96-well plates, previously coated with 10 μg/mL poly-l-lysine, at a density of 1.2 × 10^5^ cells/0.2 mL medium/well in BME supplemented with 10% heat-inactivated FBS (Life Technologies), 2 mmol/L glutamine, 100 μmol/L gentamicin sulfate, and 25 mM KCl (all from Sigma-Aldrich). Sixteen hours later, 10 μM cytosine arabinofuranoside (Sigma-Aldrich) was added to avoid glial proliferation. After 7 days *in vitro*, differentiated neurons were shifted to serum-free BME medium and exposed to increasing concentrations (5 μM, 10 μM, 25 μM) of compounds of interest for 24 h. Then, cell viability was evaluated through the MTT assay.^39^

#### Neuroprotection in CGNs

To evaluate the neuroprotective effect of our compounds, we tested increasing concentrations (5 μM, 10 μM, 25 μM) of compounds of interest in differentiated CGNs switched to serum-free BME medium with 5 mM KCl (serum-potassium deprivation) for 48 h to mimic in vitro the naturally occurring death of granule cells.^39^ Neuroprotection was evaluated through MTT assay.^46^

#### MTT Assay

For MTT assay, thiazolyl blue was added to the culture medium at a final concentration of 0.1 mg/mL. Following a 20 min incubation at 37 °C in the dark, the MTT precipitate was dissolved in 0.1 M Tris–HCl pH 7.5 buffer containing 5% Triton X-100 (all from Sigma-Aldrich) and absorbance was read at 570 nm in a microplate spectrophotometric reader (Bio-Rad).

#### Immunomodulation in N9 Cell Line

N9 microglial cells were plated at a density of 2.5 × 10^5^ cells/35 mm Ø dish in serum-free DMEM High Glucose (Life Technologies) and pretreated (2 h) with increasing concentrations of compounds (2.5 and 5 μM) in the presence of 100 ng/mL LPS for 24 h. The microglial phenotype was evaluated through Western blot analysis of the pro-inflammatory iNOS and anti-inflammatory-TREM2 markers.

#### Neurosphere Assays

NSCs were initially obtained by SVZ microdissection of 6 months-old C57BL/6N wild-type male mice (*Mus musculus*) and routinely cultured in suspension in DMEM-F12 (Gibco; ThermoFisher Scientific, Waltham, MA, USA) supplemented with 2 mM glutamine, 10 μg/mL insulin from bovine pancreas (Sigma-Aldrich; St Louis, MO, USA), 20 ng/mL epidermal growth factor (EGF; PeproTech EC, London, UK), 20 ng/mL fibroblast growth factor-2 (FGF2; PeproTech), 1% N2 (ThermoFisher Scientific), 1% B27 (ThermoFisher Scientific), 10 units/mL penicillin, and 10 μg streptomycin.^47^

To investigate the effect of compounds of interest in neurospheres’ growth rate, single spheres were plated in suspension in 96-well plates (5 × 10^3^ cells/well) in the presence of compounds (0.1 μM, 1 μM, and 5 μM) in complete DMEM F-12 culture medium. One image/well was acquired every day by using the Sartorius Incucyte Live-Cell Analysis System and evaluated through the Brightfield Spheroid Analysis Software Module. Only aggregates with areas larger than 400 μm^2^ were considered for statistical analysis.

To assess the effect of compounds of interest in NSCs differentiation, 30 spheres were plated on 13 mm glass coverslips, previously coated with Matrigel matrix (Corning; New York, USA), in complete DMEM F-12 medium, and treated with 1 μM of compounds of interest. After 7DIV, neurospheres were fixed for 20 min with 4% PFA in PBS 0.1% pH 7.4. Fixed neurospheres were permeabilized in 0.1% Triton/PBS and nonspecific sites were blocked for 1 h with 0.1% Triton/PBS and 5% normal goat serum (Sigma-Aldrich; St Louis, MO, USA). Cells were then incubated overnight at 4 °C with the following primary antibodies: Anti-Doublecortin DCX (Abcam, Cambridge, UK), GFAP (Dakopatts), OLIG2 (Santa Cruz Biotechnology), 1:500 dilution in 0.1% Triton/PBS with 2% normal goat serum. After 3 washes in 0.1% Triton/PBS, specific secondary antibodies were added for 2 h at RT in the dark: Donkey anti-Mouse IgG Alexafluor 555 (Abcam, Cambridge, UK), Goat anti-Rabbit IgG Alexafluor 555 (Abcam, Cambridge, UK), 1:1000 dilution in 0.1% Triton/PBS with 2% normal goat serum. Following 3 washes in 0.1% Triton/PBS, nuclei were stained with DAPI (2 μg/mL, Sigma-Aldrich) for 5 min. Glass coverslips were mounted by using Ultracruz Aqueous Mounting Medium with DAPI (Santa Cruz Biotechnology). Neurospheres confocal images were obtained with a Nikon EZ-C1 microscope (60× objective) and the z-stack function (1024 steps and 1 μm thickness layers; 40 total stacks). 3D image reconstruction was performed by using Fiji ImageJ2 software, z-project plugin, and selecting the sum stacks’ function. Fluorescence intensity index was estimated as the ratio of markers’ positive cells intensity/total cells fluorescence intensity stained with DAPI per ROI.^48^

#### Statistical Analysis

Data were analyzed by using the GraphPad Prism8, San Diego, CA, United States, software and expressed as the mean ± standard error of independent experiments. One-way ANOVA followed by Dunnett’s post hoc was used to compare the means between control and treated cells. Only *p*-values < 0.05 were considered statistically significant.

### BBB Permeability

BBB permeability assay was conducted as described in Albertini et al.^39^ The brain microvascular endothelial cells (hCMEC/D3) were seeded at the density of 5 × 10^4^ cells per cm^2^ in the upper compartment of Transwell filters (polyester, pore size 0.4 μm, 12-well tissue culture transwell; Corning), precoated with rat tail collagen type I (0.1 mg/mL; Roche). They were grown for 7 days up to confluence in Endothelial Cell Growth Medium 2 (Promocell), supplemented with penicillin/streptomycin 100 U/mL, in a humidified, 5% CO_2_ incubator at 37 °C. Confluent monolayers were washed in prewarmed HBSS buffer, and the selected compounds were added to the apical (AP) side at a concentration of 100 μM. The permeability coefficient of tetramethylrhodamine isothiocyanate (TRITC)-dextran (average mol wt 4400; Sigma-Aldrich), considered as a parameter of paracellular transport and of tight junction integrity,^49^ was measured. Samples (100 μL) were collected from the BL side at different time points (15, 30, 60, and 90 min) and then replaced with the corresponding volume of HBSS buffer. TRITC-dextran flux had initially been monitored over a duration of 300 min but, at incubation times longer than 90 min, cytotoxic effects on hCMEC/D3 cells were observed in accordance with a previous report.^50^ The concentration of compounds was determined using a Ultimate 3000 HPLC system (ThermoFisher) with UV–vis detection at the maximum absorption wavelength of each compound except for inulin as determined by a spectrophotometric method using a Tecan Sunrise absorbance microplate reader.^51^ The amount of TRITC-dextran was quantified using an Infinite 200 PRO microplate reader (Tecan) with excitation and emission at 540 and 590 nm, respectively. The permeability coefficient (Papp) was calculated as follows: Papp = (Δ*Q*/Δ*t*)/(*C*_[donor]_ × *A*), where Δ*Q*/Δ*t* represents the permeation rate (mol/s), *C*_[donor]_ is the initial compound concentration in the donor compartment at time 0 (mol/cm^3^), and A represents the surface area of the filter (1.12 cm^2^).

## Supplementary Material





## Notes

Supplementary chemistry material; 3D binding poses of compounds **28** and **43**; Western blot analysis of Fyn and GSK-3β expression in exploited cell lines; neurotoxicity and neuroprotective profiles of compounds **21**, **27**, **33**, **38**, **39** and **42**; NPC proliferation analysis for compounds **27**, **39** and **42**; physicochemical parameters of compounds **28** and **43**; permeability coefficients of compounds **28** and **43**; kinase inhibition profiles of compounds **41** and **43**; ^1^H, ^13^C NMR spectra, and UHPLC-MS traces of compounds **26**, **28**, **40**, **41**, and **43** (PDF)
Molecular formula strings containing SMILES string and associated biochemical and biological data (CSV)

## ∇

Structural Biophysics Facility, Istituto Italiano di Tecnologia, Via Morego, 30, 16163 Genova, Italy

## Abbreviations

ANOVA: analysis of variance
APP: amyloid precursor protein
BBB: blood–brain barrier
BME: basal medium eagle
CGN: cerebellar granule neurons
CNS: central nervous system
DAPI: 4′,6-diamidino-2-phenylindole
DCM: dichloromethane
DCX: doublecortin
DMEM: Dulbecco’s modified eagle medium
DMSO: dimethyl sulfoxide
DTT: 1,4-dithiothreitol
Fyn: Fyn proto-oncogene kinase
EDTA: ethylenediaminetetraacetic acid
EGTA: egtazic acid
FBS: fetal bovine serum
GAPDH: glyceraldehyde-3-phosphate dehydrogenase
GFAP: glial fibrillary acidic protein
GSK-3β: glycogen synthase kinase 3β
HBSS: Hanks’ balanced salt solution
HPLC: high-performance liquid chromatography
iNOS: inducible nitric oxide synthase
LPS: lipopolysaccharide
MTT: 3-(4,5-dimethylthiazol-2-yl)-2,5-diphenyltetrazolium bromide
MW: microwave
NFT: neurofibrillary tangles
NMR: nuclear magnetic resonance
NPC: neural progenitor cell
PBS: phosphate-buffered saline
OLIG2: oligodendrocyte transcription factor 2
SAR: structure–activity relationship
SVZ: subventricular zone
TLC: thin-layer chromatography
TMS: tetramethyl silane
TREM2: triggering receptor expressed on myeloid cells 2
TRITC: tetramethyl-rhodamine isothiocyanate
