# Supplementary material for: Exploration of the Neuromodulatory Properties of Fyn and GSK-3β Kinases Exploiting 7‑Azaindole-Based Inhibitors
Source: J Med Chem. 2025 Aug 8;68(16):17130–54. doi: 10.1021/acs.jmedchem.5c00629 (PMC12406253; doi:10.1021/acs.jmedchem.5c00629)
Supplement: Supplementary file 1 [file jm5c00629_si_001.pdf]

Supporting Information

Exploration of the neuromodulatory properties of Fyn and GSK-3*β* kinases exploiting 7-azaindole-based inhibitors

Giambattista Marotta[a], Francesca Massenzio[b], Jose Ortega[c], Debora Russo[d,h], Ilaria Penna[e], Federico Falchi[a,c], Giorgia Babini[b], Sabrina Petralla[f], Rita Scarpelli[e], Elena Roggiolani[a], Gert Fricker[f], Michela Rosini[a], Andrea Cavalli[a,c], Barbara Monti[b,g], Anna Minarini[a,*], Filippo Basagni[a,*]

[a] Department of Pharmacy and Biotechnology, Alma Mater Studiorum – University of Bologna, Via Belmeloro 6, 40126 – Bologna (Italy)

[b] Department of Pharmacy and Biotechnology, Alma Mater Studiorum – University of Bologna, Via Selmi 3, 40126 – Bologna (Italy)

[c] Computational and Chemical Biology, Istituto Italiano di Tecnologia, Via Morego 30, 16163 - Genova (Italy)

[d] D3 Pharma Chemistry, Istituto Italiano di Tecnologia, via Morego 30, 16163 - Genova (Italy)

[e] Medicinal Chemistry and Technologies for Drug Discovery and Delivery Facility, Istituto Italiano di Tecnologia, via Morego 30, 16163 - Genova (Italy)

[f] Institute of Pharmacy and Molecular Biotechnology, Ruprecht-Karls-University, Im Neuenheimer Feld 364, 69120 - Heidelberg (Germany)

[g] IRCCS Istituto delle Scienze Neurologiche di Bologna, Via Altura 3, 40139 - Bologna (Italy)

[h] Present: Structural Biophysics Facility, Istituto Italiano di Tecnologia, Via Morego, 30, 16163 - Genova (Italy)

Corresponding authors:

* Anna Minarini: anna.minarini@unibo.it, Phone: +39 051 2099709;

* Filippo Basagni: filippo.basagni2@unibo.it, Phone: +39 051 2099744.

Table of contents:

Supplementary Chemistry Material page S2

Figures and Tables page S9

NMR spectra and UHPLC traces of final compounds **26**, **28**, **40**, **41** and **43** page S15

Supplementary Chemistry Material

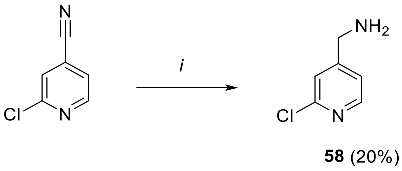

Scheme S1. Reagents and conditions: *(i)* $NaBH_4$, $CoCl_2$/DMG/DMF, THF/H_2O, rt, 3h.

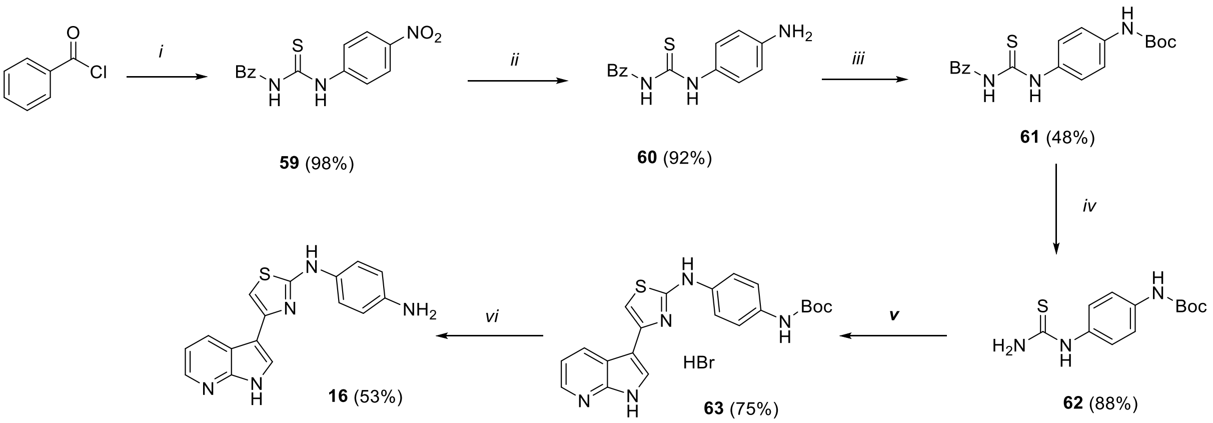

Scheme S2. Reagents and conditions: *(i)* a. NH_4SCN, acetone, MW, 15 min, 60°C; b. *p*-NO_2-aniline, MW, 15 min, 60°C; *(ii)* Fe/HCl, EtOAc/H_2O/AcOH, reflux, 2h; *(iii)* Boc_2O, K_2CO_3, THF, rt, 12h; *(iv)* K_2CO_3, H_2O/EtOH, reflux, 2h; *(v)* **44**, EtOH, reflux, 2h; *(vi)* TFA, DCM, 0°C-rt, 2h.

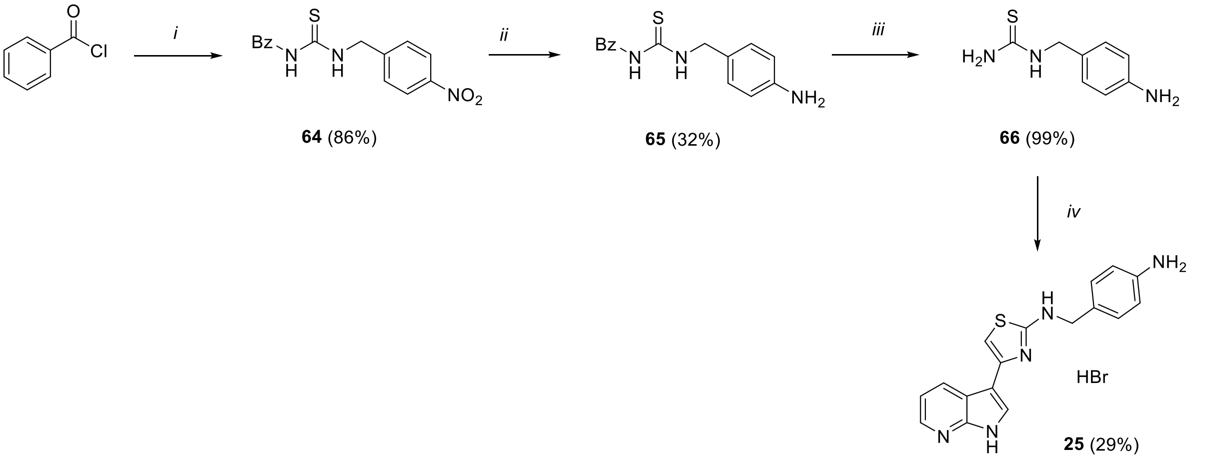

Scheme S3. Reagents and conditions: *(i)* a. NH_4SCN, acetone, MW, 15 min, 60°C; b. *p*-NO_2-benzylamine, MW, 15 min, 60°C; *(ii)* Fe/HCl, EtOAc/H_2O/AcOH, reflux, 2h; *(iii)* K_2CO_3, H_2O/EtOH, reflux, 2h; *(iv)* **44**, EtOH, reflux, 2h.

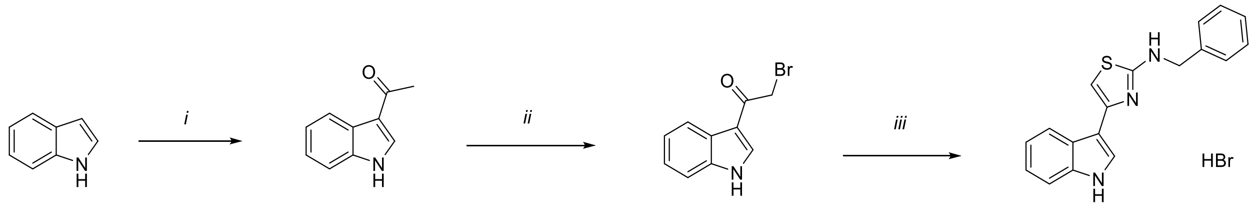

67 (49%) **68** (70%) **34** (70%)

Scheme S4. Reagents and conditions: *(i)* AcCl, $AlCl_3$, DCM, reflux, 2h; *(ii)* $CuBr_2$, MeOH, reflux, 1d; *(iii) N*-benzylthiourea, EtOH, reflux, 30 min.

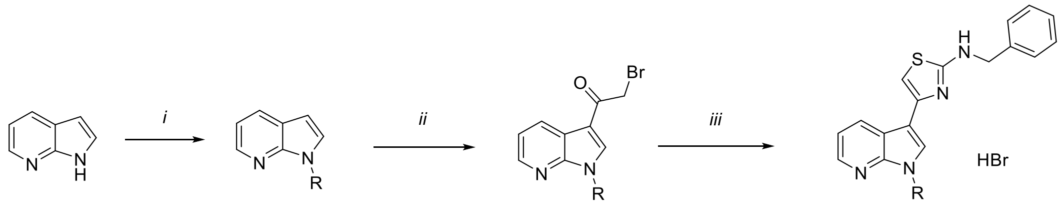

69. R=Me (60%)
70. R=allyl (70%)

71. R=Me (35%)
72. R=allyl (57%)

35. R=Me (31%)
36. R=allyl (36%)

Scheme S5. Reagents and conditions: *(i)* R-X, NaH, DMF, 0°C-rt, 3h; *(ii)* BrAcBr, $AlCl_3$, DCM, reflux, 1h; *(iii) N*-benzylthiourea, EtOH, reflux, 30 min.

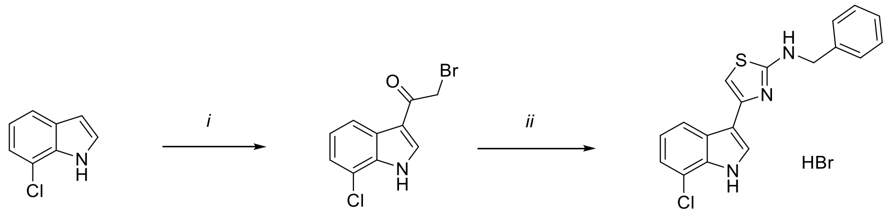

73 (34%) **37** (61%)

Scheme S6. Reagents and conditions: *(i)* BrAcBr, $AlCl_3$, DCM, reflux, 1h; *(ii) N*-benzylthiourea, EtOH, reflux, 1h.

(2-Chloropyridin-4-yl)methanamine (58)

To a solution of 2-chloroisonicotinonitrile (500 mg, 3.61 mmol) in a mixture of THF:H_2O (2:1 mL) was added a solution previously prepare including cobalt chloride (24.7 mg) and dimethylglyoxime (149.1 mg) in DMF (3 mL). After left stirring for 15 min at room temperature, $NaBH_4$ (409.7 mg, 10.83 mmol) was slowly added and the reaction is stirred for 3h under the same condition. After completion, the reaction is quenched with water (10 ml) dropwise added. After extraction with DCM (4x10 ml), the organic phases reunited were dried with Na_2SO_4, filtered and concentrated under vacuo. The resulting crude is purified through column chromatography DCM/MeOH/$NH_4OH_{(aq)}$ (9.7/0.3/0.05) to obtain **58** as yellowish solid (103 mg, 20%). ^{1}H NMR ($CDCl_3$, 400 MHz) δ 8.26 (d, *J* = 4.0Hz, 2H), 7.29 (s, 1H), 7.15 (d, *J* = 4.0Hz, 1H) 3.87 (s, 2H), 1.62 (br s, 2H). ^{13}C NMR ($CDCl_3$, 400 MHz) δ 155.38, 151.76, 149.49, 122.38, 120.84, 44.72.

2-Bromo-1-(1-methyl-1*H*-pyrrolo[2,3-*b*]pyridin-3-yl)ethan-1-one (71)

Compound **71** was obtained following general procedure A using **76** (150 mg, 1.14 mmol) and bromoacetyl bromide. Compound was eluted with DCM/MeOH (9.5/0.5) which afforded **71** as off-white solid (100 mg, 35%). ^{1}H NMR (DMSO-*d6*, 400 MHz) δ 8.69 (s, 1H), 8.48 (d, *J* = 8 Hz, 1H), 8.37 (d, *J* = 8 Hz, 1H), 7.30-7.28 (m, 1H), 4.69 (s, 2H), 3.90 (s, 3H). ^{13}C NMR (DMSO-*d6*, 400 MHz) δ 186.14, 149.92, 145.43, 138.87, 127.93, 119.70, 119.10, 111.50, 33.40, 31.69.

1-(1-Allyl-1*H*-pyrrolo[2,3-*b*]pyridin-3-yl)-2-bromoethan-1-one (72)

Compound **72** was obtained following general procedure A using **77** (260 mg, 1.64 mmol) and bromoacetyl bromide. Compound was eluted with DCM/MeOH (9.5/0.5) which afforded **72** as off-white solid (260 mg, 57%). ^{1}H NMR (DMSO-*d6*, 400 MHz) δ 8.08 (d, *J* = 2 Hz, 1H), 7.97 (d, *J* = 2Hz, 1H), 7.26-7.23 (m, 2H), 6.10-6.00 (m, 1H), 5.20-5.18 (m, 1H), 4.51-4.49 (m, 3H), 4.28 (s, 2H). ^{13}C NMR (DMSO-*d6*, 400 MHz) δ 186.12, 144.95, 135.67, 134.73, 132.17, 130.99, 119.23, 119.08, 118.89, 118.84, 47.22, 31.16.

2-Bromo-1-(7-chloro-1*H*-indol-3-yl)ethan-1-one (73)

Compound **73** was obtained following general procedure A using 7-chloroindole (300 mg, 1.98 mmol) and bromoacetyl bromide. Compound was eluted with DCM/MeOH (9.7/0.3) which afforded **73** as brown solid (180 mg, 34%). ^{1}H NMR (DMSO-*d6*, 400 MHz) δ 11.65 (br s, 1H), 7.70 (s, 1H), 7.26 (d, *J* = 8.2 Hz, 1H), 6.47 (d, *J* = 8.2 Hz, 1H), 6.38-6.34 (m, 1H), 3.85 (s, 2H). ^{13}C NMR (DMSO-*d6*, 100 MHz) δ 187.07, 136.49, 134.10, 127.81, 123.74, 123.26, 120.62, 117.09, 114.82, 34.09.

1-(1*H*-Indol-3-yl)ethan-1-one (67)

Compound **67** was obtained following general procedure A using indole (150 mg, 1.28 mmol) and acetyl chloride. Compound was eluted with DCM/MeOH/30% $NH_4OH_{(aq)}$ (9.8/0.2/0.05) which afforded **67** as off-white solid (100 mg, 49%). ^{1}H NMR (DMSO-*d6*, 400 MHz) δ 11.89 (br s, 1H), 8.26 (s, 1H), 8.14 (d, *J*=8Hz, 1H), 7.43 (d, *J*=8Hz, 1H), 7.19-7.11 (m, 2H), 2.41 (s, 3H). ^{13}C NMR (DMSO-*d6*, 100 MHz) δ 193.05, 137.06, 134.76, 125.69, 123.11, 122.04, 121.71, 117.19, 112.48, 27.67.

2-Bromo-1-(1*H*-indol-3-yl)ethan-1-one (68)

To a solution of **67** (130 mg, 0.82 mmol) in MeOH (2 mL) $CuBr_2$ (257 mg, 1.5 mmol) was added and left stirring at 50°C for 15h while monitoring with TLC. After completion, the reaction mixture was cooled down to room temperature, the obtained precipitate filtered and the obtained crude was purified by column chromatography with DCM/MeOH/30% $NH_4OH_{(aq)}$ (9.8/0.2/0.05) as mobile phase. Compound **68** was obtained as off-white solid (140 mg, 70%). ^{1}H NMR (DMSO-*d6*, 400 MHz) δ 12.13 (br s, 1H), 8.47 (s, 1H), 8.15

(d, *J*=8.6Hz 1H), 7.49 (d, *J*=8.6Hz, 1H), 7.24-7.21 (m, 2H), 4.64 (s, 2H). ^{13}C NMR (DMSO-d_6, 100 MHz) δ 186.81, 137.17, 135.71, 125.91, 123.66, 122.61, 121.65, 113.98, 112.78, 33.99.

General procedure for intermediates 59 and 64

Compounds **59 a**nd **64** were obtained following a modified general procedure B. A mixture of benzoyl chloride (1 eq) and ammonium thiocyanate (1.2 eq) was stirred in acetone (1-3 ml) under microwave irradiation (60°C, 250 psi, 80 W) for 15 min. After this time, the opportune substituted benzylamine or aniline (1 eq) was added and the mixture was irradiated for additional 15 min under the same condition. Then, the reaction mixture was filtered, the precipitate washed with acetone and the filtrate concentrated under vacuo. The resulting crude was purified by column chromatography petroleum ether/EtOAc (8/2).

***N*-((4-Nitrophenyl)carbamothioyl)benzamide (59)**

Compound **59** was obtained following general procedure for intermediates 59 and 64 using 4-nitrophenylamine (295 mg, 2.13 mmol) and affording it as a yellow solid (631 mg, 98%). ^{1}H NMR (DMSO-d_6, 400 MHz) δ 12.88 (br s, 1H), 11.78 (br s, 1H), 8.28 (d, *J* = 9.2Hz, 2H), 8.10 (d, *J* = 9.2Hz, 2H), 8.00 (d, *J* = 6.8Hz, 2H), 7.69-7.66 (m, 1H), 7.57-7.53 (m, 2H). ^{13}C NMR (DMSO-d_6, 100 MHz) δ 179.65, 168.41, 144.70, 144.51, 133.69, 132.47, 129.18 (2C), 128.90 (2C), 124.73 (2C), 124.29 (2C).

***N*-((4-Nitrobenzyl)carbamothioyl)benzamide (64)**

Compound **64** was obtained following general procedure for intermediates 59 and 64 using 4-nitrobenzylamine (400 mg, 2.63 mmol) and affording it as a yellow solid (710 mg, 86%). ^{1}H NMR ($CDCl_3$, 400 MHz) δ 11.21 (br s, 1H), 9.24 (br s, 1H), 8.18 (d, *J* = 9.2Hz, 2H), 7.82 (d, *J* = 9.2Hz, 2H), 7.61 (t, *J* = 4.4Hz, 1H), 7.53-7.48 (m, 4H), 5.02 (s, 2H). ^{13}C NMR ($CDCl_3$, 100 MHz) δ 181.03, 167.04, 147.36, 143.72, 133.70, 131.34, 129.08 (2C), 128.30 (2C), 127.45 (2C), 123.88 (2C), 48.39.

General procedure for intermediates 60 and 65

4-Nitroaryl derivatives **59** or **64** (1 eq) and iron dust (3 eq) were suspended in the solvent mixture of EtOAc/AcOH/H_2O (2/2/1, 5 ml) and 0.50 mL of concentrated HCl was added. The mixture was refluxed for 20 min and then maintained stirring at room temperature for 2 hours, monitoring the reaction by TLC. After completion, water (20 mL) was added dropwise and the resulting mixture was extracted with DCM (4x10 ml) and washed with saturated solution of $NaHCO_3$ (2x30 mL) and brine (1x30 mL). The organic phases reunited were dried with sodium sulfate, filtered and the solvent evaporated under reduced pressure. The obtained crude was further purified by column chromatography with EtOAc/petroleum ether (1/1) as mobile phase.

***N*-((4-Aminophenyl)carbamothioyl)benzamide (60)**

Compound **60** was obtained following general procedure for intermediates 60 and 65 using **59** (1.05 g, 3.48 mmol) and affording it as a yellow solid (870 mg, 92%). ^{1}H NMR (DMSO-d_6, 400 MHz) δ 12.34 (br s, 1H), 11.34 (br s, 1H), 7.93 (d, *J* = 6.8Hz, 2H), 7.63-7.60 (m, 1H), 7.52-7.48 (m, 2H), 7.26 (d, *J* =8.6Hz, 2H), 6.55 (d, *J* = 8.6Hz, 2H), 5.21 (br s, 2H). ^{13}C NMR (DMSO-d_6, 400 MHz) δ 178.65, 168.65, 147.70, 133.44, 132.69, 129.03 (2C), 128.86 (2C), 126.90, 125.72 (2C), 113.87 (2C).

***N*-((4-Aminobenzyl)carbamothioyl)benzamide (65)**

Compound **65** was obtained following general procedure for intermediates 60 and 65 using **64** (350 mg, 285.37 mmol) and affording it as a white solid (100 mg, 32%). ^{1}H NMR ($CDCl_3$, 400 MHz) δ 10.89 (br s, 1H), 9.29 (br s, 1H), 7.79 (d, *J* = 7.6 Hz, 2H), 7.56 (t, *J* = 7.6Hz, 1H), 7.47-7.43 (m, 2H), 7.16 (d, *J* = 8.0 Hz, 2H), 6.67

(d, J = 8.0Hz, 2H), 4.76 (s, 2H), 3.55 (br s, 2H). ^{13}C NMR ($CDCl_3$, 100 MHz) δ 179.52, 166.94, 146.23, 133.45, 131.79, 129.37 (2C), 129.04 (2C), 127.51 (2C), 125.93, 115.41 (2C), 49.60.

tert-Butyl (4-(3-benzoylthioureido)phenyl)carbamate (61)

Intermediate **60** (870 mg, 3.20 mmol) and potassium carbonate (660 mg, 3.84 mmol) were suspended in 3 mL of THF. Di-*tert*-butyl dicarbonate (700 mg, 3.20 mmol) was dissolved in 3 ml of THF and the obtained solution was added dropwise to the reaction flask under vigorous stirring. The mixture reacted at room temperature overnight and was monitored by TLC. After completion, water (5 ml) was added and extraction with DCM (4x10 ml) was performed. The organic phases reunited were dried with sodium sulfate, filtered and the solvent evaporated under reduced pressure. The obtained residue was purified with column chromatography using EtOAc/petroleum ether (1/1) as mobile phase to obtain the pure product as yellow solid (570 mg, 48%). ^{1}H NMR ($CDCl_3$, 400 MHz) δ 12.48 (s, 1H), 9.10 (s, 1H), 7.86 (d, J = 7.2Hz, 2H), 7.63-7.59 (m, 3H), 7.53-7.50 (m, 2H), 7.40 (d, J = 8.8Hz, 2H), 6.62 (br s, 1H), 1.50 (s, 9H). ^{13}C NMR ($CDCl_3$, 100 MHz) δ 178.24, 166.95, 152.59, 137.08, 133.70, 132.49, 131.65, 129.19 (2C), 127.48 (2C), 124.90 (2C), 118.60, 80.77, 28.30 (3C).

General procedure for intermediates 62 and 66

4-Aminoaryl derivatives **61** or **73** (1 eq) were dissolved in EtOH (3 ml) and a solution of K_2CO_3 2M (2 eq) was added dropwise at room temperature. The reaction was stirred at 75°C for 2 hours and it was monitored by TLC. After completion, the reaction was cooled down to room temperature, H_2O (9 ml) was added dropwise and extraction was performed with EtOAc (4x5 ml). The organic phases reunited were dried with sodium sulfate, filtered, concentrated and dried under vacuo to obtain the desired product.

1-(_tert_-Butyl)-3-(4-thioureidophenyl)urea (62)

Compound **62** was obtained following general procedure for intermediates 62 and 66 using **61** (80 mg, 0.22 mmol) and affording it as a yellow solid (50 mg, 88%). ^{1}H NMR (DMSO-d_6, 400 MHz) δ 9.56 (br s, 1H), 9.28 (br s, 1H), 7.36 (d, J = 8.4Hz, 2H), 7.18 (d, J = 8.4Hz, 2H), 1.43 (s, 9H). ^{13}C NMR (DMSO-d_6, 100 MHz) δ 181.43, 153.23, 136.86, 133.56, 124.67 (2C), 118.81 (2C), 79.41, 28.56 (3C).

1-(4-Aminobenzyl)thiourea (66)

Compound **66** was obtained following general procedure for intermediates 62 and 66 using **65** (200 mg, 0.70 mmol) and affording it as a white solid (125 mg, 99%). ^{1}H NMR (DMSO-d_6, 400 MHz) δ 8.23 (br s, 1H), 7.12 (br s, 2H), 6.93 (d, J = 8.0Hz, 2H), 6.47 (d, J=8.0Hz, 2H), 4.93 (s, 2H), 4.36 (br s, 2H). ^{13}C NMR (DMSO-d_6, 100 MHz) δ 183.35, 148.06, 129.34 (2C), 127.38, 114.07 (2C), 47.88.

General procedure for intermediates 69 and 70

In a pressure tube, a mixture of 7-azaindole (1 eq) and sodium hydride (1.5 eq) in DMF (1-2 mL) was stirred at 0°C for 10 min. Then, the opportune alkyl halide (1.5 eq) was added dropwise and the reaction was stirred at 60°C for 3h and monitored by TLC. After completion, the reaction was cooled down to room temperature, filtered and the filtrate was collected and concentrated under vacuo. The crude residue was purified by flash column chromatography DCM/MeOH (9.5/0.5) to obtain the pure products.

1-Methyl-1_H_-pyrrolo[2,3-_b_]pyridine (69)

Compound **69** was obtained following general procedure for intermediates **69** and **70** using methyl iodide (0.24 mL, 3.82 mmol) and affording it as white solid (202 mg, 60%). ^{1}H NMR ($CDCl_3$, 400 MHz) δ 8.33 (d, J =

4.8Hz, 1H), 7.88 (d, *J* = 8.0Hz, 1H), 7.12-7.10 (m, 1H), 6.43 (d, *J* = 3.4 Hz, 1H), 6.09 (d, *J* = 3.0Hz, 1H), 3.87 (s, 3H). ^{13}C NMR ($CDCl_3$, 100 MHz) δ 146.98, 142.90, 129.04, 126.80, 121.65, 115.54, 99.32, 30.32.

1-Allyl-1*H*-pyrrolo[2,3-*b*]pyridine (70)

Compound **70** was obtained following general procedure for intermediates **69** and **70** using allyl bromide (0.78 mL, 9.07 mmol) and affording it as white solid (670 mg, 70%). ^{1}H NMR ($CDCl_3$, 400 MHz) δ 8.30 (d, *J* = 4.8Hz, 1H), 7.91 (d, *J* = 8.0Hz, 1H), 7.21 (d, *J* = 3.5Hz, 1H), 7.07-7.04 (m, 1H), 6.50 (d, *J* = 3.5Hz, 1H), 6.08-5.99 (m, 1H), 5.24-5.04 (m, 2H), 4.95-4.93 (m, 2H). ^{13}C NMR ($CDCl_3$, 400 MHz) δ 147.39, 142.97, 133.90, 128.92, 127.81, 120.51, 117.43, 115.86, 99.75, 46.62.

***tert*-Butyl (4-((4-(1*H*-pyrrolo[2,3-*b*]pyridin-3-yl)thiazol-2-yl)amino)phenyl)carbamate hydrobromide (63)**

Compound **63** was obtained following general procedure C using **44** (72 mg, 0.30 mmol) and **62** (80 mg, 0.30 mmol), affording it as yellow solid (110 mg, 75%). ^{1}H NMR (DMSO-*d₆*, 400 MHz) δ 12.57 (br s, 1H), 10.24 (br s, 1H), 9.24 (s, 1H), 8.83 (d, *J* = 7.6Hz, 1H), 8.46 (d, *J* = 5.6Hz, 1H), 8.10 (s, 1H), 7.59 (d, *J* = 8.4Hz, 2H), 7.45-7.43 (m, 3H), 7.18 (s, 1H), 1.47 (s, 9H). ^{13}C NMR (DMSO-d_6, 100 MHz) δ 164.30, 153.34, 143.95, 143.63, 138.63, 136.17, 134.30, 133.80, 126.63, 120.57, 119.57, 118.53 (2C), 116.52 (2C), 111.59, 100.82, 79.22, 28.61 (3C).

***N^1*-(4-(1*H*-Pyrrolo[2,3-*b*]pyridin-3-yl)thiazol-2-yl)benzene-1,4-diamine (16)**

Compound **63** (110 mg, 0.23 mmol) was suspended in DCM (2 ml) and stirred at 0°C, while trifluoroacetic acid (2.95 mmol) added dropwise. The reaction was then stirred at room temperature for 2.5 hours and monitored by TLC. After completion, solvent was evaporated under reduced pressure, the obtained residue dissolved in water (2 ml) and NaOH 6N added dropwise until pH 7-8. The resulting precipitate was then filtered, washed with water and dried under vacuum to obtain the pure product **16** as yellow solid (38 mg, 53%). ^{1}H NMR (DMSO-d_6, 400 MHz) δ 11.83 (br s, 1H), 9.97 (br s, 1H), 8.44 (d, *J* = 8.0Hz, 1H), 8.27 (d, *J* = 4.0Hz, 1H), 7.91 (s, 1H), 7.56 (d, *J* = 8.8Hz, 2H), 7.17-7.14 (d, ^{1}J = 4.4Hz, ^{2}J = 8.0Hz, 1H), 6.99 (s, 1H), 6.93 (d, *J* = 8.8Hz, 2H). ^{13}C NMR (DMSO-d_6, 100 MHz) δ 164.16, 149.19, 146.18, 143.35, 135.89, 135.83, 128.70, 124.95, 119.43 (2C), 118.97 (2C), 117.41, 116.41, 110.89, 99.01. MS [ESI+] m/z: 308.20 $[M+H]^+$.

***N*-(4-Aminobenzyl)-4-(1*H*-pyrrolo[2,3-*b*]pyridin-3-yl)thiazol-2-amine hydrobromide (25)**

Compound **25** was obtained following general procedure C using **44** (131 mg, 0.55 mmol) and **66** (100 mg, 0.55 mmol), affording it as pale-yellow solid (65 mg, 29%). ^{1}H NMR (DMSO-d_6, 400 MHz) δ 11.84 (br s, 1H), 8.36 (d, *J*=8 Hz, 2H), 8.25 (d, *J*=4 Hz, 1H), 7.83 (s, 1H), 7.44 (d, *J*= 12Hz, 2H), 7.20 (d, *J*=8 Hz, 2H), 7.16-7.12 (m, 1H), 6.86 (s, 1H), 4.53 (s, 2H). ^{13}C NMR (DMSO-d_6, 100 MHz) δ 168.26, 148.55, 142.97, 136.93, 134.34, 129.32, 129.24, 124.89, 121.67, 117.70, 116.38, 110.16, 98.39, 47.94. MS [ESI+] m/z: 322.31 $[M+H]^+$.

***N*-Benzyl-4-(1*H*-indol-3-yl)thiazol-2-amine hydrobromide (34)**

Compound **34** was obtained following general procedure C using **68** (120 mg, 0.50 mmol) and *N*-benzyl thiourea (84 mg, 0.50 mmol), affording it as yellow solid (136 mg, 70%). ^{1}H NMR (DMSO-d_6, 400 MHz) δ 11.67 (br s, 1H), 9.89 (br s, 1H), 7.95 (s, 1H), 7.77 (d, *J* = 8.0Hz, 1H), 7.50-7.31 (m, 6H), 7.22-7.12 (m, 2H), 6.99 (s, 1H), 4.71 (s, 2H). ^{13}C NMR (DMSO-d_6, 100 MHz) δ 169.22, 136.80, 136.52, 129.13 (2C), 128.30, 128.22 (2C), 126.29, 124.60, 122.76, 120.79, 119.53, 112,71, 105.54, 98.66, 49.49. MS [ESI+] m/z: 306.23 $[M+H]^+$.

***N*-Benzyl-4-(1-methyl-1*H*-pyrrolo[2,3-*b*]pyridin-3-yl)thiazol-2-amine hydrobromide (35)**

Compound **35** was obtained following general procedure C using **71** (84 mg, 0.33 mmol) and *N*-benzyl thiourea (55 mg, 0.33 mmol), affording it as pale-yellow solid (41 mg, 31%). ^{1}H NMR (DMSO-d_6, 400 MHz) δ

9.59 (br s, 1H), 8.39 (d, *J* = 4.8Hz, 1H), 8.33 (d, *J* = 8.0Hz, 1H), 8.11 (s, 1H), 7.45-7.38 (m, 4H), 7.32 (t, *J* = 7Hz, 1H), 7.28-7.25 (m, 1H), 7.04 (s, 1H), 4.69 (s, 2H), 3.88 (s, 3H). ^{13}C NMR (DMSO-d_6, 100 MHz) δ 169.09, 147.05, 143.14, 137.23, 129.94, 129.45, 129.43, 129.05 (2C), 128.16 (2C), 128.10, 117.88, 116.92, 110.00, 99.56, 49.21, 31.79. MS [ESI+] m/z: 321.23 [M+H]$^+$.

4-(1-Allyl-1*H*-pyrrolo[2,3-*b*]pyridin-3-yl)-*N*-benzylthiazol-2-amine hydrobromide (36)

Compound **36** was obtained following general procedure C using **72** (176 mg, 0.63 mmol) and *N*-benzyl thiourea (105 mg, 0.63 mmol), affording it as yellow solid (97 mg, 36%). ^{1}H NMR (DMSO-d_6, 400 MHz) δ 9.60 (br s, 1H), 8.34 (dd, ^1J= 4.8 Hz, ^2J= 1.5 Hz 1H), 8.30 (dd, ^1J= 7.9 Hz, ^2J= 1.5 Hz 1H), 8.08 (s, 1H), 7.43-7.35 (m, 4H), 7.31-7.22 (m, 2H), 7.04 (s, 1H), 6.09-6.00 (m, 1H), 5.16 (dd, ^1J= 10.2 Hz, ^2J= 1.5 Hz 1H), 5.07-5.01 (m, 1H), 4.93 (d, *J*= 5.5 Hz, 2H), 4.66 (s, 2H) . ^{13}C NMR (DMSO-d_6, 100 MHz) δ 169.15, 146.88, 143.61, 137.02, 134.27 (2C), 129.30, 129.07 (2C), 128.77 (2C), 128.18, 128.17, 117.88 (2C), 117.66, 117.26, 99.84, 49.35, 46.73. MS [ESI+] m/z: 347.22 [M+H]$^+$.

***N*-Benzyl-4-(7-chloro-1*H*-indol-3-yl)thiazol-2-amine hydrobromide (37)**

Compound **37** was obtained following general procedure C using **73** (110 mg, 0.40 mmol) and *N*-benzyl thiourea (67 mg, 0.40 mmol), affording it as yellowish solid (102 mg, 61%). ^{1}H NMR (DMSO-d_6, 400 MHz) δ 12.01 (br s, 1H), 9.95 (br s, 1H), 8.05 (d, *J* = 2.4Hz, 1H), 7.74 (d, *J* = 8.0Hz, 1H), 7.46-7.36 (m, 4H), 7.32-7.27 (m, 2H), 7.13 (t, *J* = 8.0Hz, 1H), 7.05 (s, 1H), 4.70 (s, 2H). ^{13}C NMR (DMSO-d_6, 100 MHz) δ 169.17, 136.46, 133.66, 129.12, 128.31 (2C), 128.24 (2C), 127.48, 126.50, 122.34, 121.84, 118.73, 117.02, 106.77, 99.76, 49.51. MS [ESI+] m/z: 340.19 [M+H]$^+$.

Figures and Tables

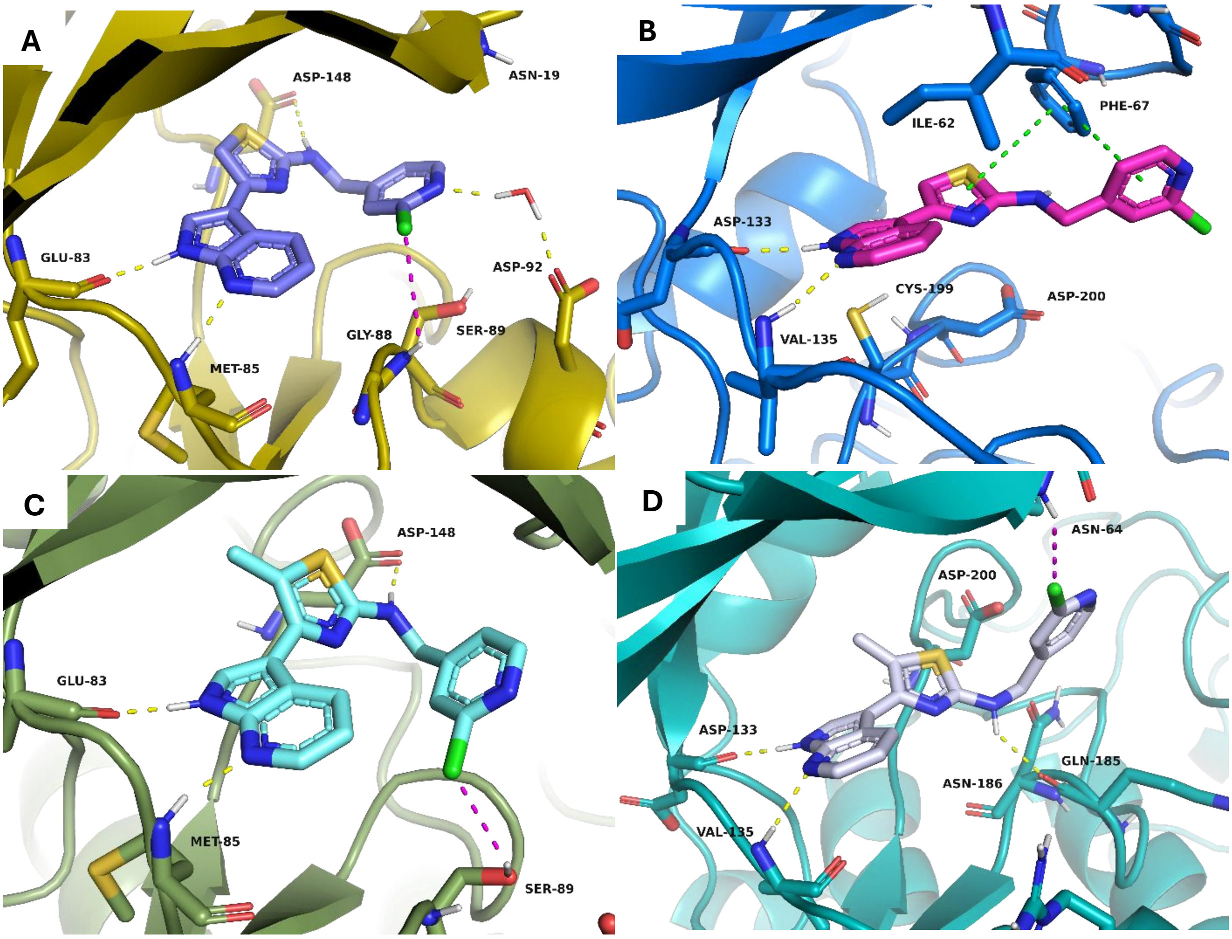

Figure S1. Tridimensional binding poses of compounds **28** (A-B) and **43** (C-D) within Fyn (A-C) and GSK-3*β* (B-D) binding site with highlighted established interactions. Yellow dotted lines are H-bond interactions, magenta dotted line is the interaction between halogen and H-bond donors and green dotted line is p-p interaction.

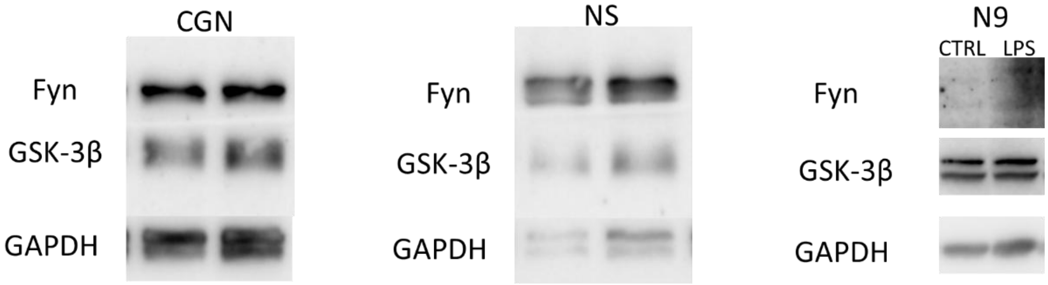

Figure S2. Western blot analysis of GSK-3*β* and Fyn kinases expression in CGNs, NS and N9 microglial cell lines. GAPDH was used as loading control.

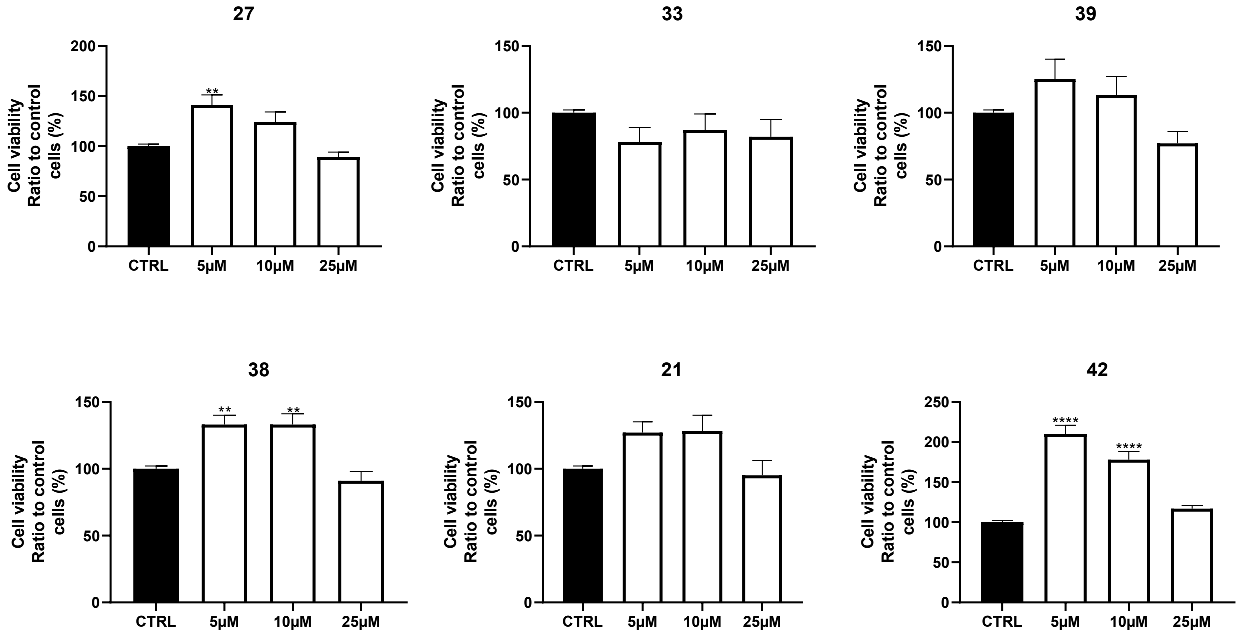

Figure S3. Neurotoxicity of selected compounds at 5, 10 and 25 μM on CGNs, expressed as percentage of cell viability compared to control. n=4; ****p<0.01*, *****p<0.0001* vs CTRL, One-way ANOVA, Dunnett's Multiple Comparison Test.

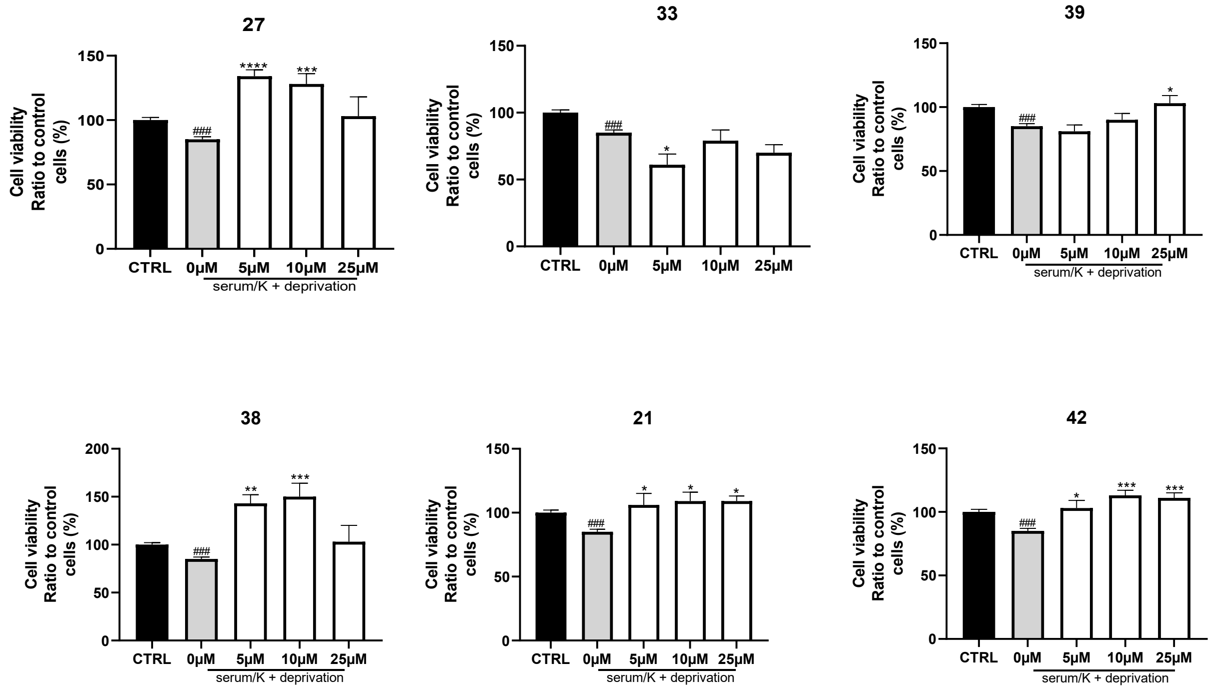

Figure S4. Neuroprotection of selected compounds at 5, 10 and 25 μM on CGNs, expressed as percentage of cell viability compared to control. n=4; * vs no serum no K. One-way ANOVA, Dunnett's Multiple Comparison Test; # vs CTRL; unpaired t-test. **p<0.05*, ***p<0.01*, ****p<0.001*, *****p<0.0001* vs CTRL.

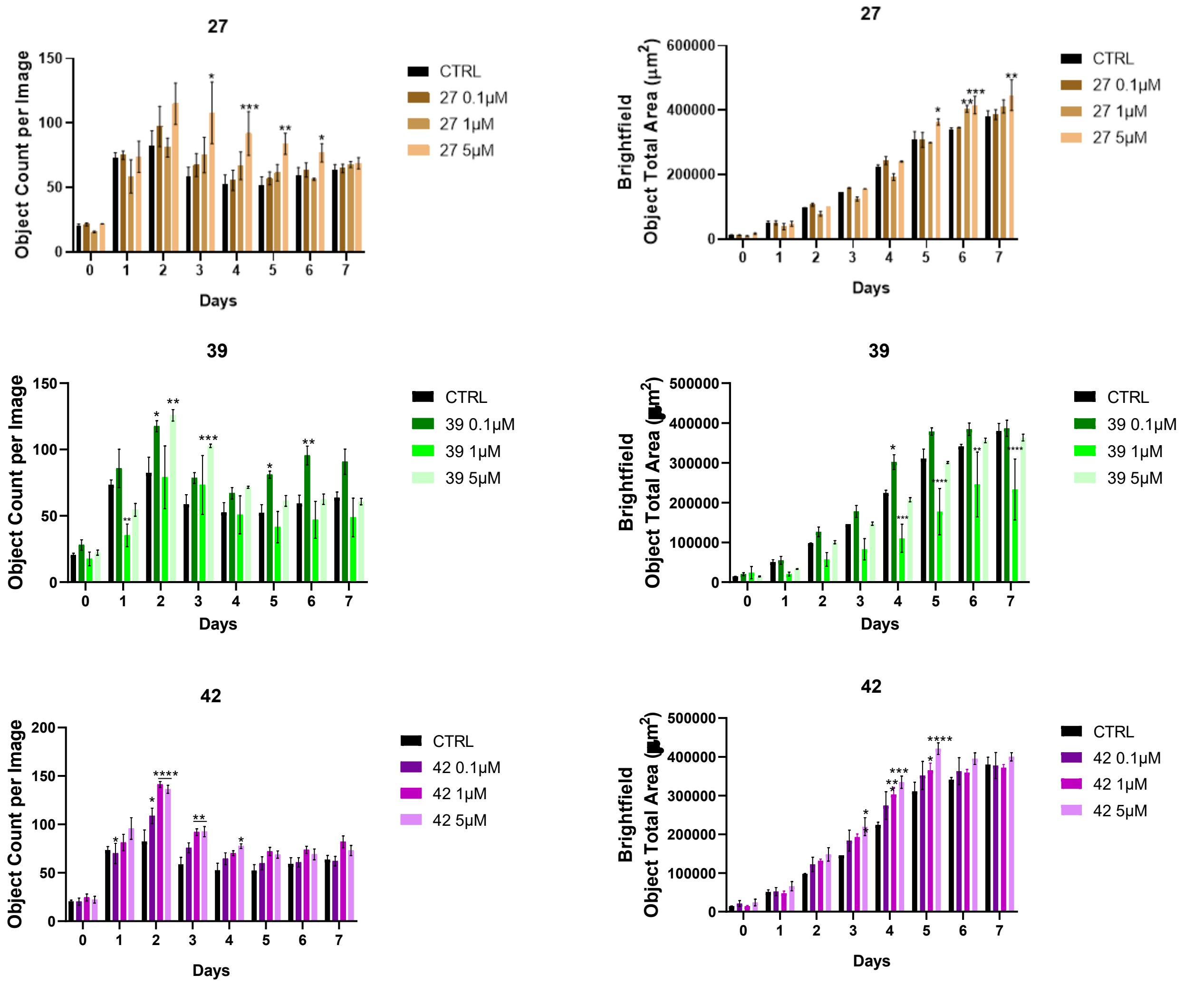

Figure S5. Growth analysis of neurospheres through the analysis of the number of objects per image (on the left) and the total of the area of the brightfield object in the image (on the right). Single neurosphere were plated (5000 per well) and let spontaneously grow for 7 days. Tested concentrations: 0.1, 1 and 5 μM. Images were acquired every 24 h. N=4 ± SE. Two-way ANOVA, Dunnett's Multiple Comparison test. *$p<0.05$*, **$p<0.01$*, ***$p<0.001$*, ****$p<0.0001$* vs CTRL.

Table S1. Physicochemical parameters of compounds **28** and **43** (calculated as hydrobromide salts).[a]

	Compound 28	Compound 43	Optimal Physicochemical properties for CNS drug[b]
**cLogP**	3.40	4.05	2<cLogP<4
**cLogD**	3.40	4.05	2<cLogD<3
**MW**	422.73	436.76	<450
**TPSA**	67.74	67.74	<90
**HBD**	3	3	<3
**HBA**	2	2	<7
**pKa**	3.44	3.66	6<pKa<10.5
**Num. rotatable bonds**	4	4	<8
**CNS MPO score**	4.35	3.73	>4

[a] Properties computed by MarvinSketch21.3 and SwissADME (*Sci. Rep.* 2017, 7:42,717).

[b] Data taken from *J. Med. Chem*. 2021, 64, 18, 13,152–13173; *J. Med. Chem*. 2015, 58, 2584-2608; *ACS Chem. Neurosci*. 2016, 7, 767-775.

Table S2. Prediction of the BBB penetration expressed as the permeability coefficient (Papp, cm s-1) ± SE. Antypirine and Donepezil were used as positive controls, while TRITC-Dextran and Inulin as negative controls.

**Compound**	**Papp (10^{-6} cm/s)**	**CNS (+/-)**
**28**	**12.4 ± 1.13**	**+/-**
**43**	**32 ± 5.38**	**+**
**Positive control**	**Papp (10^{-6} cm/s)**	**CNS (+)**
**Antypirine**	**28.0± 0.53**	**+**
**Donepezil**	**15.6± 3.1**	**+**
**Negative control**	**Papp (10^{-6} cm/s)**	**CNS (-)**
**Inulin**	**6.74± 1.7**	**-**
**TRITC-dextran**	**6.77 ± 1.08**	**-**

Table S3. Kinase inhibition profiles of compounds **41** and **43** over a panel of 58 kinases at 10 µM.

Kinase	43	41
	% Residual activity	
Abl(h)	6	8
ALK(h)	61	38
AMPKα1(h)	60	54
ASK1(h)	97	72
Aurora-A(h)	27	11
CaMKI(h)	80	89
CDK1/cyclinB(h)	20	27
CDK2/cyclinA(h)	16	27
CDK6/cyclinD3(h)	90	86
CDK7/cyclinH/MAT1(h)	62	55
CDK9/cyclin T1(h)	5	50
CHK1(h)	112	111
CK1γ1(h)	77	93
CK2α2(h)	100	83
c-RAF(h)	34	52
DRAK1(h)	14	9
eEF-2K(h)	108	121
EGFR(h)	86	50
EphA5(h)	103	93
EphB4(h)	108	109
Fyn(h)	0	-2
GSK3β(h)	17	58
IGF-1R(h)	68	83
IKKα(h)	99	96
IRAK4(h)	31	52
JAK2(h)	46	26
KDR(h)	15	16
LOK(h)	4	4
Lyn(h)	1	1
MAPKAP-K2(h)	45	33
MEK1(h)	35	61
MLK1(h)	12	8
Mnk2(h)	27	17
MSK2(h)	1	59
MST1(h)	14	16
mTOR(h)	97	86
NEK2(h)	84	56
p70S6K(h)	14	50
PAK2(h)	72	75
PDGFRβ(h)	93	95
Pim-1(h)	55	20
PKA(h)	8	111
PKBα(h)	103	104
PKCα(h)	28	66
PKCθ(h)	23	98
PKG1α(h)	40	80
Plk3(h)	96	89
PRAK(h)	97	64
ROCK-I(h)	76	93

**Rse(h)**	98	34
**Rsk1(h)**	3	44
**SAPK2a(h)**	103	101
**SRPK1(h)**	96	85
**TAK1(h)**	30	49
**PI3 Kinase (p110β/p85α)(h)**	94	89
**PI3 Kinase (p120γ)(h)**	94	80
**PI3 Kinase (p110δ/p85α)(h)**	97	66
**PI3 Kinase (p110α/p85α)(h)**	96	97

NMR spectra and UHPLC traces of final compounds 26, 28, 40, 41 and 43

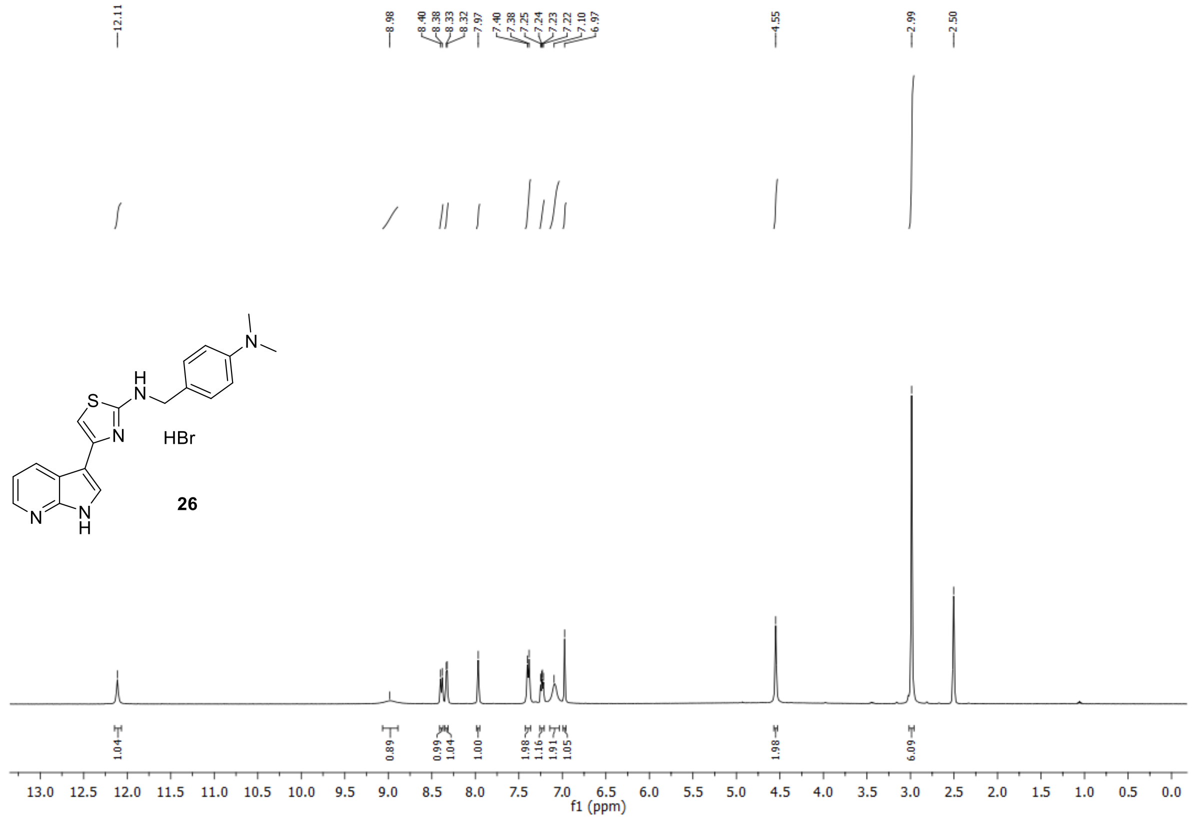

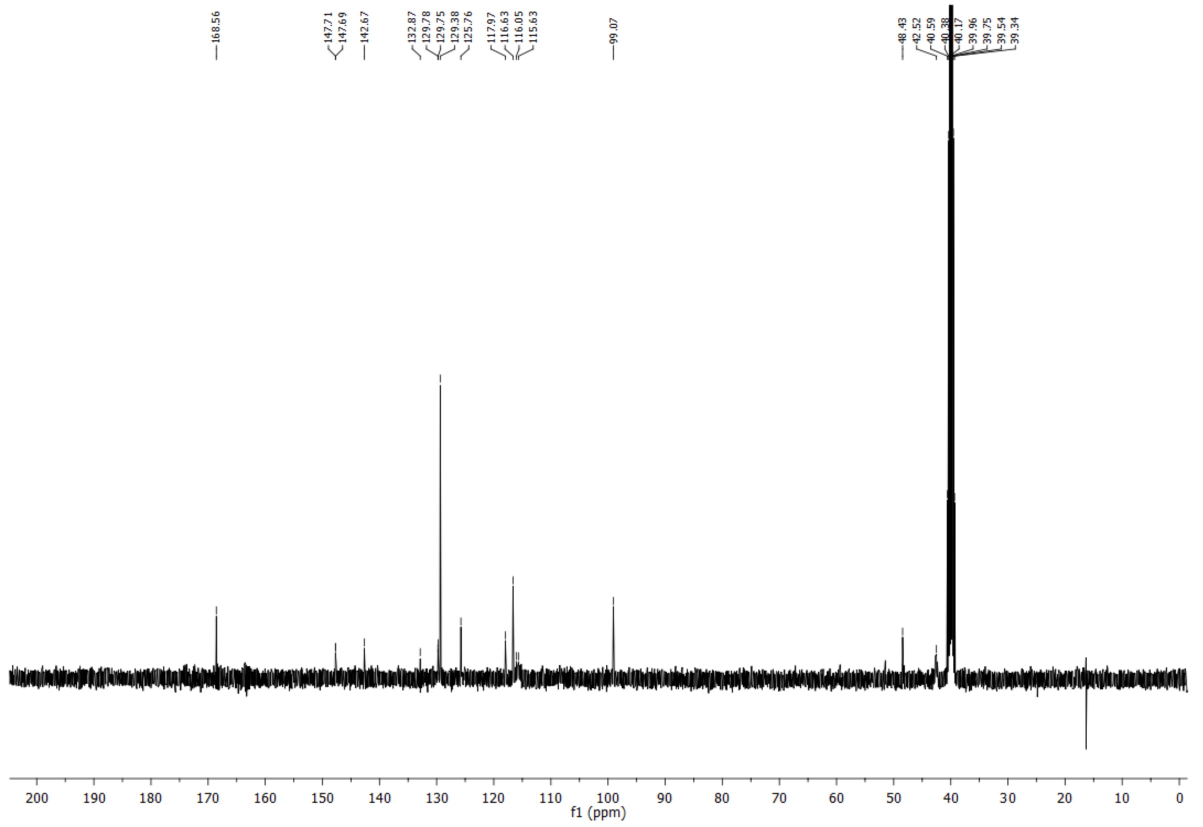

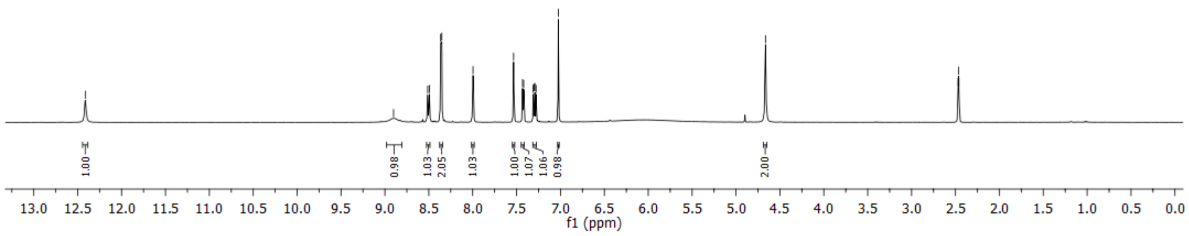
12.41
8.90
8.51
8.49
8.36
8.35
8.00
7.53
7.43
7.42
7.31
7.30
7.29
7.28
7.02
4.66
2.47
2.47
2.46
1.00
0.98
1.03
2.05
1.03
1.00
1.07
1.06
0.98
2.00
f1 (ppm)

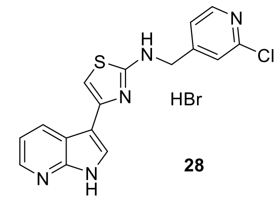
HBr

28

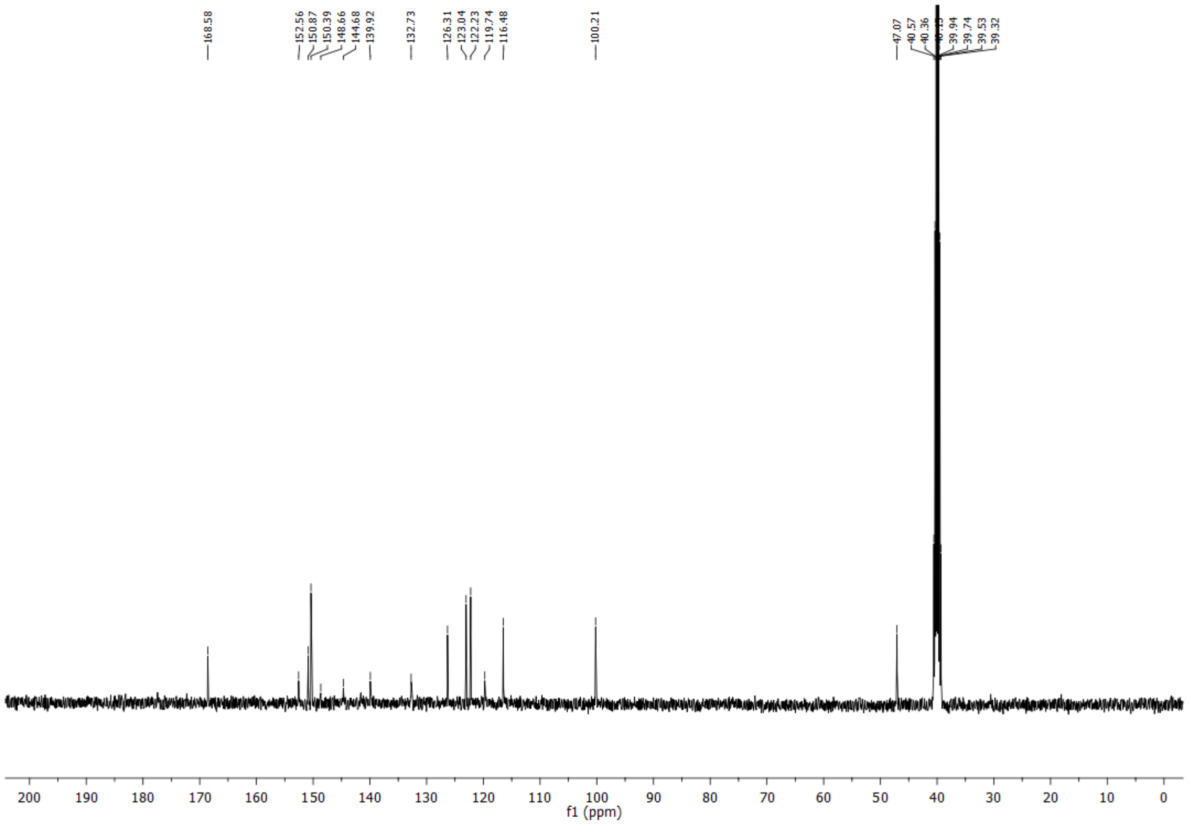
168.58
152.56
150.87
150.39
148.66
144.68
139.92
132.73
126.31
123.04
122.23
119.74
116.48
100.21
47.07
40.57
40.36
39.94
39.74
39.53
39.32
f1 (ppm)

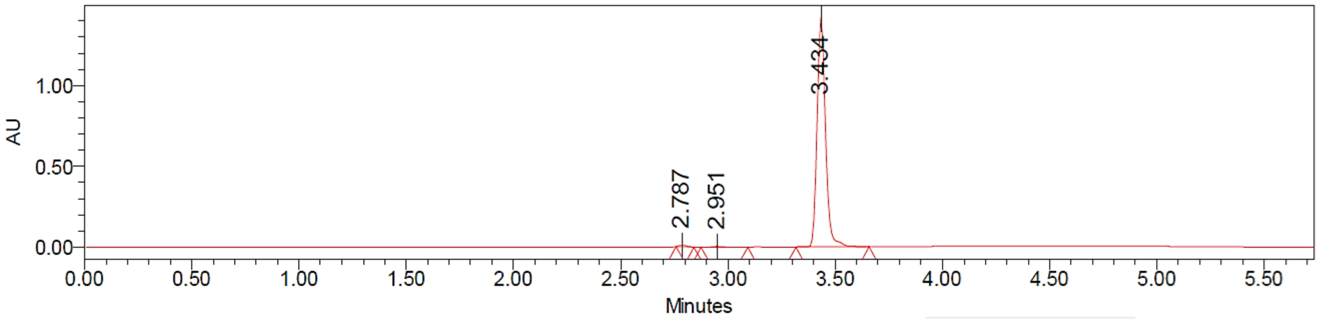

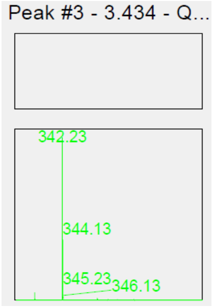

	Processed Channel	Retention Time (min)	Area	% Area	Height
1	W2489 ChA 254nm	2.787	20402	0.52	9038
2	W2489 ChA 254nm	2.951	10110	0.26	3620
3	W2489 ChA 254nm	3.434	3856568	99.22	1401812

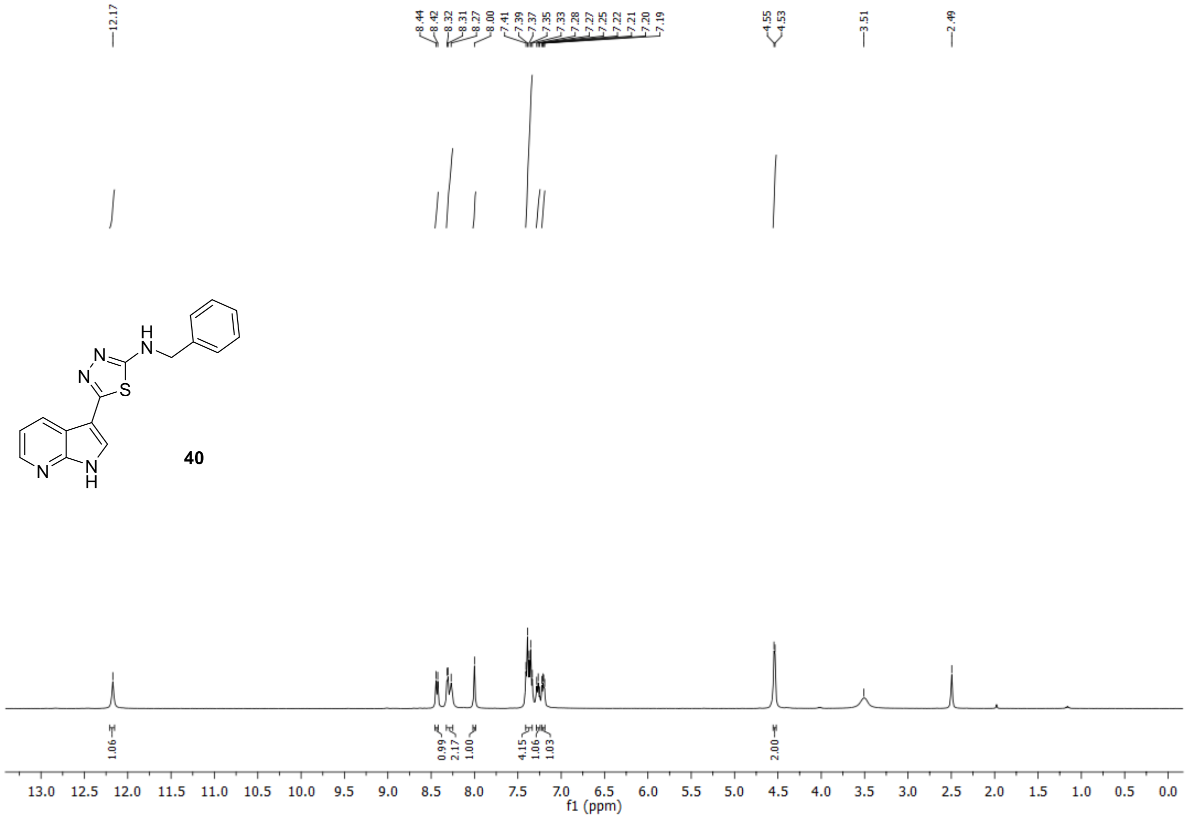
12.17
8.44 8.42 8.32 8.31 8.27 8.00 7.41 7.39 7.37 7.35 7.33 7.28 7.27 7.25 7.22 7.21 7.20 7.19
4.55 4.53
3.51
2.49
40
f1 (ppm)

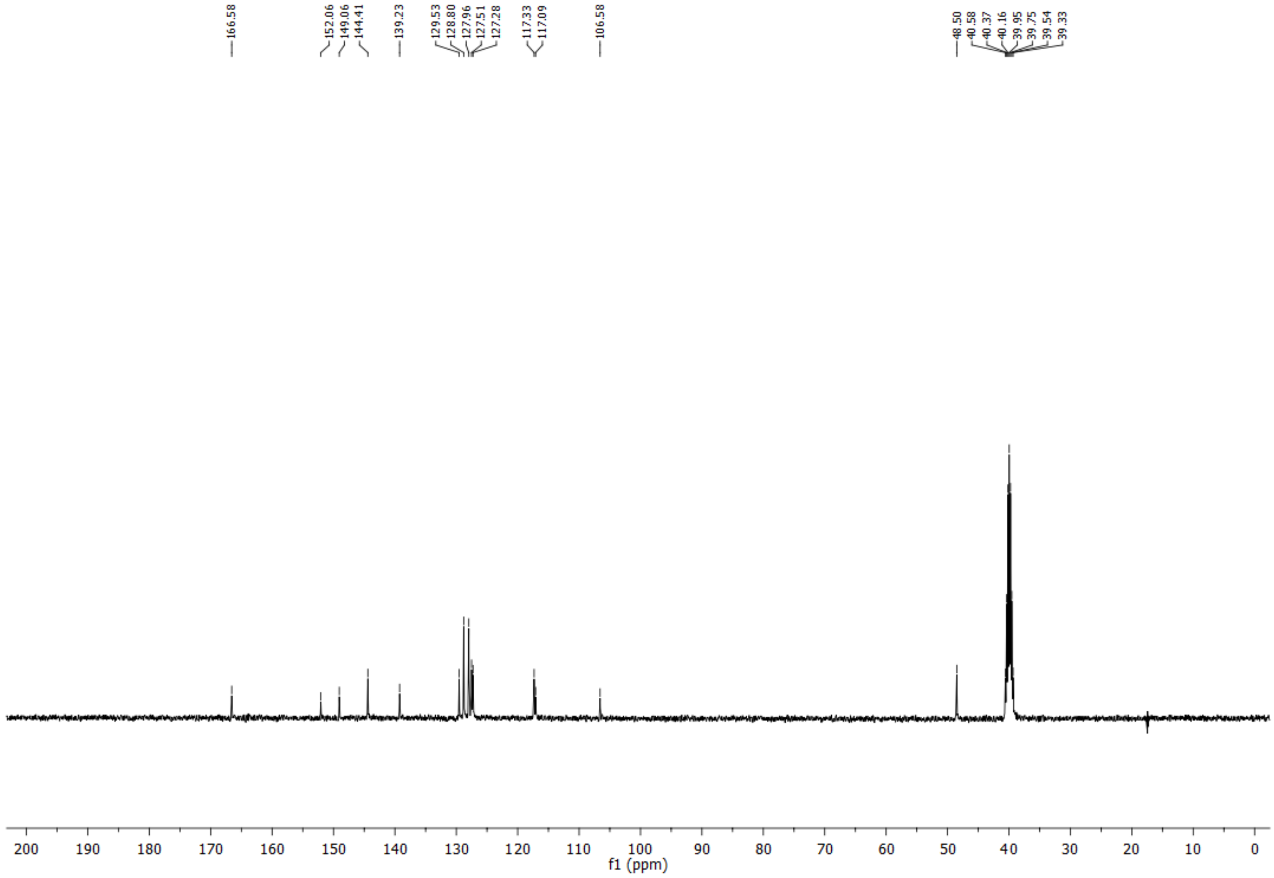
166.58
152.06 149.06 144.41
139.23
129.53 128.80 127.96 127.51 127.28
117.33 117.09
106.58
48.50 40.58 40.37 40.16 39.95 39.75 39.54 39.33
f1 (ppm)

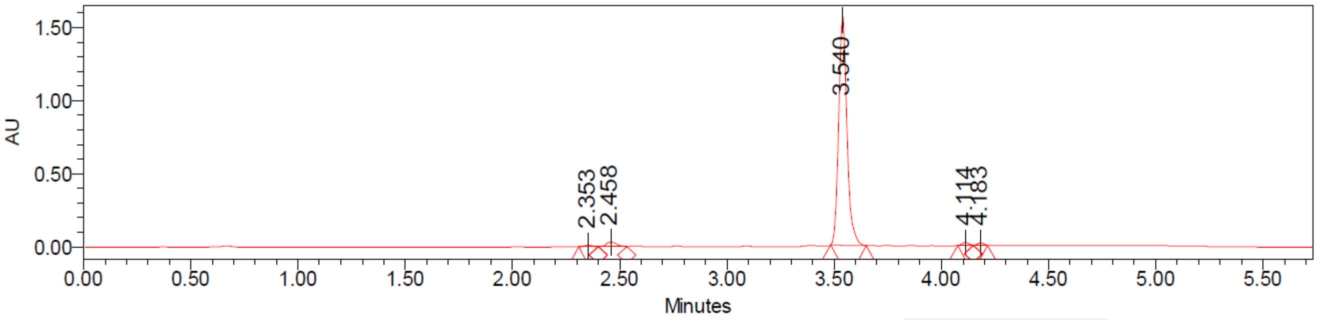

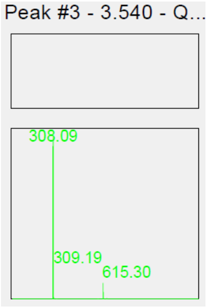

	Processed Channel	Retention Time (min)	Area	% Area	Height
1	W2489 ChA 254nm	2.353	23689	0.54	9752
2	W2489 ChA 254nm	2.458	108483	2.45	31797
3	W2489 ChA 254nm	3.540	4207120	95.11	1546461
4	W2489 ChA 254nm	4.114	47054	1.06	20409
5	W2489 ChA 254nm	4.183	36894	0.83	17283

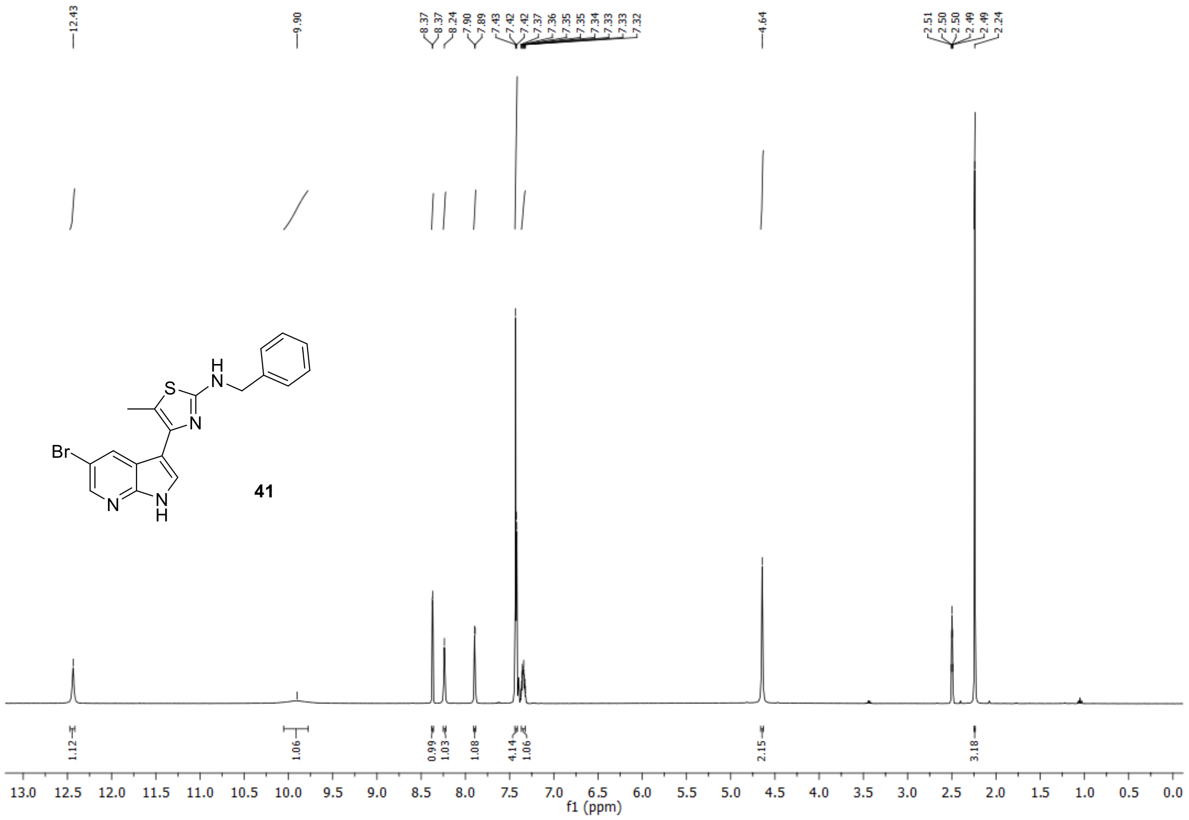
12.43
9.90
8.37
8.37
8.24
7.90
7.89
7.43
7.42
7.42
7.37
7.36
7.35
7.35
7.34
7.33
7.33
7.32
4.64
2.51
2.50
2.50
2.49
2.49
2.24
Br
N
H
S
N
H
N
41
1.12
1.06
0.99
1.03
1.08
4.14
1.06
2.15
3.18
13.0 12.5 12.0 11.5 11.0 10.5 10.0 9.5 9.0 8.5 8.0 7.5 7.0 6.5 6.0 5.5 5.0 4.5 4.0 3.5 3.0 2.5 2.0 1.5 1.0 0.5 0.0
f1 (ppm)

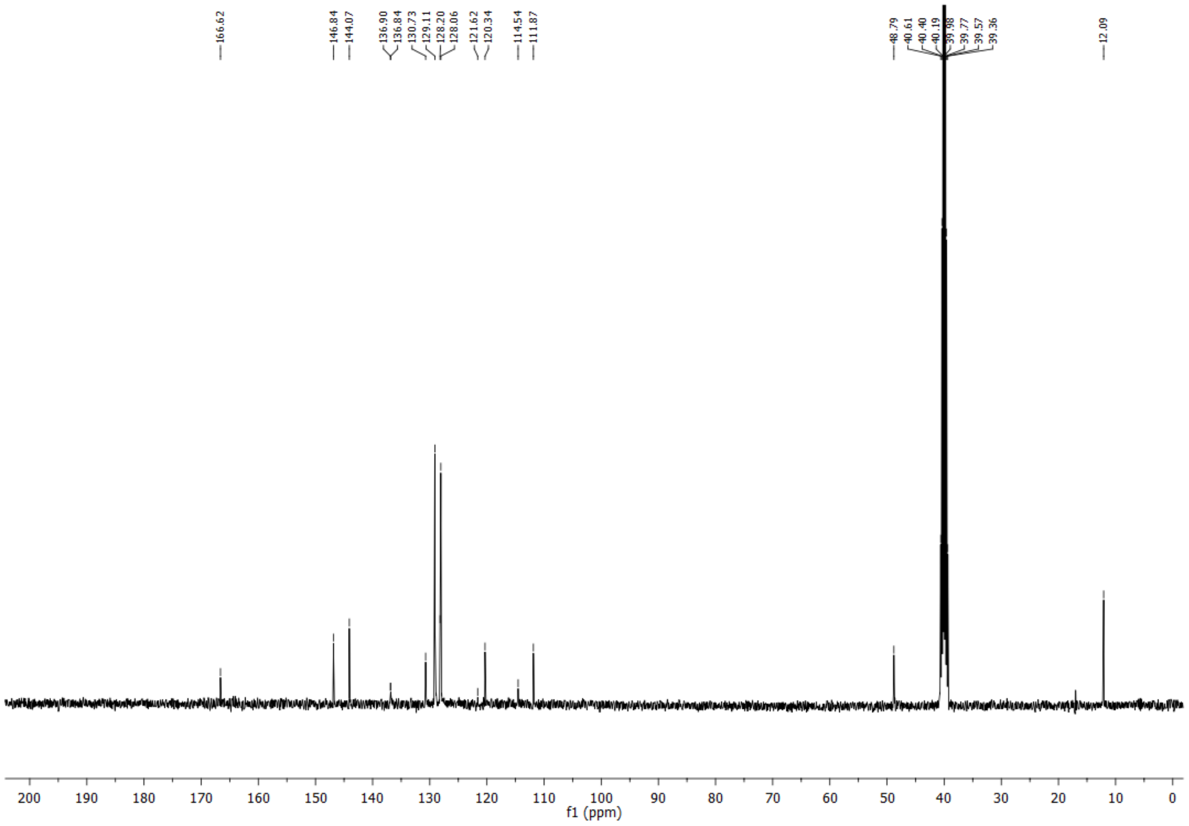
166.62
146.84
144.07
136.90
136.84
130.73
129.11
128.20
128.06
121.62
120.34
114.54
111.87
48.79
40.61
40.40
40.19
39.98
39.77
39.57
39.36
12.09
200 190 180 170 160 150 140 130 120 110 100 90 80 70 60 50 40 30 20 10 0
f1 (ppm)

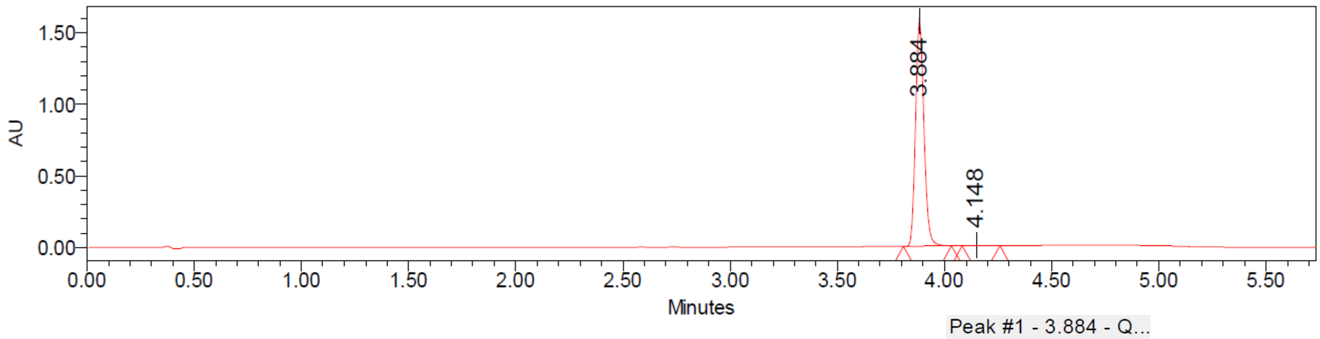

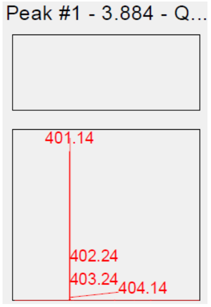

	Processed Channel	Retention Time (min)	Area	% Area	Height
1	W2489 ChA 254nm	3.884	4304018	99.87	1574736
2	W2489 ChA 254nm	4.148	5731	0.13	1921

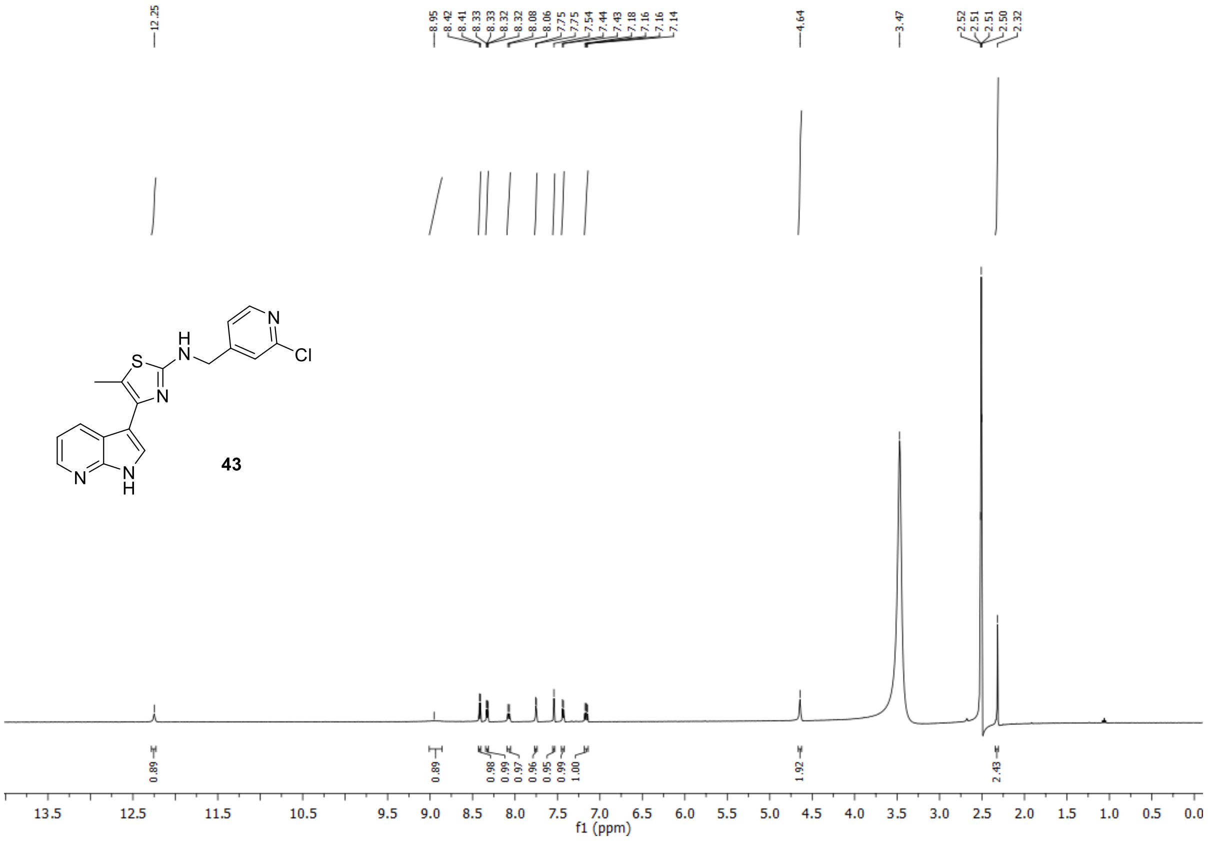
12.25
8.95
8.42
8.41
8.33
8.33
8.32
8.32
8.08
8.06
7.75
7.75
7.54
7.44
7.43
7.18
7.16
7.16
7.14
4.64
3.47
2.52
2.51
2.51
2.50
2.32
H
N
S
N
N
Cl
N
N
H
43
0.89
0.89
0.98
0.99
0.97
0.96
0.95
0.99
1.00
1.92
2.43
13.5
12.5
11.5
10.5
9.5
9.0
8.5
8.0
7.5
7.0
6.5
6.0
5.5
5.0
4.5
4.0
3.5
3.0
2.5
2.0
1.5
1.0
0.5
0.0
f1 (ppm)

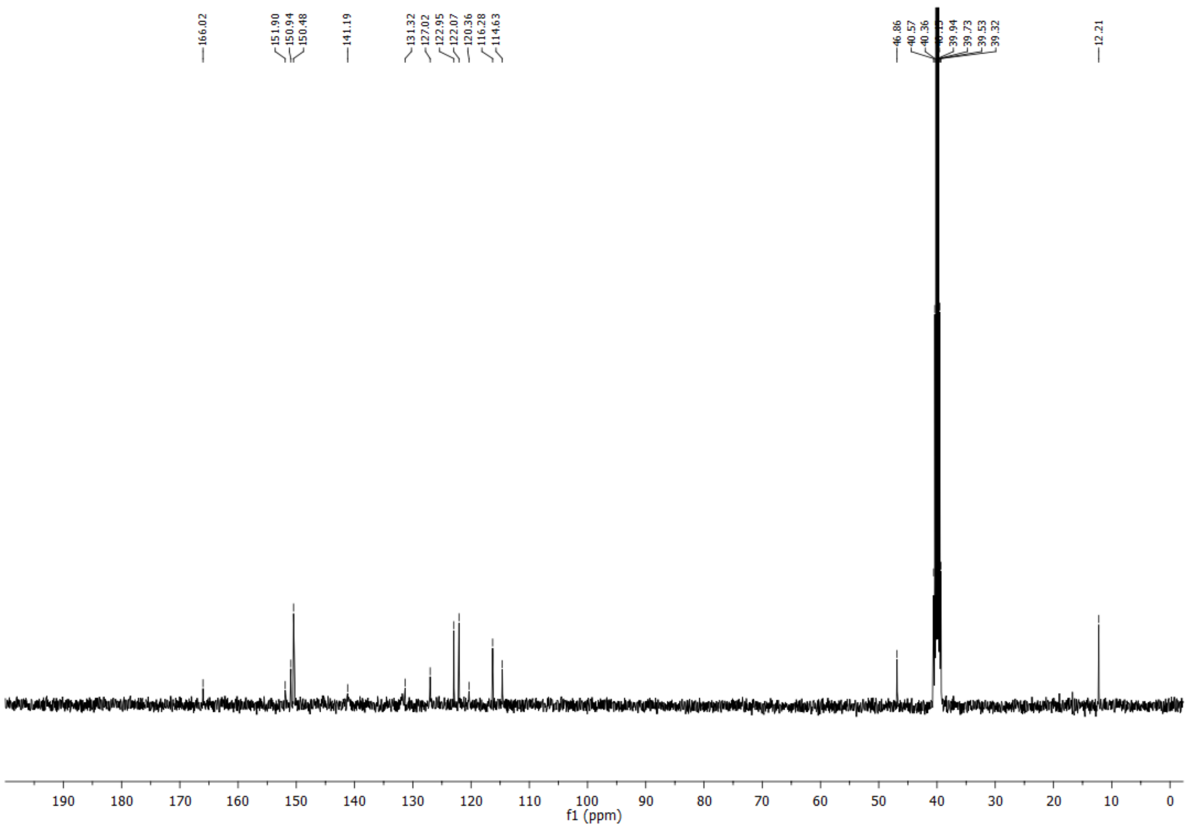
166.02
151.90
150.94
150.48
141.19
131.32
127.02
122.95
122.07
120.36
116.28
114.63
46.86
40.57
40.36
39.94
39.73
39.53
39.32
12.21
190
180
170
160
150
140
130
120
110
100
90
80
70
60
50
40
30
20
10
0
f1 (ppm)

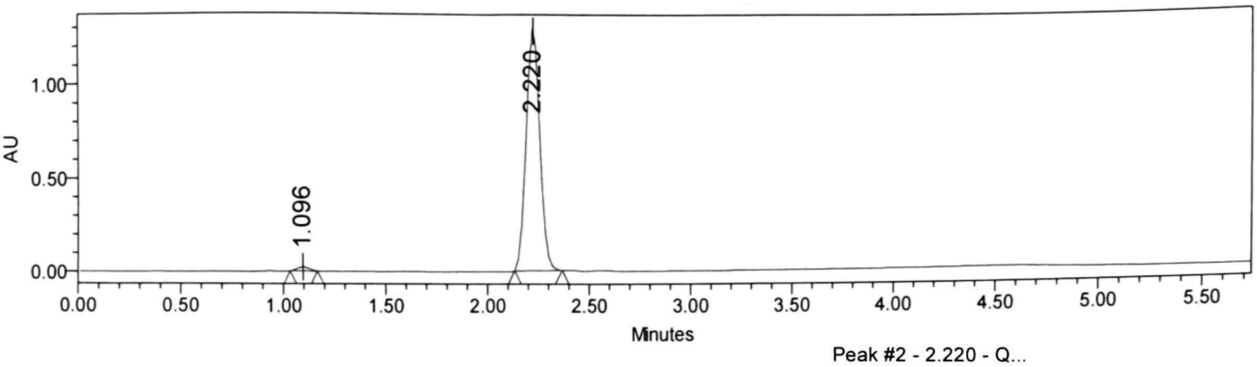

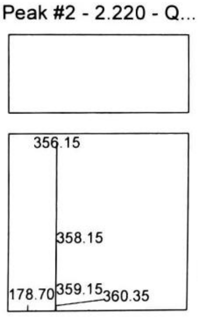

	Processed Channel	Retention Time (min)	Area	% Area	Height
1	W2489 ChA 254nm	1.096	85716	1.40	21508
2	W2489 ChA 254nm	2.220	6019524	98.60	1281002
